# Supplementary material for: α-Catenin levels determine direction of YAP/TAZ response to autophagy perturbation
Source: Nat Commun. 2021 Mar 17;12:1703. doi: 10.1038/s41467-021-21882-1 (PMC7969950; doi:10.1038/s41467-021-21882-1)
Supplement: Supplementary file 1 — Supplementary Information [file 41467_2021_21882_MOESM1_ESM.pdf]

Supplementary Information

α-Catenin Levels Determine Direction of YAP/TAZ Response to Autophagy Perturbation

Mariana Pavel[1,2†], So Jung Park[1,3†], Rebecca A. Frake[1], Sung Min Son[1,3], Marco M. Manni[1,3], Carla F. Bento[1], Maurizio Renna[1], Thomas Ricketts[1], Fiona M. Menzies[1], Radu Tanasa[4] & David C. Rubinsztein[1,3*]

Supplementary Fig. 1

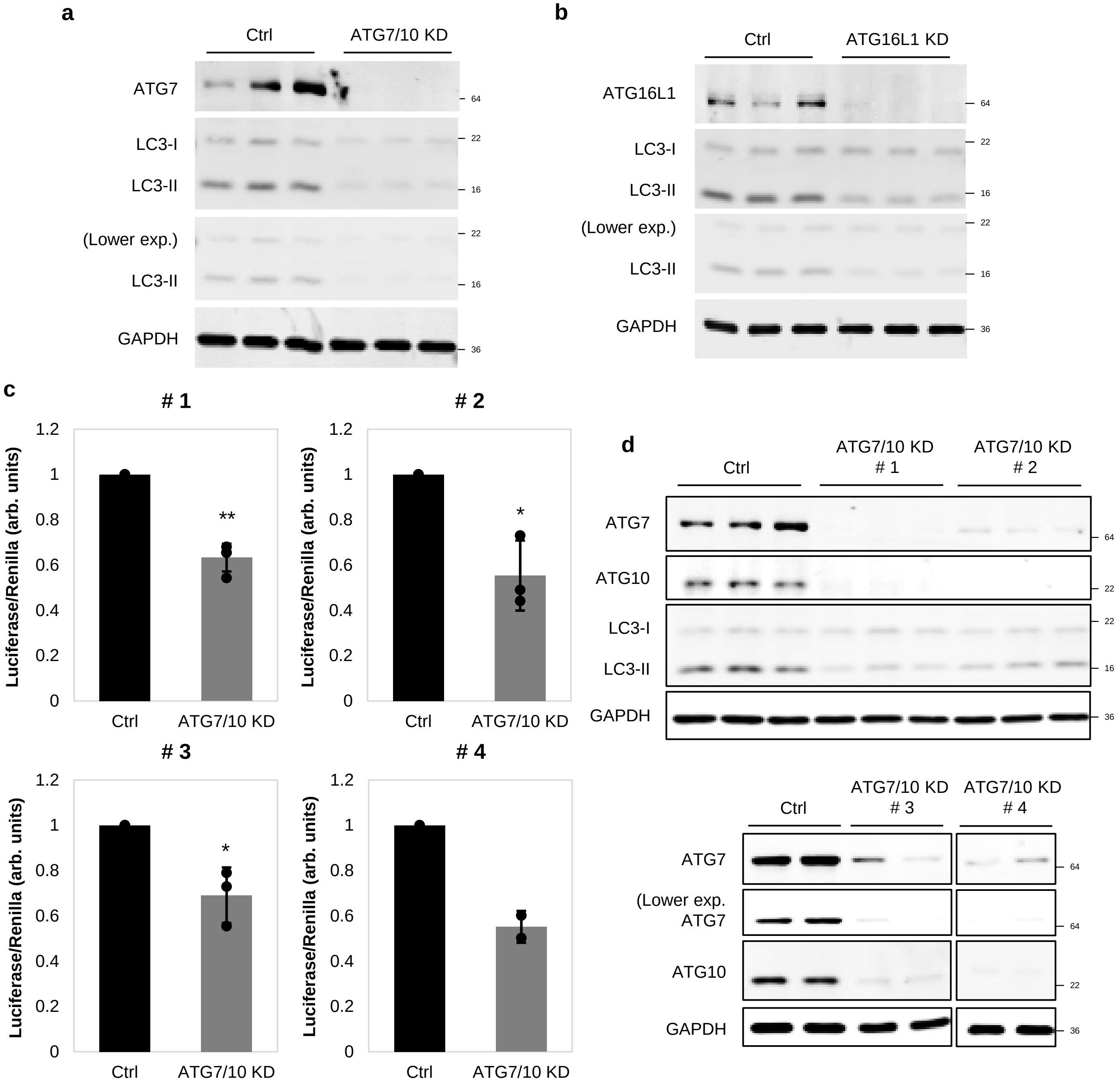

Supplementary Fig.1 | TEAD activity is reduced in autophagy-inhibited MCF10A cells

a, Representative ATG7/10 immunoblot in MCF10A cells exposed to control or ATG7/10 (pool of 4 oligos) siRNAs. The experiment was repeated 3 times with similar results.

b, Representative ATG16L1 immunoblot in MCF10A cells exposed to control or ATG16L1 (pool of 4 oligos) siRNAs. The experiment was repeated 3 times with similar results.

c, TEAD luciferase activity in MCF10A cells exposed to control or various combinations of single oligos of ATG7 and ATG10 siRNAs. Bars represent the mean ± s.d. (#1: $n = 4$, #2: $n = 3$, #3: $n = 3$, #4: $n = 2$, where n represents the number of independent experiments; $^{**}P < 0.01$, $^{*}P < 0.05$; two-tailed one sample t-test).

d, Representative ATG7/10 immunoblot in MCF10A cells treated as in **c** (the total number of independent experiments per each condition is reported in **c**).

Exact *P* values for asterisks: **c** **#1** 0.0012; **#2** 0.0379; **#3** 0.0486, **#4** 0.0700.

Supplementary Fig. 2

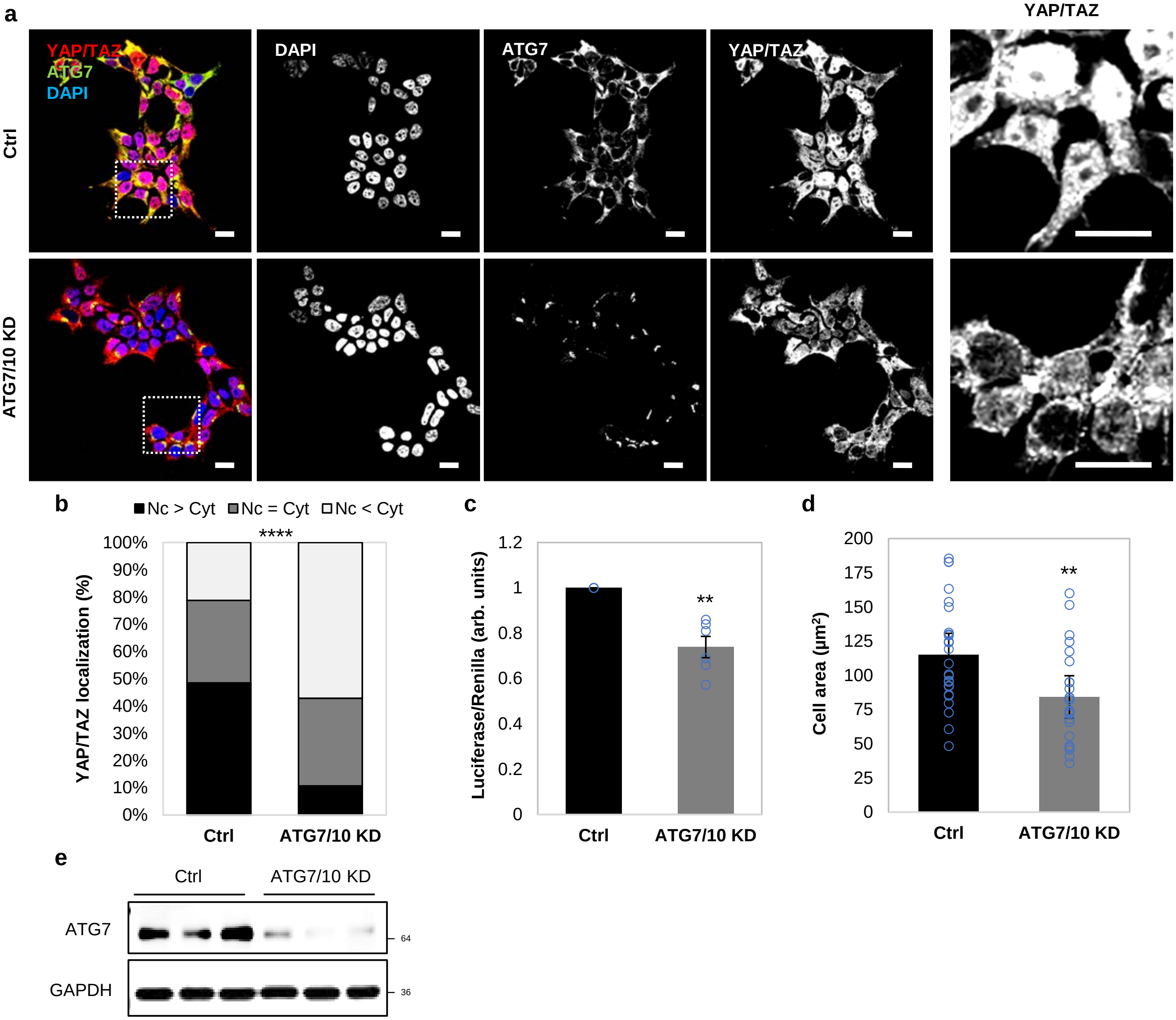

Supplementary Fig. 2 | YAP/TAZ activity is reduced in autophagy-inhibited HEK293T cells

a, Representative confocal images of YAP/TAZ immunostaining in HEK293T cells exposed to control or ATG7/10 siRNAs. Scale bars are 10 µm. The experiment was repeated 2 times with similar results.

b, YAP/TAZ localization (nuclear – Nc or cytoplasmic – Cyt) in HEK293T cells exposed to control or ATG7/10 siRNAs (****$P < 0.0001$; chi-squared test).

c, TEAD luciferase activity in HEK293T cells exposed to control or ATG7/10 siRNAs. Bars represent the mean ± s.e.m. ($n = 6$ independent experiments; **$P < 0.01$; two-tailed one sample t-test).

d, Size of HEK293T cells exposed to control or ATG7/10 siRNAs. Confocal images of HEK293T cells were analyzed for cell area using ZEN software. Bars represent the mean ± s.e.m (n = 27 (Ctrl) and 25 (ATG7/10KD) cells over 2 independent experiments; **$P < 0.01$; two-tailed t-test).

e, Representative ATG7 immunoblot in HEK293T cells treated as above. The experiment was repeated 2 times with similar results.

Exact P values for asterisks: **c** 0.0026; and **d** 0.0025.

Supplementary Fig. 3

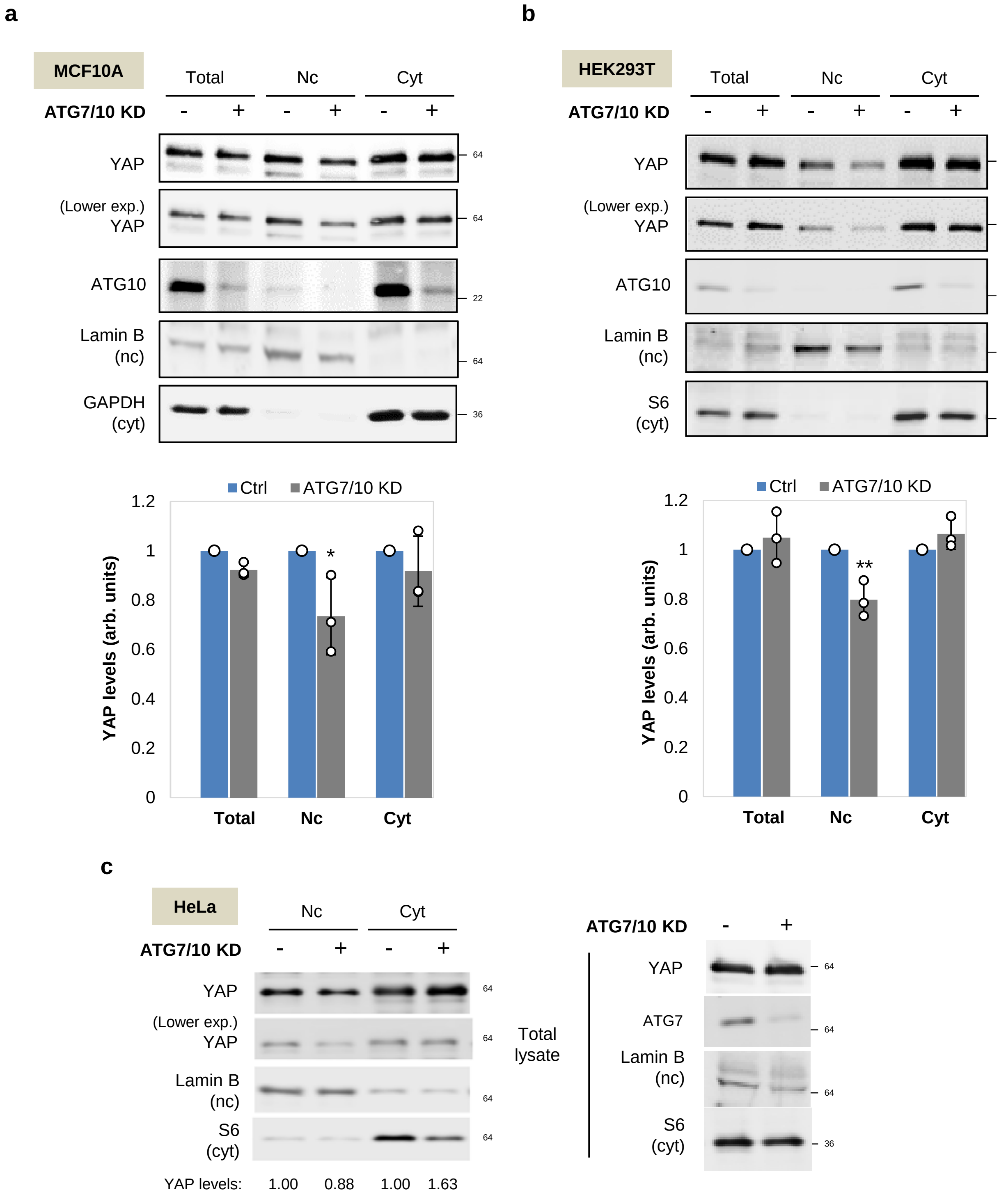

Supplementary Fig. 3 | YAP/TAZ nuclear to cytoplasmic translocation upon ATG7/10 depletion in MCF10A, HEK293T, HeLa cells.

a, Representative immunoblot of YAP subcellular nuclear (Nc) and cytoplasmic (Cyt) fractions from control (scramble) and ATG7/10 knockdown MCF10A cells. Lamin B and GAPDH were used as loading controls for the nuclear and cytoplasmic fractions, respectively. Bars represent the mean ± s.d. . (*n* = 3 independent experiments; **P* < 0.05; two-tailed one sample t-test).

b, Representative immunoblot of YAP subcellular nuclear (Nc) and cytoplasmic (Cyt) fractions from control (scramble) and ATG7/10 knockdown HEK293T cells. Lamin B and S6 were used as loading controls for the nuclear and cytoplasmic fractions, respectively. Bars represent the mean ± s.d. (*n* = 3 independent experiments; ***P* < 0.01; two-tailed one sample t-test).
c, Representative immunoblot of YAP subcellular nuclear (Nc) and cytoplasmic (Cyt) fractions from control (scramble) and ATG7/10 knockdown HeLa cells. Lamin B and S6 were used as loading controls for the nuclear and cytoplasmic fractions, respectively. The experiment was repeated 3 times with similar results.
Exact *P* values for asterisks: **a** 0.0457; and **b** 0.0083.

Supplementary Fig. 4

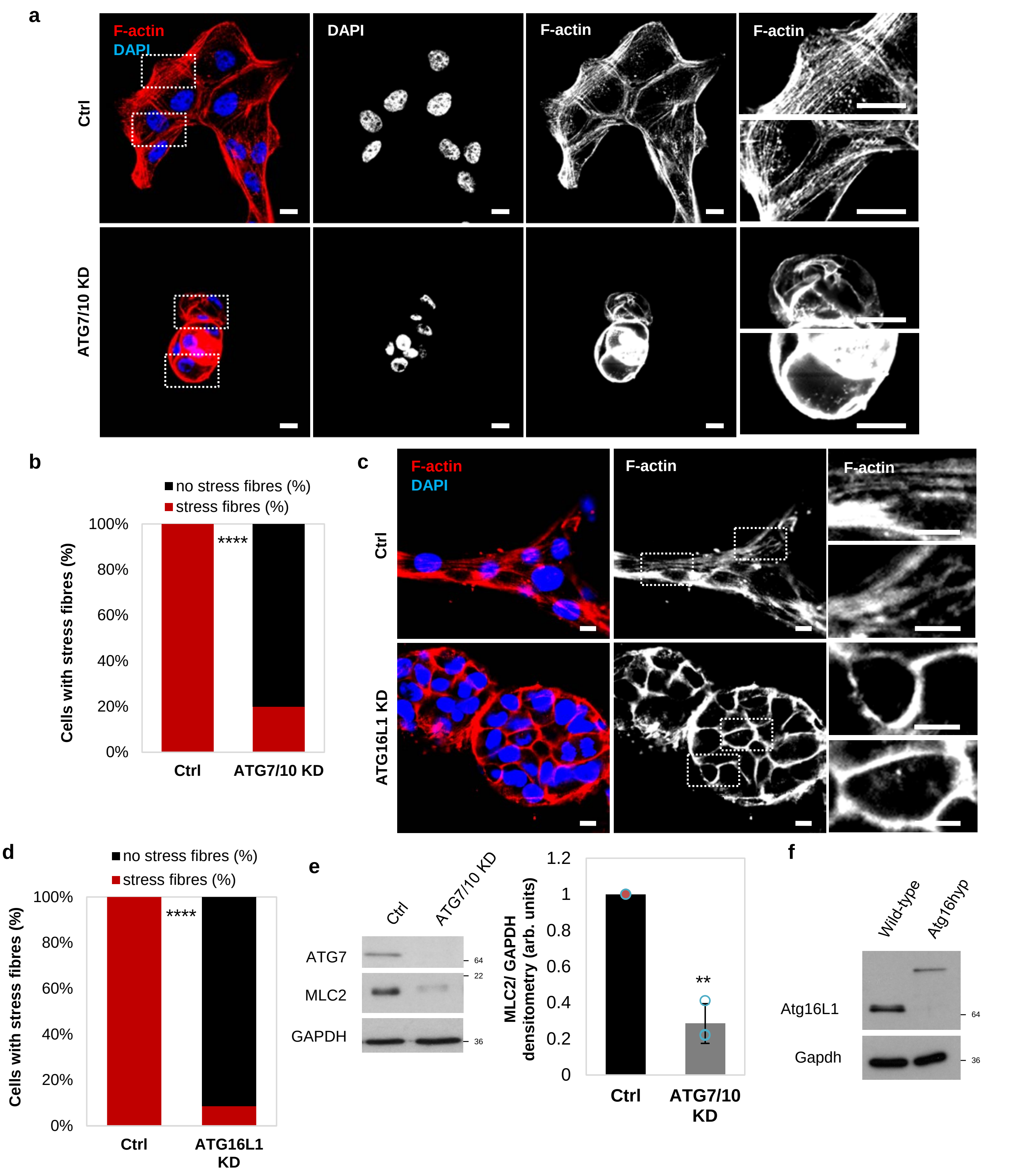

Supplementary Fig. 4 | Actin cytoskeleton is impaired upon ATG16L1 and ATG7/10 depletion in MCF10A cells.

a, Representative F-actin (phalloidin) staining in MCF10A cells exposed to control or ATG7/10 siRNAs. Scaler bars are 10 µm. The experiment was repeated 2 times with similar results.

b, Quantification of percentages of MCF10 cells with F-actin stress fibres. Cells were exposed to either control or ATG7/10 siRNAs (n = 100 cells per condition; ****P < 0.0001; chi-squared test). The experiment was repeated with similar results.

c, Representative F-actin (phalloidin) staining in MCF10A cells exposed to control or ATG716L1 siRNAs and grown on a 3D stiff extracellular matrix. Scale bars are 10 µm. The experiment was repeated 2 times with similar results.

d, Quantification of percentages of MCF10 cells with F-actin stress fibres. Cells were exposed to either control or ATG7/10 siRNAs (n = 100 cells per condition; ****P < 0.0001; chi-squared test). The experiment was repeated with similar results.

e, MLC2 and ATG7 protein levels in MCF10A cells exposed to either control or ATG7/10 siRNAs. GAPDH was used as loading control. Bars represent the mean ± s.d. (n = 3 independent experiments; **P < 0.01; two-tailed one sample t-test).

f, Representative Atg16L1 immunoblot in Atg16hyp pMECs from 3 independent experiments. Gapdh was used as loading control.

Exact *P* values for asterisks: **e** 0.0078.

Supplementary Fig. 5

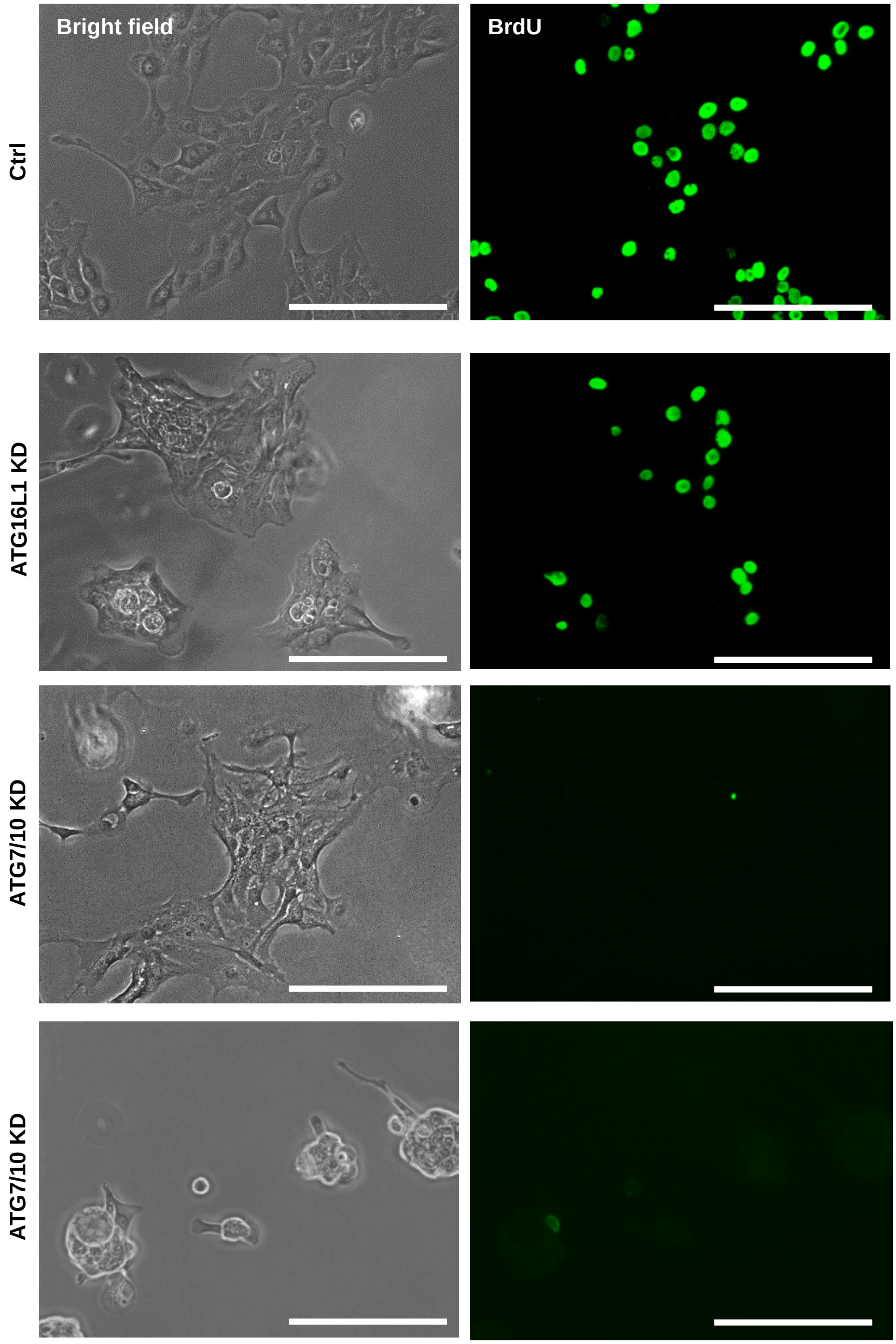

Supplementary Fig. 5 | Cell proliferation is reduced upon ATG16L1 or ATG7/10 depletion in MCF10A cells.

Representative bright field images and BrdU immunostaining in MCF10A cells exposed to control, ATG16L1 or ATG7/10 siRNAs. Scale bars are 100 µm. The experiment was repeated 2 times with similar results.

Supplementary Fig. 6

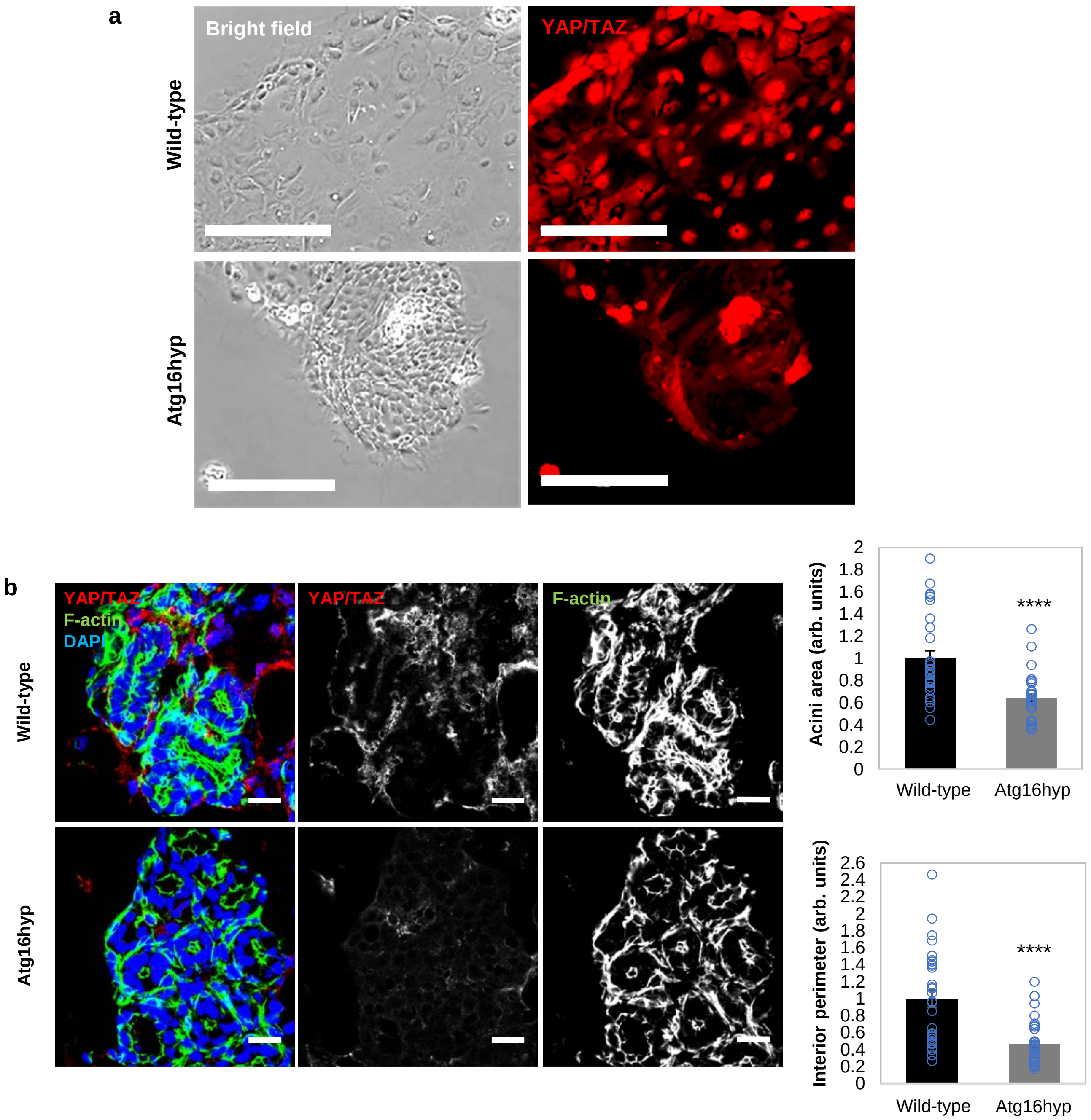

Supplementary Fig. 6 | YAP/TAZ nuclear localization and cell proliferation are reduced in Atg16hyp primary mammary epithelial cells.

a, Representative bright field images and endogenous YAP/TAZ immunostaining in primary mammary epithelial cells (pMECs) isolated from wild-type and Atg16L1 hypomorph (Atg16hyp) mice. Scale bars are 200 μm. The experiment was repeated at least 2 times with similar results.

b, Effect of decreased levels of Atg16L1 on mammary acini development. Mammary glands of wild-type or Atg16L1 hypomorph (Atg16hyp) female mice (at E16.5 gestation) were immunostained for YAP/TAZ and phalloidin. Scale bar, 20 μm. Bars represent the mean ± s.e.m. (n = 3 mice; ****$P < 0.0001$; two-tailed t-test).

Supplementary Fig. 7

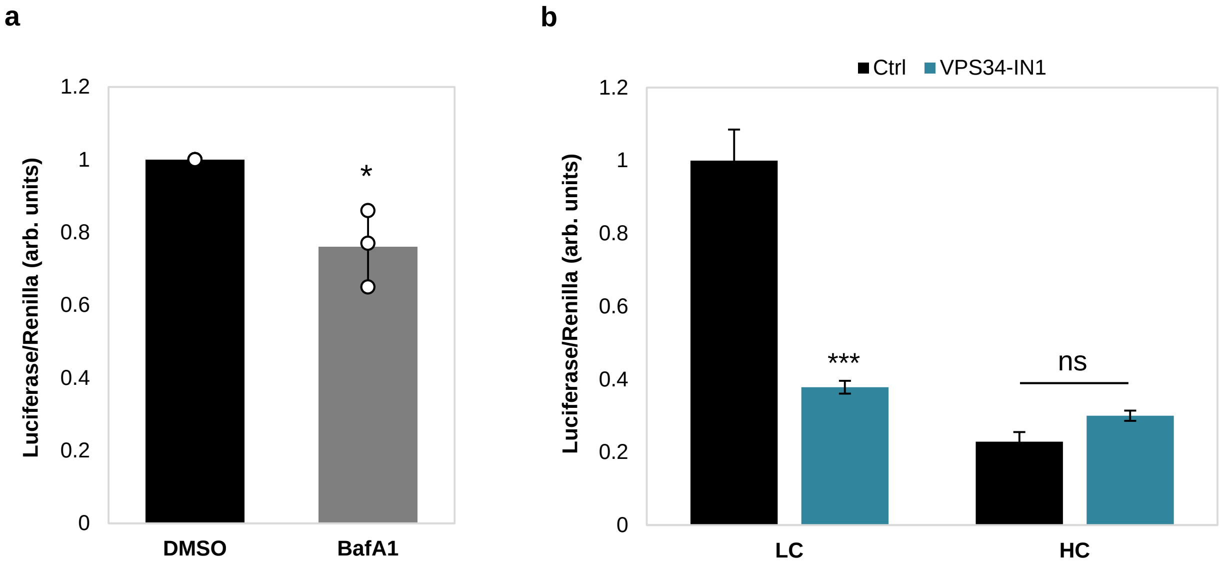

Supplementary Fig. 7 | YAP/TAZ activity is reduced upon chemical autophagy inhibition in MCF10A cells.

a, TEAD luciferase activity in MCF10A cells exposed to either control (DMSO) or BafA1 (400nM for 5 h). Bars represent the mean ± s.d. (n = 3 independent experiments; *$P < 0.05$; two-tailed one sample t-test).

b, TEAD luciferase activity in MCF10A cells exposed to either control (DMSO) or VPS34-IN1 (1 µM for 24 h). The high confluency condition was used as positive control for reduced TEAD luciferase activity. Bars represent the mean ± s.e.m. (n = 4 independent samples; ***$P < 0.001$, ns – not significant; two-tailed one sample t-test). The individual values are listed in Source data file.

Exact P values for asterisks: **a** 0.0169; and **b** 0.0004.

Supplementary Fig. 8

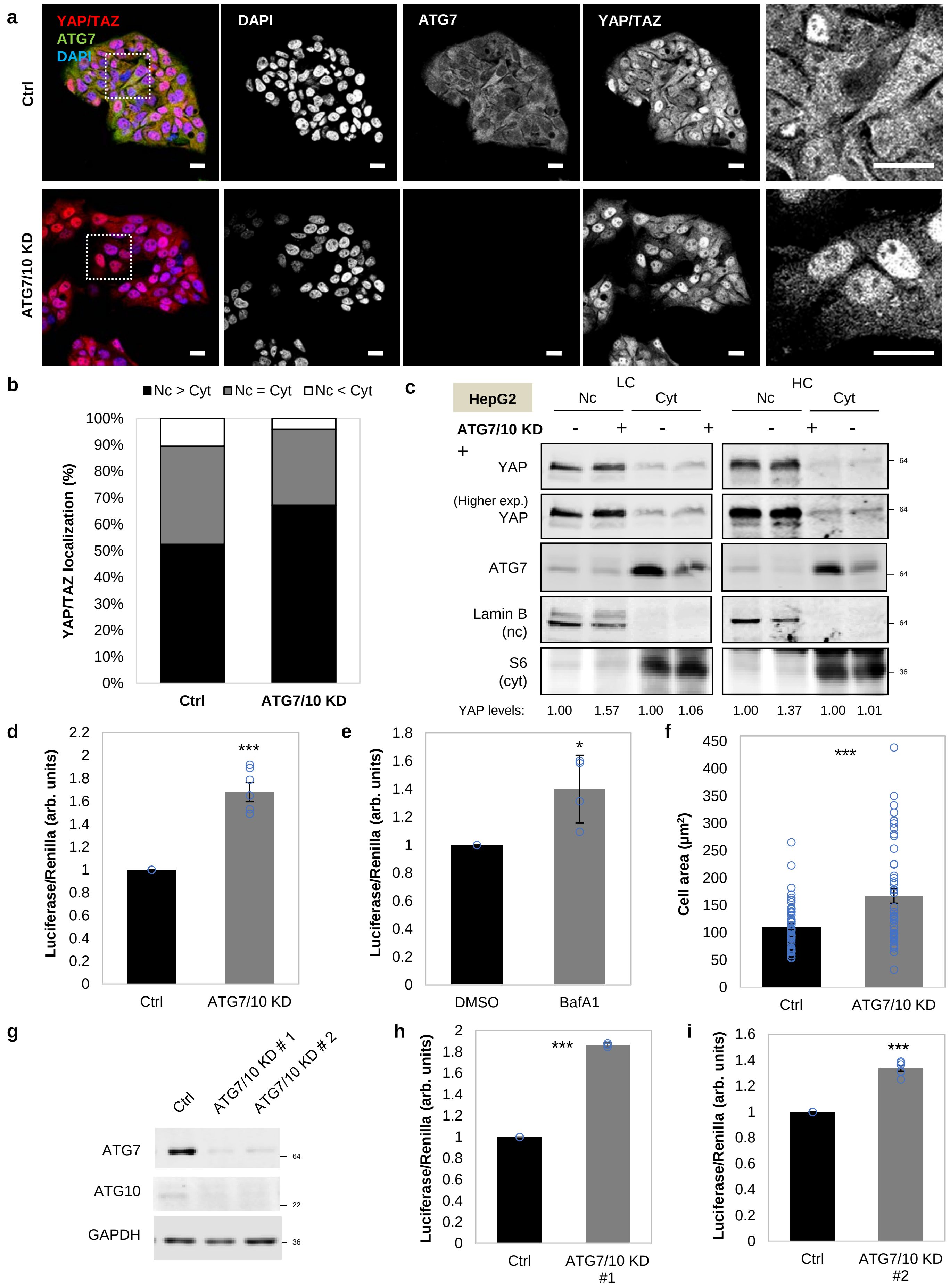

Supplementary Fig. 8 | YAP/TAZ is activated upon autophagy inhibition in HepG2 cells.
a, Representative confocal images of YAP/TAZ and ATG7 immunostaining in HepG2 cells exposed to either control or ATG7/10 siRNAs. Scale bars are 10 µM. The experiment was repeated 3 times with similar results.
b, YAP/TAZ localization in HepG2 cells treated as in **a** (*n* = 181 (Ctrl) and 122 (ATG7/10 KD) cells).
c, Representative immunoblots of YAP subcellular nuclear (Nc) and cytoplasmic (Cyt) fractions from control (scramble) and ATG7/10 knockdown HepG2 cells. Lamin B and S6 were used as loading controls for the nuclear and cytoplasmic fractions, respectively. The experiment was repeated 2 times with similar results.
d, TEAD luciferase activity of HepG2 cells exposed to ATG7/10 siRNAs. Bars represent the mean ± s.e.m. (*n* = 7 independent experiments; ****P* < 0.001; two-tailed one sample t-test).
e, TEAD luciferase activity of HepG2 cells exposed to BafA1 (400 nM, 6h). Bars represent the mean ± s.d. (*n* = 4 independent experiments; **P* < 0.05; two-tailed one sample t-test).
f, Size of HepG2 cells exposed to either control or ATG7/10 siRNAs. Confocal images of at least 50 cells per condition over 3 independent samples were analyzed to measure cell area using the ZEN software. Bars represent the mean ± s.e.m. (****P* < 0.001; two-tailed t-test).
g, Representative ATG7 and ATG10 immunoblots in HepG2 cells exposed to two different pairs of single ATG7 and ATG10 siRNA oligos. The experiment was repeated 2 times with similar results.
h, TEAD luciferase activity in HepG2 cells exposed to ATG7/10 siRNAs, pair # 1. Bars represent the mean ± s.d. (*n* = 3 independent samples; ****P* < 0.001; two-tailed one sample t-test).
i, TEAD luciferase activity in HepG2 cells exposed to ATG7/10 siRNAs, pair # 2. Bars represent the mean ± s.d. (*n* = 6 independent samples; ****P* < 0.001; two-tailed one sample t-test).
Exact *P* values for asterisks: **d** 0.0004; **e** 0.0461; **f** 0.0002; **h** 0.0001; and **i** 0.0005.

Supplementary Fig. 9

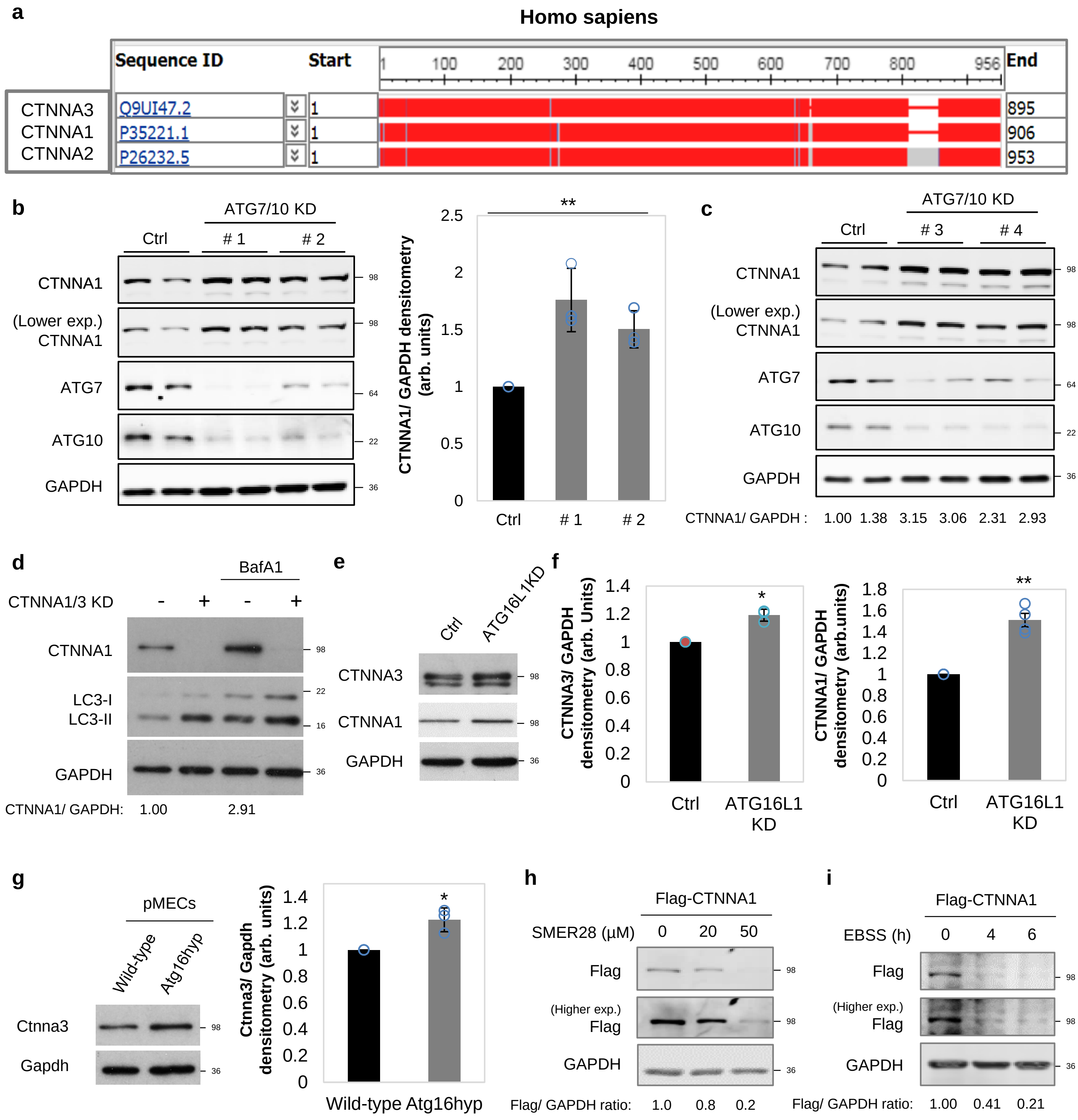

Supplementary Fig. 9 | α-catenins are degraded by autophagy.
a, Multiple protein sequence alignment for human α-catenins based on the conserved domain and local sequence similarity information, using the COBALT software.
CTNNA3: Q9UI47.2 [https://www.uniprot.org/uniprot/Q9UI47]
CTNNA1: P35221.1 [https://www.uniprot.org/uniprot/P35221]
CTNNA2: P26232.5 [https://www.uniprot.org/uniprot/P26232]

b, Representative CTNNA1 immunoblot in MCF10A cells exposed to two different pairs of single ATG7 and ATG10 siRNA oligos (# 1 and # 2). Bars represent the mean ± s.d. (n = 3 independent experiments; $^{**}P < 0.01$; two-way ANOVA).
c, Representative CTNNA1 immunoblot in MCF10A cells exposed to two different pairs of single ATG7 and ATG10 siRNA oligos (# 3 and # 4). The experiment was repeated twice with similar results.
d, Representative immunoblot of CTNNA1 knockdown efficiency in MCF10A cells exposed to either control or CTNNA1/3 siRNAs. Cells were treated with DMSO or BafA1 (200nM) for the last 12 h. The experiment was repeated twice with similar results.
e, Representative α-catenins immunoblots in ATG16L1 knockdown MCF10A cells. GAPDH was used as loading control. The experiment was repeated twice with similar results.
f, Densitometry of α-catenins in ATG16L1 knockdown MCF10A cells. Bars represent the mean ± s.e.m. (n = 4 independent experiments; $^{**}P < 0.01$, $^{*}P < 0.05$; two-tailed one sample t-test).
g, Representative Ctnna3 immunoblot in Atg16hyp pMECs. Bars represent the mean ± s.d. (n = 3 independent experiments; $^{*}P < 0.05$; two-tailed one sample t-test).
h, Representative immunoblot of Flag-CTNNA1 levels in MCF10A cells exposed to different concentrations of SMER 28 (0, 20 and 50 μM) for 24 h. The blot is the same as in Fig. 7d, and we are showing only the first 3 lanes for clarity.
i, Representative immunoblot of Flag-CTNNA1 levels in MCF10A cells cultured in EBSS for different time points (0, 4 and 6 h).
Exact P values for asterisks: **b** 0.0066; **f** (from left to right) 0.0159, 0.0040; and **g** 0.0480.

Supplementary Fig. 10

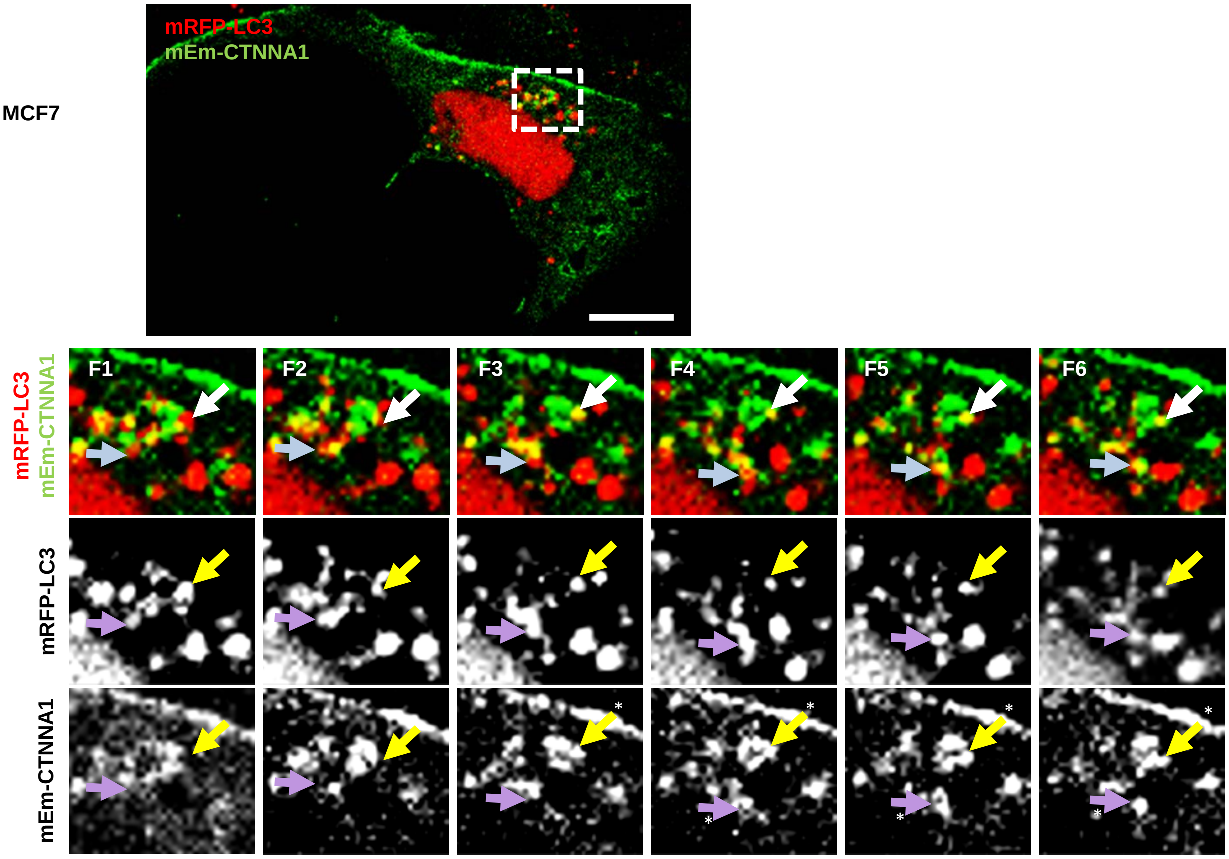

Supplementary Fig. 10 | mEm-CTNNA1 accumulates in autophagosomes in MCF7 cells. Confocal live imaging of MCF7 cells expressing mEm-CTNNA1 and the autophagosome marker mRFP-LC3. The arrows show the autophagosomes (mRFP-LC3 positive vesicles) enriching in mEm-CTNNA1. Scale bars are 10 µm. The experiment was repeated twice with similar results.

Supplementary Fig. 11

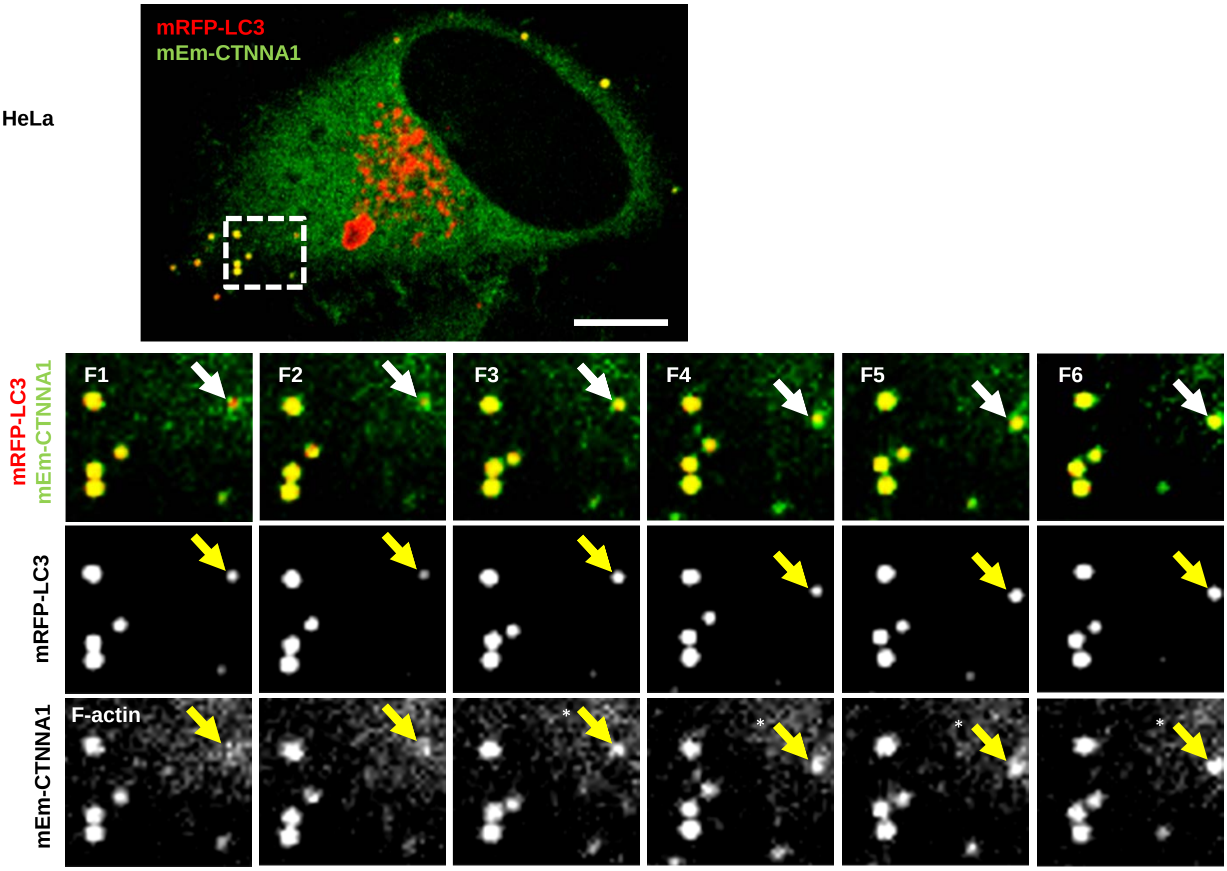

Supplementary Fig. 11 | mEm-CTNNA1 accumulates in autophagosomes in HeLa cells. Confocal live imaging of HeLa cells expressing mEm-CTNNA1 and the autophagosome marker mRFP-LC3. The arrows show the autophagosomes (mRFP-LC3 positive vesicles) enriched in mEm-CTNNA1. Scale bars are 10 µm. The experiment was repeated twice with similar results.

Supplementary Fig. 12

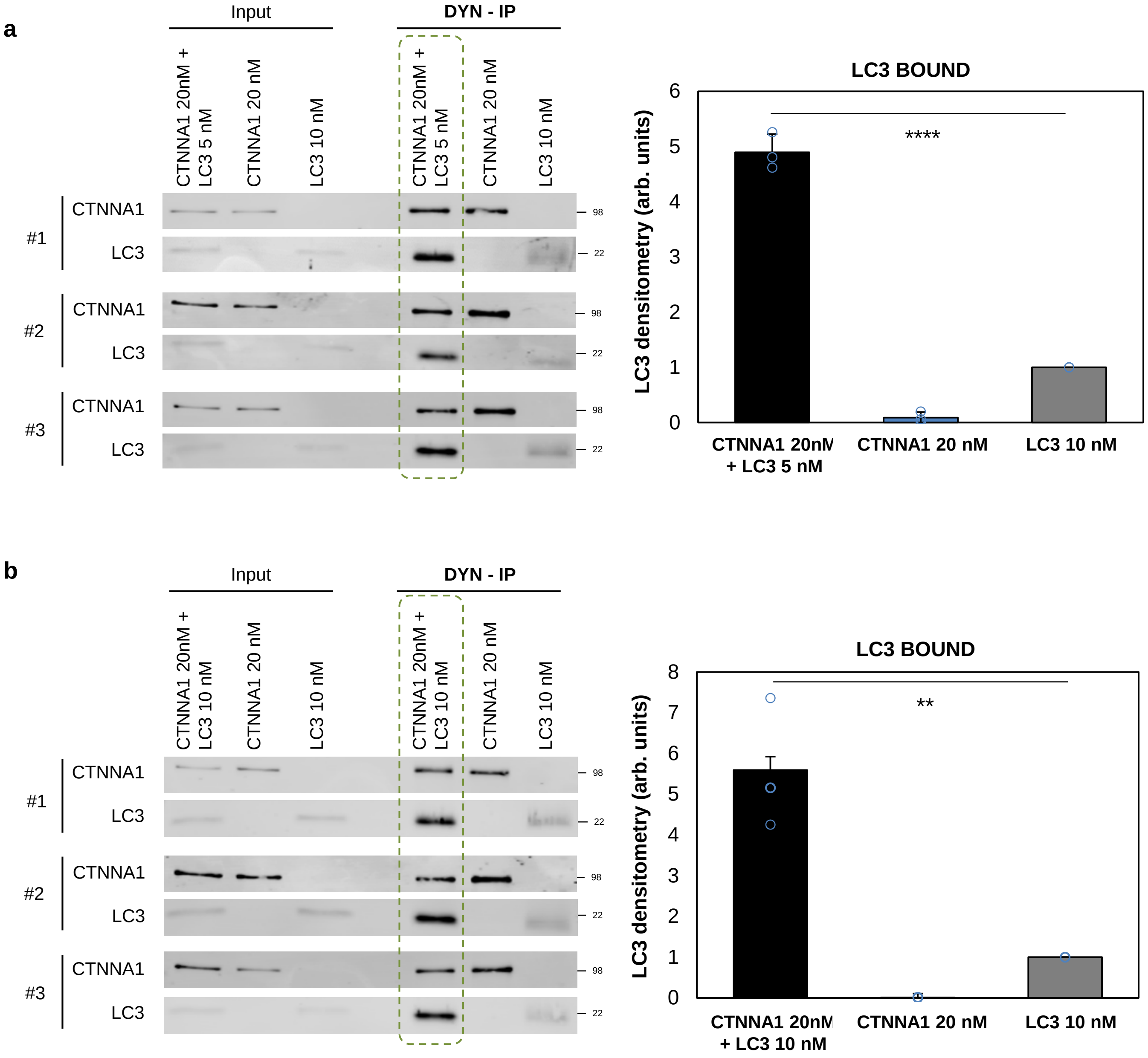

Supplementary Fig.12 | In-vitro binding of purified proteins: CTNNA1 and LC3.
a, Representative immunoblot of *in-vitro* binding of purified proteins: 20 nM CTNNA1 and 5 nM LC3. Bars represent the mean ± s.d. (n = 3 independent experiments; ****P < 0.0001; two-tailed one sample t-test).
b, Representative immunoblot of *in-vitro* binding of purified proteins: 20 nM CTNNA1 and 10 nM LC3. Bars represent the mean ± s.d. (n = 3 independent experiments; **P < 0.01; two-tailed one sample t-test).
Exact *P* values for asterisks: **b** 0.0076.

Supplementary Fig. 13

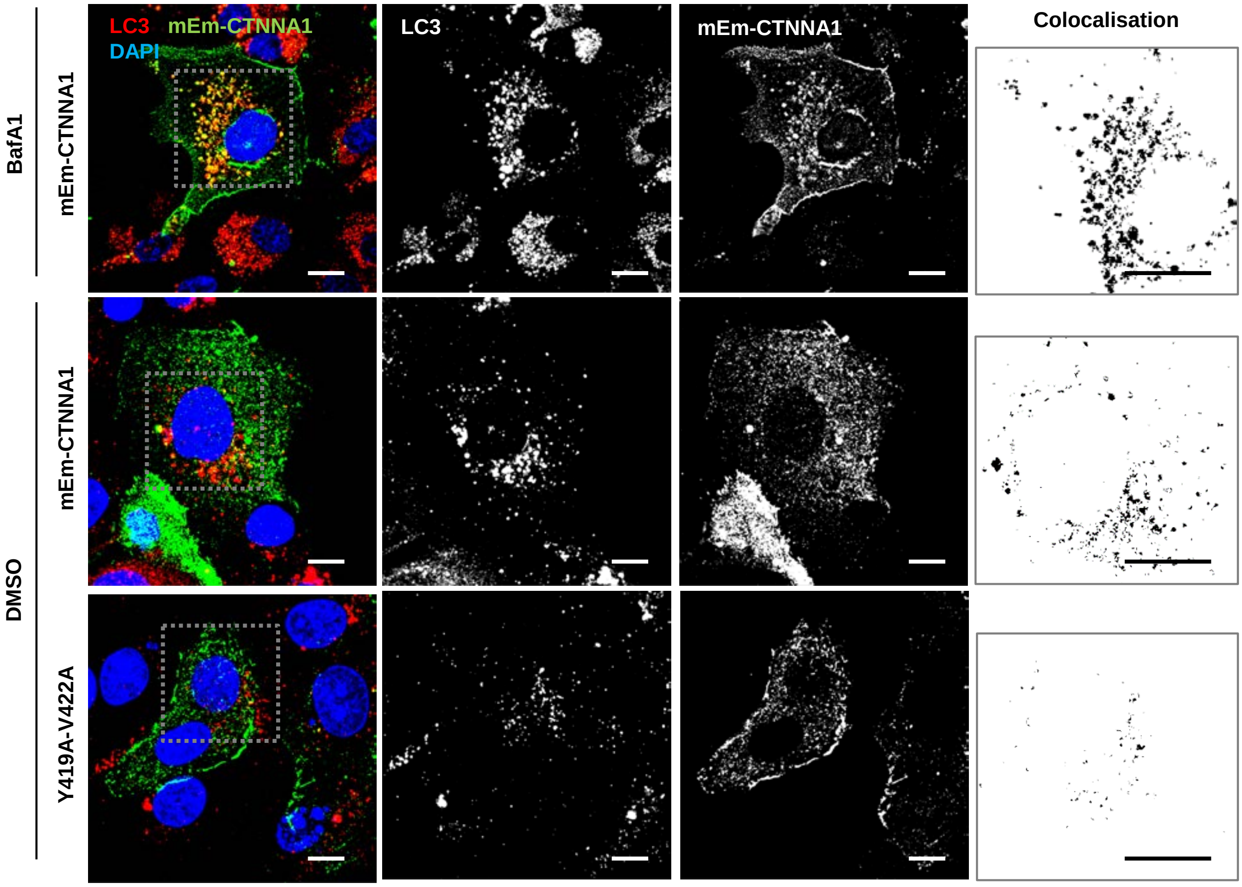

Supplementary Fig. 13 | α-catenins colocalise with autophagosome marker, LC3. Representative confocal images of mEm-CTNNA1 – endogenous LC3 colocalization in MCF10A cells transiently transfected with either wild-type or Y419A-V422A mEmCTNNA1. The mEm-CTNNA1 – endogenous LC3 colocalization is depicted on the right. Scale bars are 10 µm. The experiment was repeated twice with similar results.

Supplementary Fig. 14

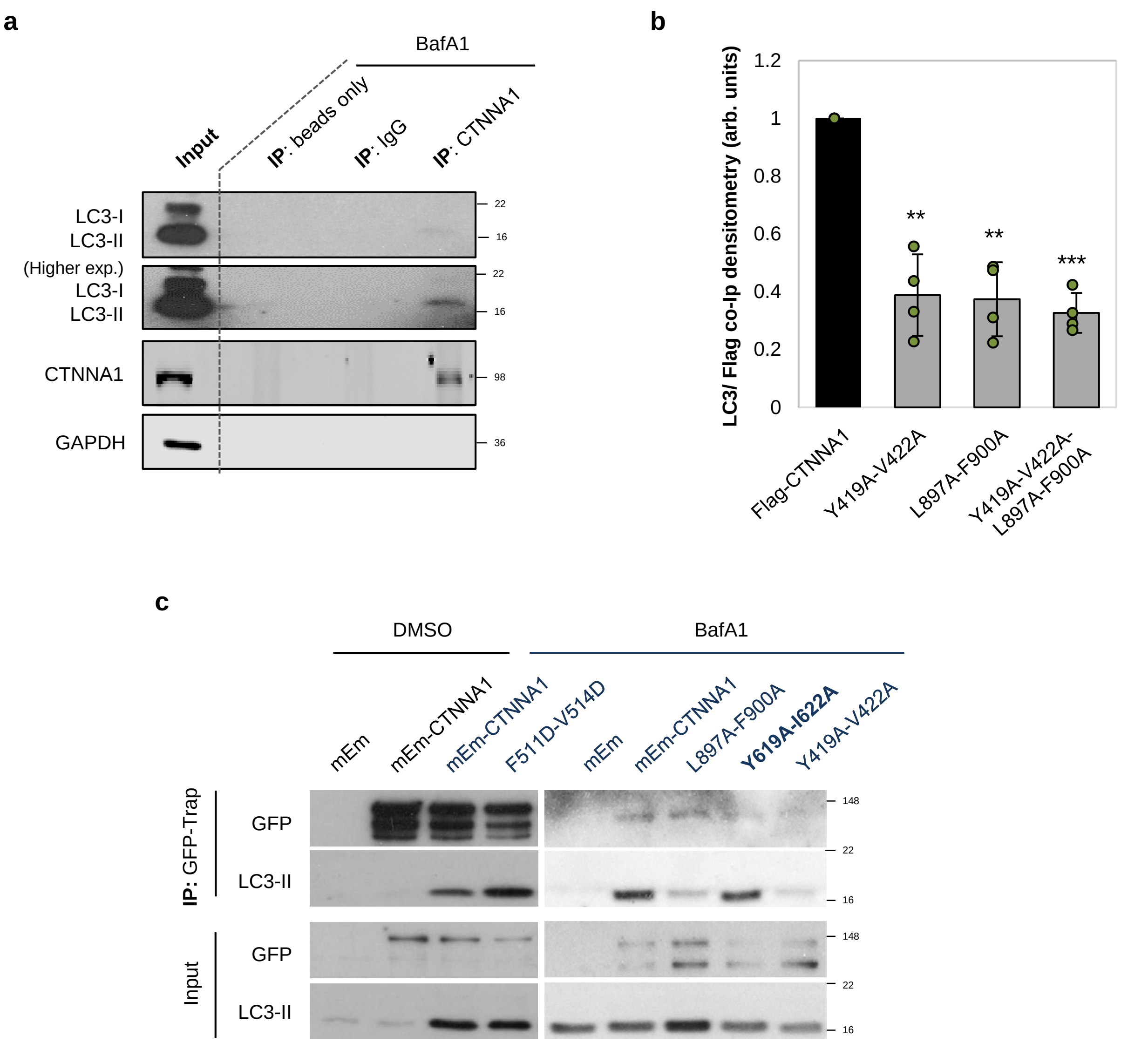

Supplementary Fig. 14 | Co-immunoprecipitation of endogenous LC3 with CTNNA1.

a, Co-immunoprecipitation of endogenous LC3 and CTNNA1 in BafA1 treated MCF10A cells. The experiment was repeated 3 times with similar results.

b, Quantification of co-immunoprecipitated endogenous LC3 with Flag-CTNNA1 (WT or mutants). Bars represent the mean ± s.d. (*n* = 4 independent experiments; ****P* < 0.001, ***P* < 0.01; two-tailed one sample t-test).

c, Co-immunoprecipitation of endogenous LC3 with transiently transfected wild-type mEm-CTNNA1 or the indicated mEm-CTNNA1 mutants in MCF10A cells. The mEm-tagged proteins were pulled down using the GFP-Trap technology. The experiment was repeated 3 times with similar results.

Exact *P* values for asterisks (from left to right): **a** 0.0032, 0.0023, 0.0003.

Supplementary Fig. 15

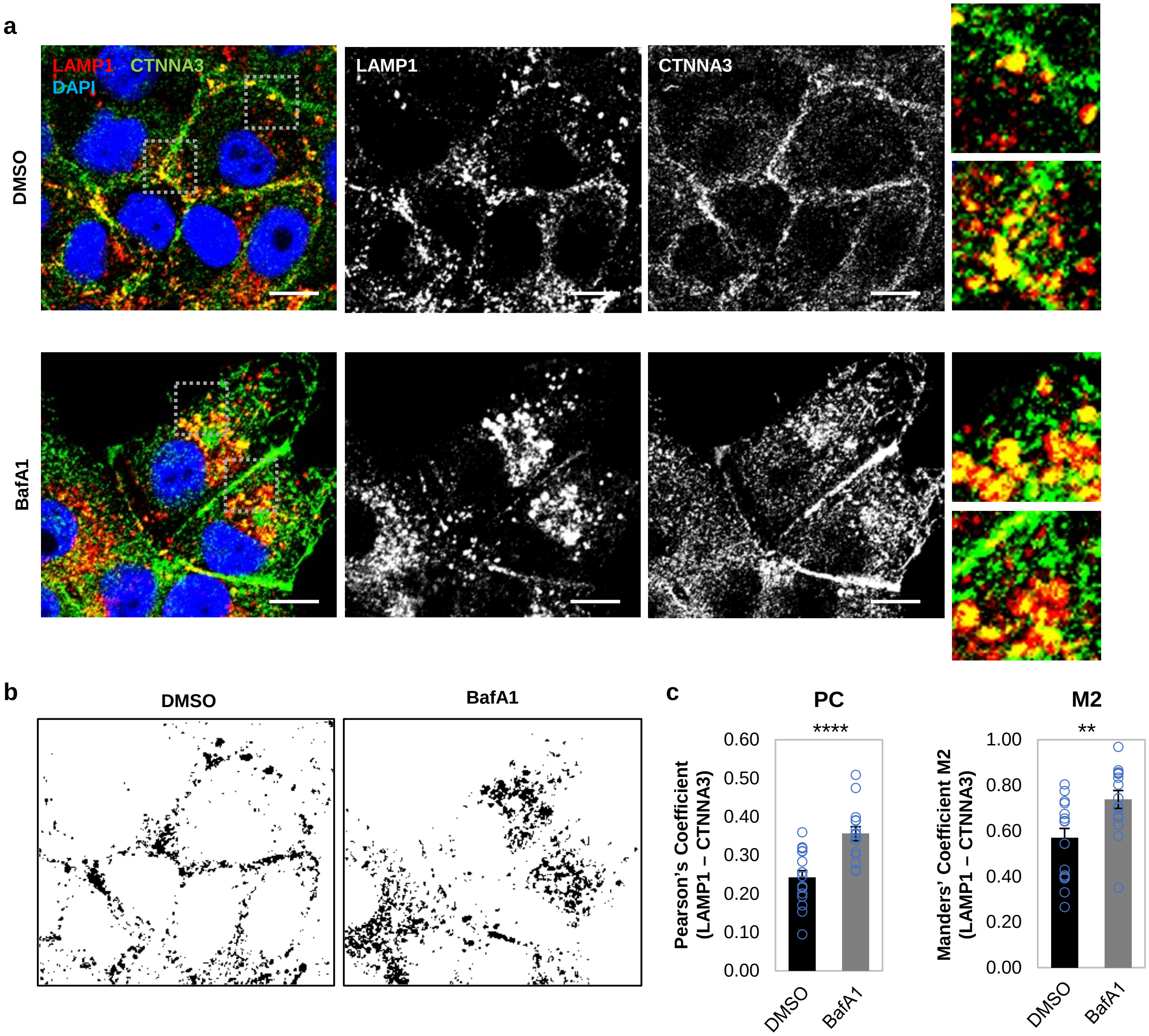

Supplementary Fig.15 | α-catenins colocalise with the lysosomal marker, LAMP1.
a, Representative confocal images of endogenous CTNNA3 – LC3 colocalization in MCF10A cells. Cells were exposed to either DMSO or BafA1 (400 nM) for 4 h. Scale bars are 10 μm. The experiment was repeated twice with similar results.
b, Colocalisation points for the images presented at (**a**).
c, Quantification of colocalization for the experiment in (**a**). The Pearson's and Mander's (fraction of CTNNA3 overlapping LAMP1) coefficients were quantified for more than 15 microscopy fields of at least 5 cells (each field) over n = 3 independent experiments. Bars represent the mean ± s.e.m. (****P < 0.0001, **P < 0.01; two-tailed t-test). The data points show the means of individuals cell fields.
Exact P values for asterisks: **c** 0.0031.

Supplementary Fig. 16

a

```
CTNNA3      120 AVVQAARALLAAVTRLLILADMIDVMCLLQHVSAFQRTFESLKNVANKSDLQKTYQKLGK        179
CTNNA1      123 NMVRAARALLSAVTRLLILADMADVYKLLVQLKVVEDGILKLRNAGNEQDLGIQYKALKP        182
CTNNA2      122 TMVRAARALLSAVTRLLILADMADVMRLLSHLKIVEEALEAVKNATNEQDLANRFKEFGK        181
                :*:******:*********** **   ** ::. .:  :  ::*. *:.**   :: :

CTNNA3      228 HSDVASLKASKDTVCEEIQNALNVISNASQGIQNMTTP--PEPQAATLGSALDELENLIV        285
CTNNA1      231 HPDVAAYKANRDLIYKQLQQAVTGISNAAQATASDDASQHQGGGGGELAYALNNFDKQII        290
CTNNA2      230 HPDVAATRANRDYVFKQVQEAIAGISNAAQATSPTDEAKG-HTGIGELAAALNEFDNKII        288
                * ***: :*.:* : :::*:*:  ****:*.              . *. **::::: *:

CTNNA3      382 IDHVSDSFLDTTVPLLVLIEAAKNGREKEIKEYAAIFHEHTSRLVEVANLACSMSTNEDG        441
CTNNA1      387 MDHVSDSFLETNVPLLVLIEAAKNGNEKEVKEYAQVFREHANKLIEVANLACSISNNEEG        446
CTNNA2      385 MDHISDSFLETNVPLLVLIEAAKSGNEKEVKEYAQVFREHANKLVEVANLACSISNNEEG        444
                :**:*****:*.***********.*.***:****  :*:**:.:*:********:*.**:*

CTNNA3      476 KNTMEMYKRTWENHIHVLTEAVDDITSIDDFLAVSESHILEDVNKCIIALRDQDADNLDR        535
CTNNA1      481 QENMDLFKEQWEKQVRVLTDAVDDITSIDDFLAVSENHILEDVNKCVIALQEKDVDGLDR        540
CTNNA2      479 QDNMDVFKDQWEKQVRVLTEAVDDITSVDDFLSVSENHILEDVNKCVIALQEGDVDTLDR        538
                ::.*:::*  **::::***:*******:****:***.*********:***::  *.* ***

CTNNA3      596 SSLNVLDDNQFVDISKKIYDTIHDIRCSVMMIRTPEELEDVSDLEEE-HEVRSHTSIQTE        654
CTNNA1      601 DPAQPMDENEFIDASRLVYDGIRDIRKAVLMIRTPEELD-DSDFETEDFDVRSRTSVQTE        659
CTNNA2      599 NVPQPFEENEFIDASRLVYDGVRDIRKAVLMIRTPEELEDDSDFEQEDYDVRSRTSVQTE        658
                .  : :::*:*:* *: :** ::*** :*:********:   **:* *  .:***:**:***

CTNNA3      841 PRHPVVMWRMKAPAKKPLIKREKPEETCAAVRRGSAKKKIHPLQVMSEFRGRQIY        895
CTNNA1      852 LNLPAVSWKMKAPEKKPLVKREKQDETQTKIKRASQKKHVNPVQALSEFKAMDSI        906
CTNNA2      899 VNSPVVSWKMKAPEKKPLVKREKPEEFQTRVRRGSQKKHISPVQALSEFKAMDSF        953
                 . *.* *:**** ****:**** :*   : ::*.* **:: *:*.:***:.  :
```

Mutations:

Y419A-V422A

F511D-V514D

Y619A-I622A

L897A-F900A

b

Protein Blast: CTNNA1 (*H. sapiens*) and Ctnna1 (*M. musculus*)

Score	Expect	Method	Identities	Positives	Gaps
1848 bits(4786)	0.0	Compositional matrix adjust.	901/906(99%)	904/906(99%)	0/906(0%)

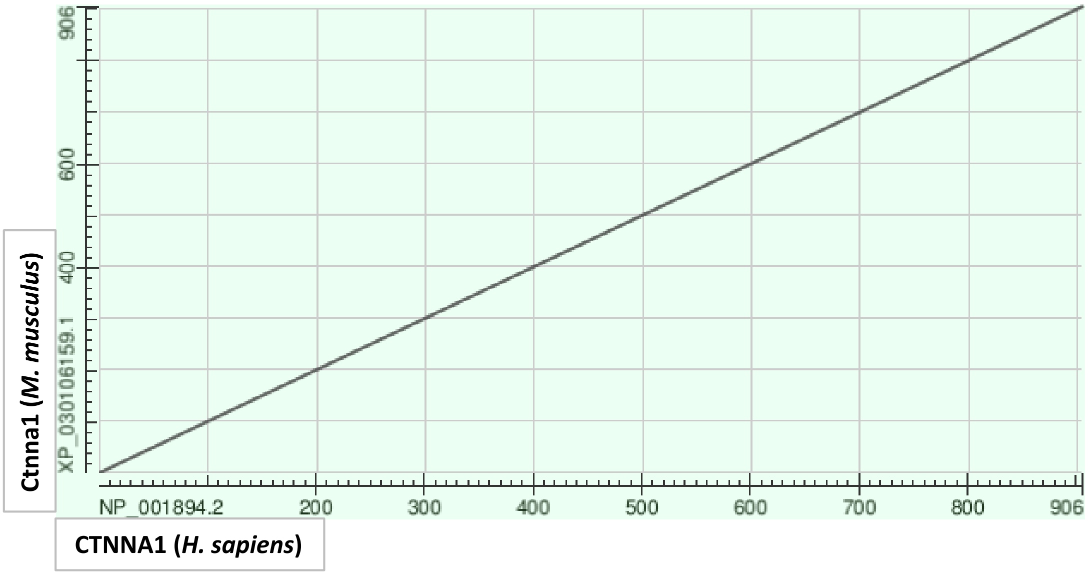

Supplementary Fig. 16 | Amino-acid sequences of human α-catenins.

a, Potential LIR motifs identification and BLAST amino acid sequences alignment of human α-catenins. The studied CTNNA1 mutants are depicted on the right.

b, Protein Blast analysis of CTNNA1 (*H. sapiens*) and Ctnna1 (*M. musculus*) showing 99 % identity.

Supplementary Fig. 17

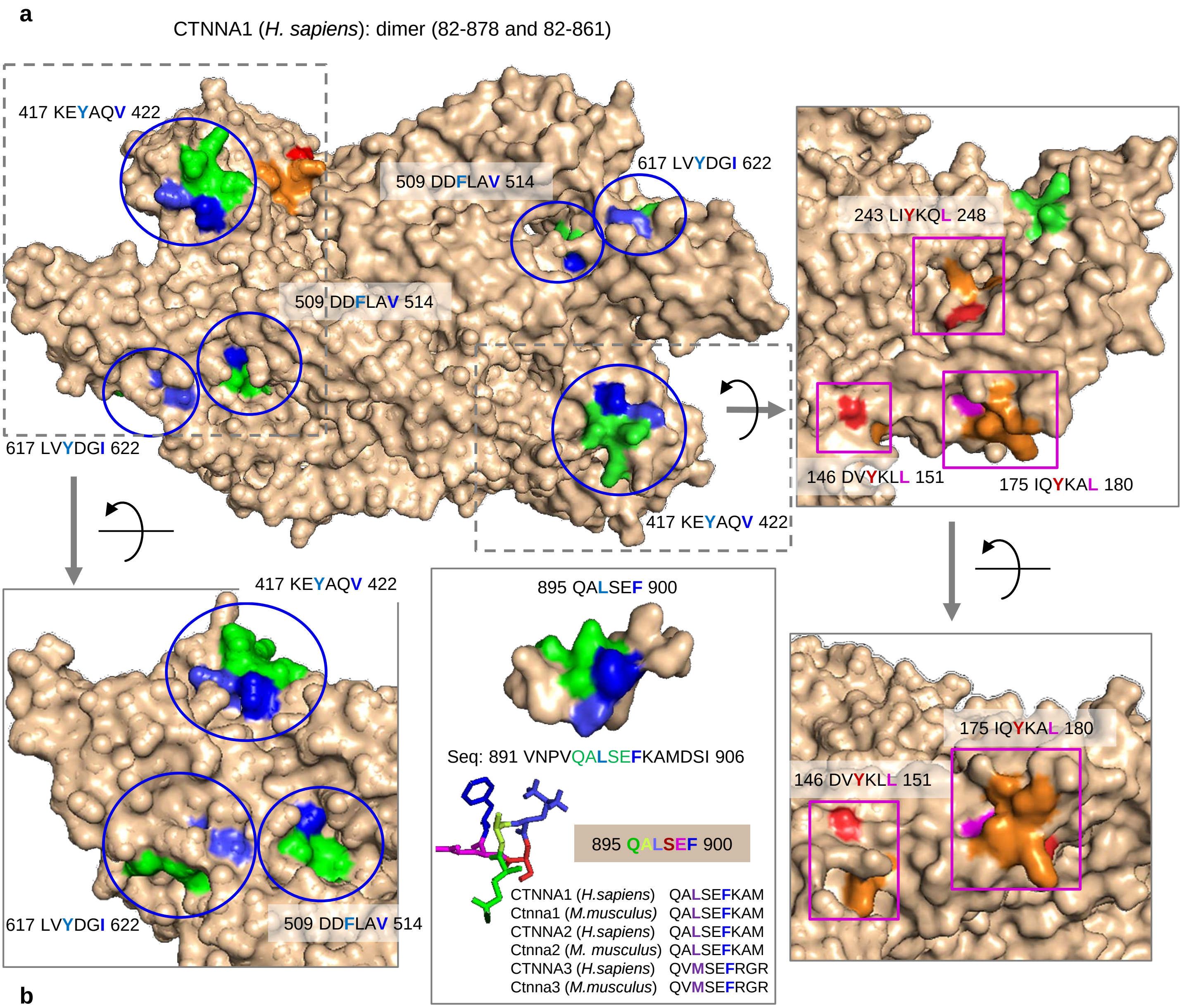

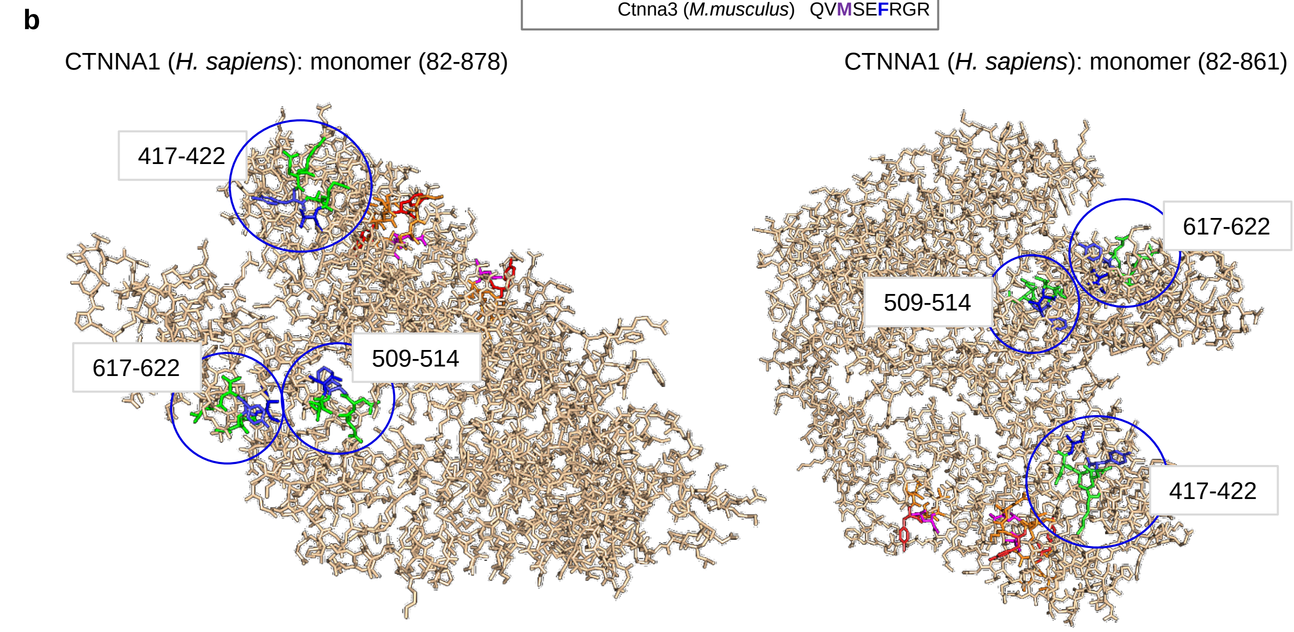

Supplementary Fig. 17 | Structural analysis of potential LIR regions in human CTNNA1.

a, CTNNA1 (*H. sapiens*) as dimer (82-878 and 82-861) (pdb. 4IGG [http://doi.org/10.2210/pdb4IGG/pdb]) showing the location of studied (mutated) LIR regions colored in blue-green. The potential LIR regions identified based on their amino acid sequences and coloured in orange-purple are facing the interior of the CTNNA1 molecule (unlikely to interact with LC3). Only two blue-green regions (possibly responsible for α-catenin – LC3 interaction) are composed of amino acids found on the surface of the CTNNA1 molecule facing outwards: 417-422 (KEYAQV) and 895-900 (QALSEF). As the 891-906 region is not included in the pdb. 4IGG [http://doi.org/10.2210/pdb4IGG/pdb] , this amino acid sequence was computed using Phyre2.

b, CTNNA1 (*H. sapiens*) as monomers 82-878 and 82-861, respectively (pdb. 4IGG [http://doi.org/10.2210/pdb4IGG/pdb]) showing the location of mutations, colored in blue-green.

Supplementary Fig. 18

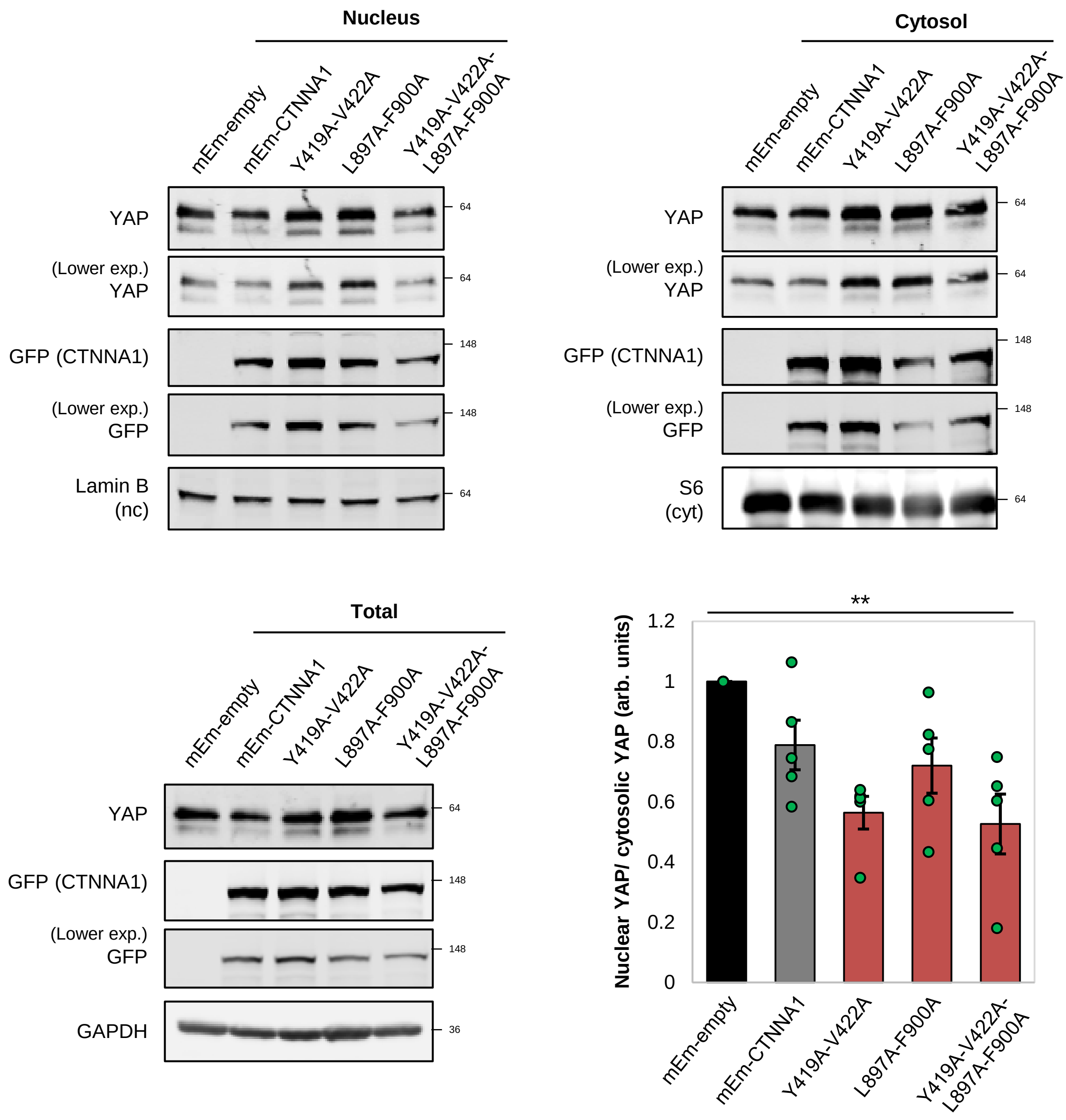

Supplementary Fig. 18 | YAP/TAZ nuclear to cytoplasmic translocation upon mEm-CTNNA1 (WT or LIR-defective mutants) overexpression in MCF10A cells.
Representative immunoblot of YAP subcellular nuclear (Nc) and cytoplasmic (Cyt) fractions from MCF10A cells transiently expressing the wild type, L897A-F900A, Y419A-V422A or Y419A-V422A-L897A-F900A mEm-CTNNA1 constructs. Lamin B, S6 and GAPDH were used as loading controls for the nuclear and cytoplasmic fractions or total levels. Bars represent the mean ± s.e.m. (n = 5 independent experiments; $^{**}P < 0.01$; one-way ANOVA test).
Exact P values for asterisks: 0.0038.

Supplementary Fig. 19

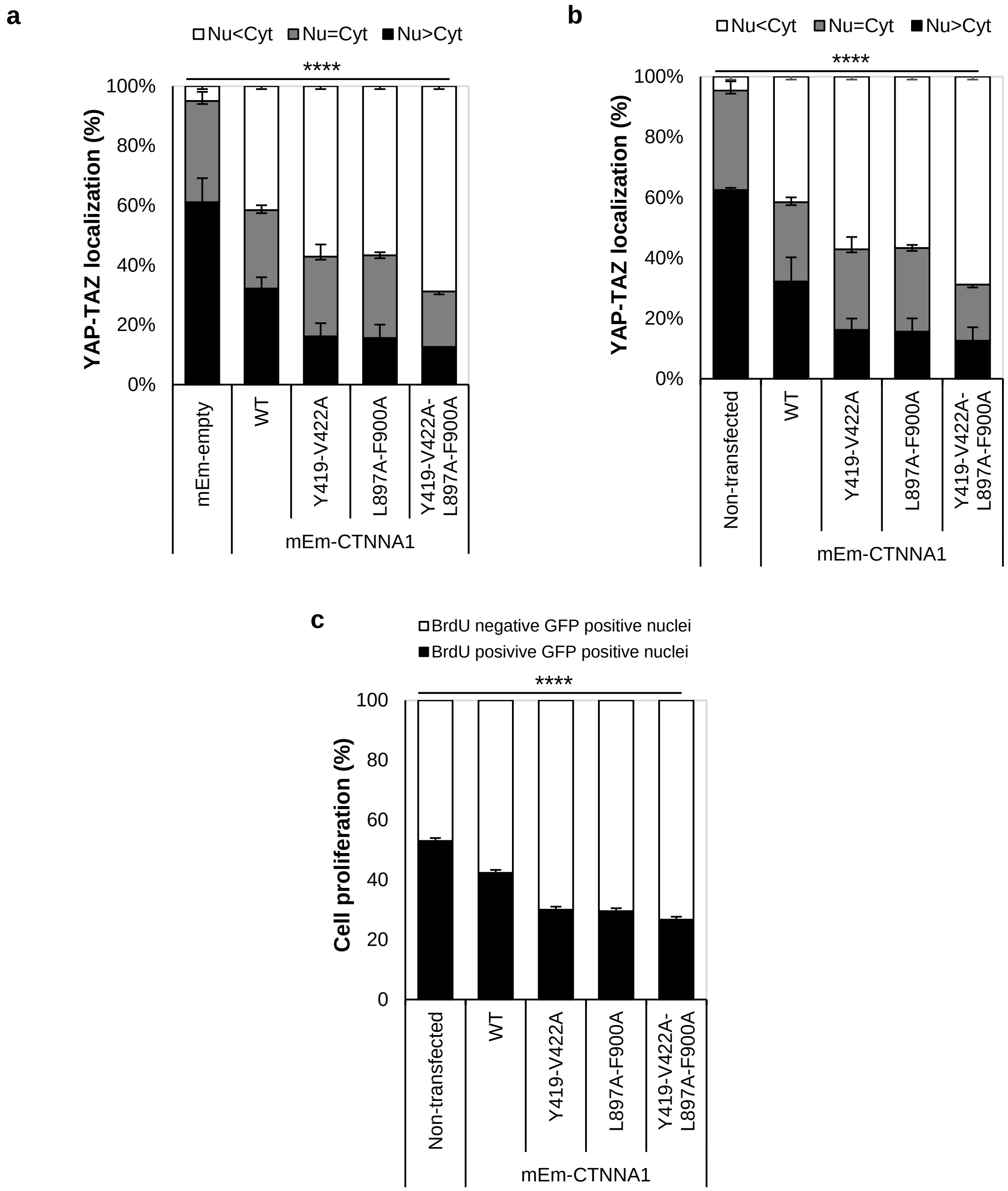

Supplementary Fig. 19 | YAP/TAZ activity is reduced upon mEm-CTNNA1 (WT or LIR-defective mutants) overexpression in MCF10A cells.

a, YAP/TAZ localization in MCF10A cells transfected with mEm-empty, wild-type or mEm-CTNNA1 mutants. Bars represent the mean ± s.e.m. (*n* = 4 independent experiments; *****P* < 0.0001; two-way ANOVA).

b, YAP/TAZ localization in MCF10A cells: non-transfected or transfected with the wild-type or mEm-CTNNA1 mutants. Bars represent the mean ± s.e.m. (*n* = 4 independent experiments; ****P* < 0.0001; two-way ANOVA).

c, Percentages of BrdU-positive cells in MCF10A cells: non-transfected or transfected with the wild-type or mutant forms of mEm-CTNNA1. Bars represent the mean ± s.e.m. (*n* = 5 independent experiments; *****P* < 0.0001; two-way ANOVA).

Supplementary Fig. 20

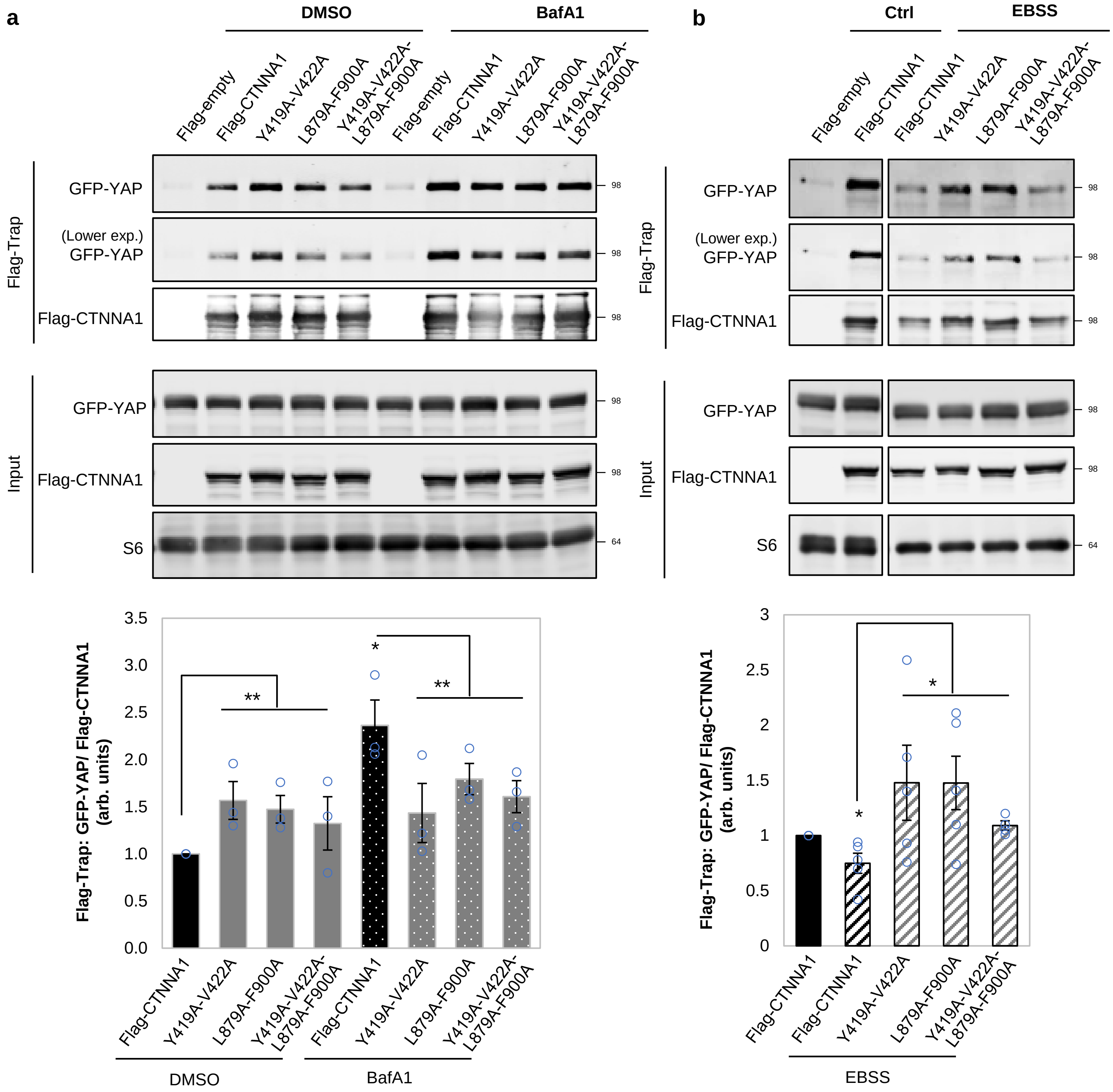

Supplementary Fig. 20 | Co-immunoprecipitation of GFP-YAP with Flag-CTNNA1 LIR-defective mutants is unaffected by BafA1 treatment.

a, Representative co-IP immunoblot of GFP-YAP with wild-type or Flag-CTNNA1 mutants (L897A-F900A, Y419A-V422A or Y419A-V422A-L897A-F900A) in HEK293T cells, in the presence or absence of BafA1 (200 nM, 16 h). S6 was used as loading control for the input cytosolic fractions. The Flag-tagged proteins were pulled down using the Flag-Trap technology. Bars represent the mean ± s.e.m. (n = 3 independent experiments; **P < 0.01, *P < 0.05; one-way ANOVA test). Exact P values for asterisks (from left to right): 0.0020, 0.0162, 0.0085.

b, Representative co-IP immunoblot of GFP-YAP with wild-type or Flag-CTNNA1 mutants in HEK293T cells exposed to EBSS (6 h). S6 was used as loading control for the input cytosolic fractions. The Flag-tagged proteins were pulled down by Flag-Trap technology. Bars represent the mean ± s.e.m. (n = 5 independent experiments; *P < 0.05 (0.0302, 0.0274); one-way ANOVA test).

Supplementary Fig. 21

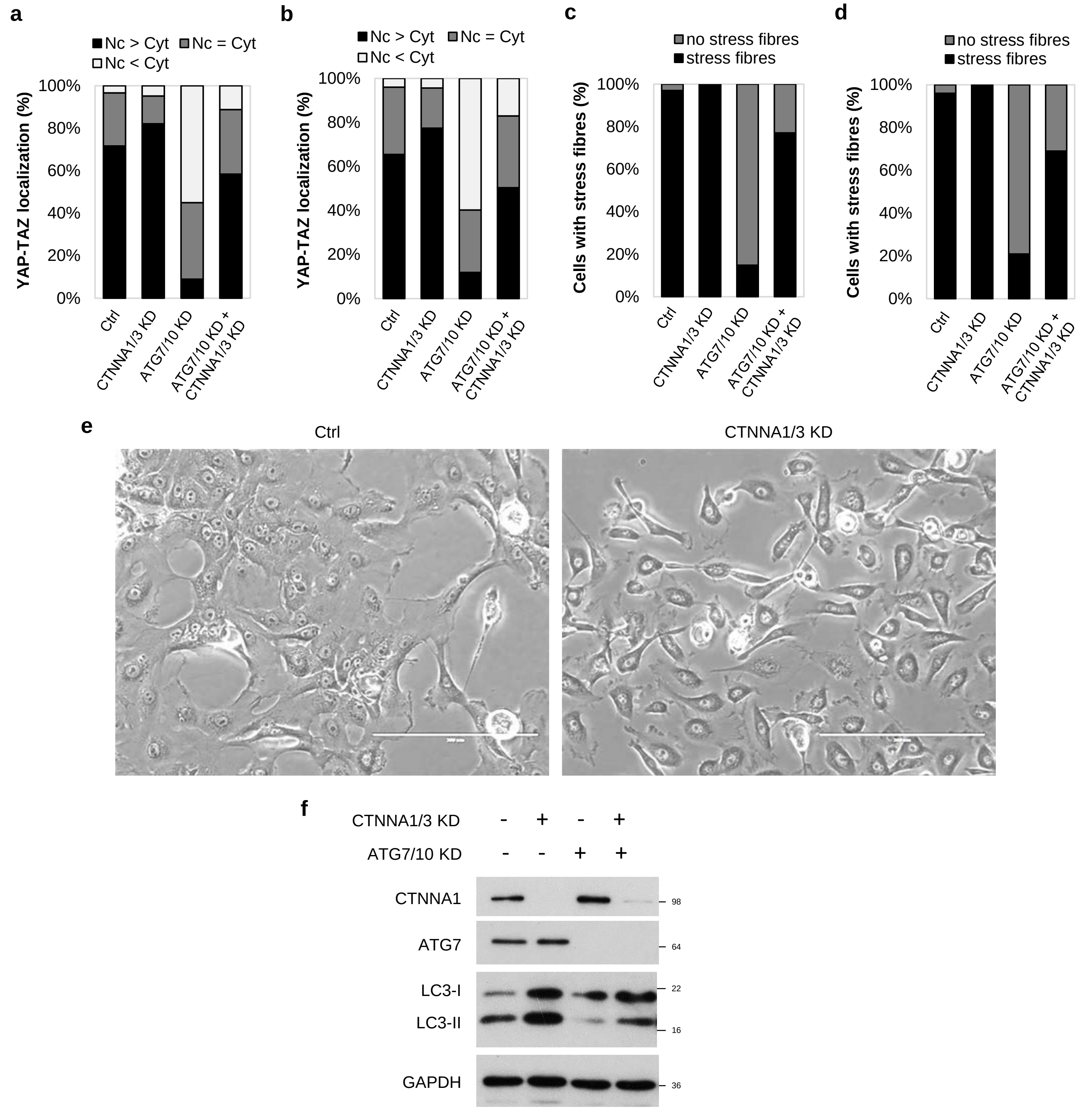

Supplementary Fig. 21 | Knockdown efficiency in MCF10A cells.
a, The experimental repeat for Fig. 9b – *n* (from left to right) = 268/ 229/ 200/ 178 cells.
b, Pooled data from the 2 independent experiments presented in Fig. 9b and Supplementary Fig. 21a. Bars represent the mean.
c, The experimental repeat for Fig. 9c – *n* (from left to right) = 268/ 229/ 200/ 178 cells.
d, Pooled data from the 2 independent experiments presented in Fig. 9c and Supplementary Fig. 21c. Bars represent the mean.

e, Representative bright field images of MCF10A cells exposed to either control or CTNNA1/3 siRNAs. Scale bars are 10 µm. The experiment was repeated twice with similar results.
f, Representative immunoblot of CTNNA1 and ATG7 protein levels in MCF10A cells exposed to ATG7/10 and CTNNA1/3 siRNAs. The experiment was repeated twice with similar results.

Supplementary Fig. 22

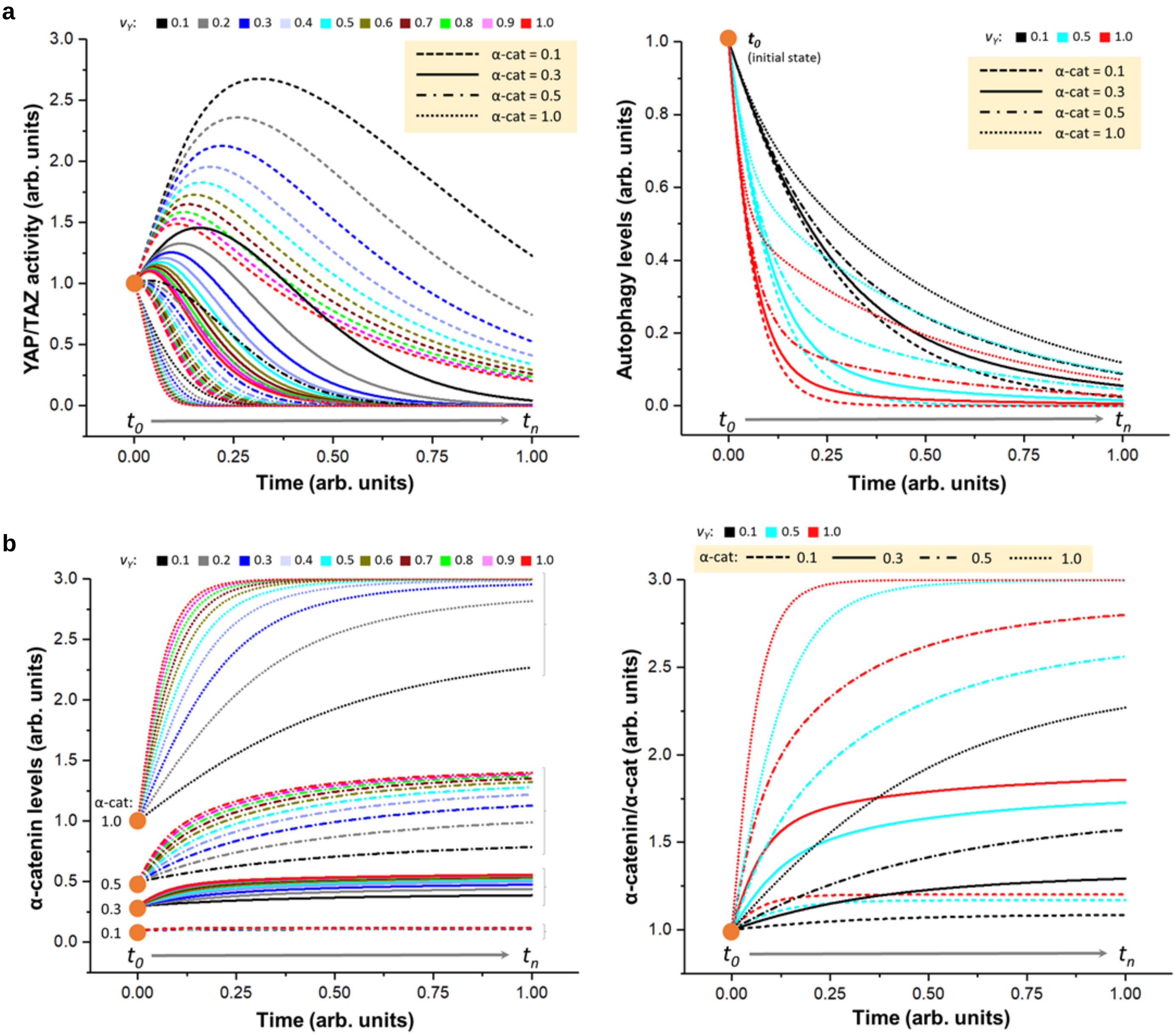

Supplementary Fig. 22 | Predicted time variation of mathematical model parameters upon autophagy inhibition.

a, Time variation of the indicated parameters: YAP/TAZ activity (left) and autophagy levels (right).

b, Time variation of α-catenin levels. The graph on the right shows the normalized α-catenin levels to initial α-cat values. Conditions with low initial α-cat values show reduced α-catenin accumulation upon autophagy inhibition (towards t_n).

Supplementary Fig. 23

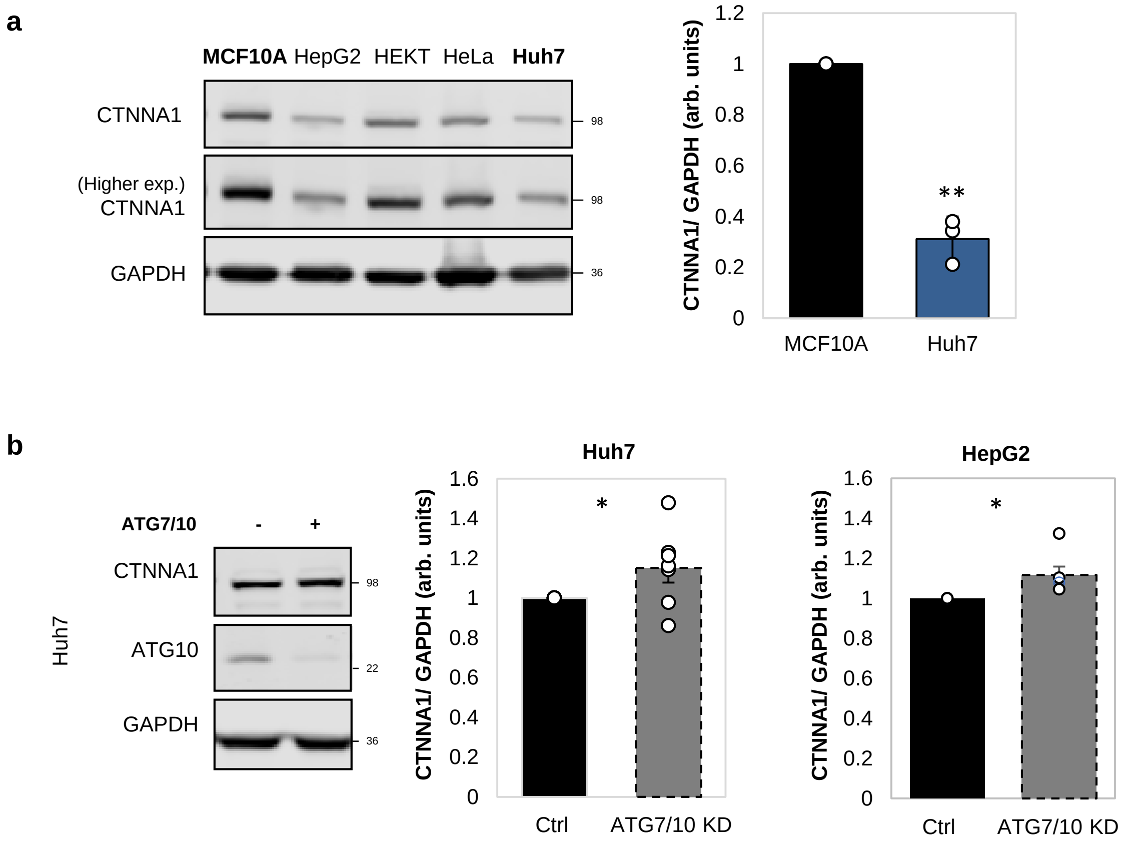

Supplementary Fig. 23 | α-catenin levels upon autophagy inhibition in Huh7 cells.

a, Representative immunoblot of CTNNA1 in various cell line, including Huh7. GAPDH was used as loading control. Bars represent the mean ± s.e.m. (n = 3 independent experiments; $^{**}P$ < 0.01; two-tailed one sample t-test).

b, Representative immunoblot of CTNNA1 levels in Huh7 cells exposed to ATG7/10 siRNAs. Bars represent the mean ± s.e.m. (n = 7 independent experiments; $^{*}P$ < 0.05; two-tailed one sample t-test). Data in HepG2 cells from Fig. 10a is shown enlarged on the right for relevant comparison.

Exact P values for asterisks: **a** 0.0055; **b** (from left to right) 0.0435, 0.0405.

Supplementary Fig. 24

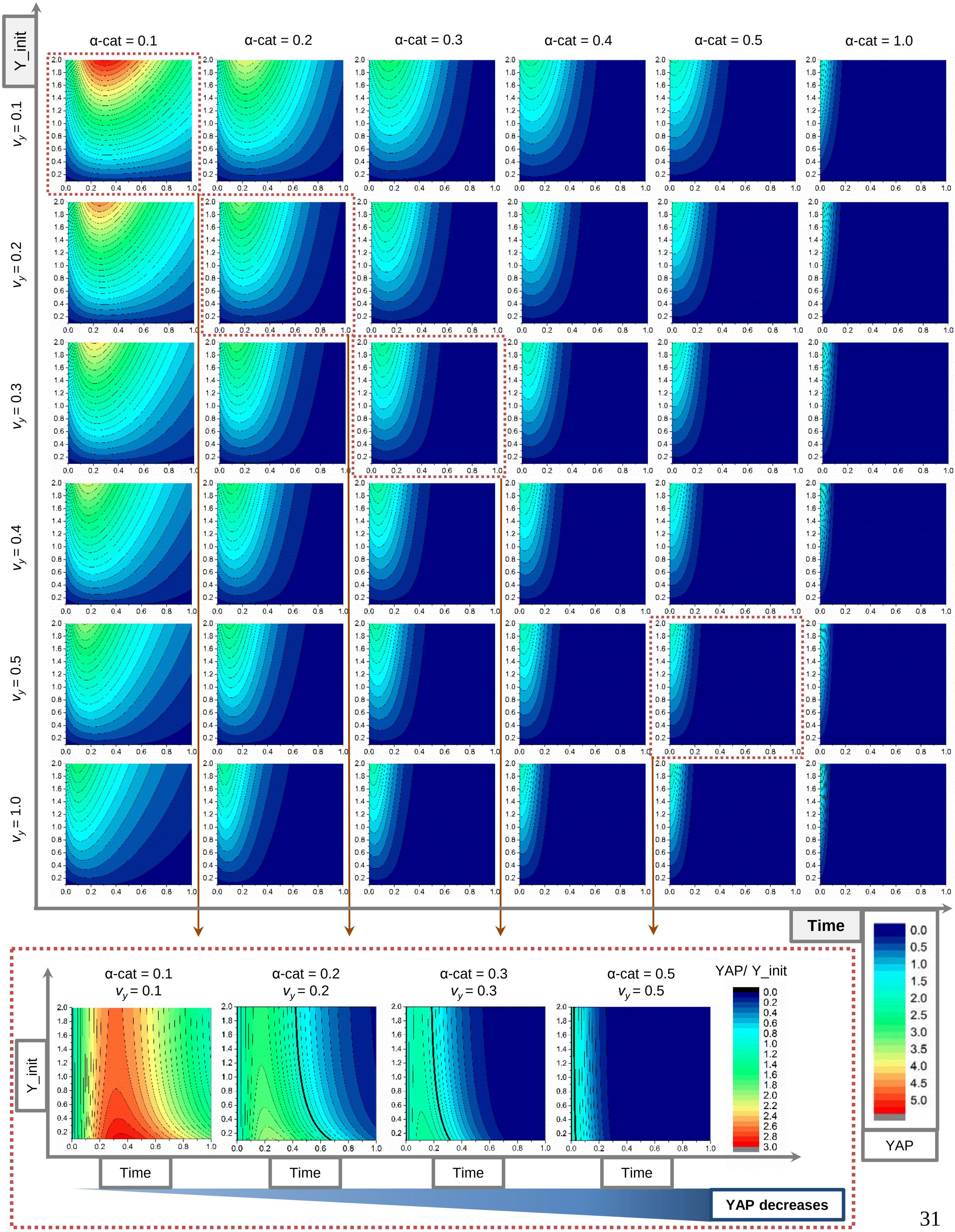

Supplementary Fig. 24 | Time variation of YAP/TAZ activity when different initial YAP, α-cat and v_Y values are considered upon autophagy downregulation.

Predicted time variation of absolute YAP/TAZ activity when various initial YAP (YAP_init), α-cat and v_Y values are considered (A_init = 1.0). Time parameter is shown on the horizontal axis, while the Y_init is varied on the vertical axis.

Initial α-cat levels have the strongest influence on final YAP/TAZ activity upon autophagy inhibition at lower time points. The colour code on the bottom right indicates the estimated absolute values of YAP/TAZ activity.

Predicted relative YAP/TAZ activity (to initial values) in the indicated conditions are shown on the graph on the bottom. In low initial α-cat and v_Y conditions, YAP/TAZ increases upon autophagy inhibition at any time, while increasing initial α-cat and v_Y conditions YAP/TAZ is reduced by autophagy inhibition at increasing time points.

Note: Y_init represents the initial value for YAP activity (before applying the autophagy perturbation); A_init represents the initial autophagy level (before applying the autophagy perturbation); α-cat represents the initial α-catenin level (before applying the autophagy perturbation); v_Y represents the strength of the feedback loop / the strength of YAP influence on autophagy levels (Pavel et al. 2018)); YAP represents the YAP activity level at the indicated time point of autophagy inhibition (after applying the autophagy perturbation).

Supplementary Fig. 25

a

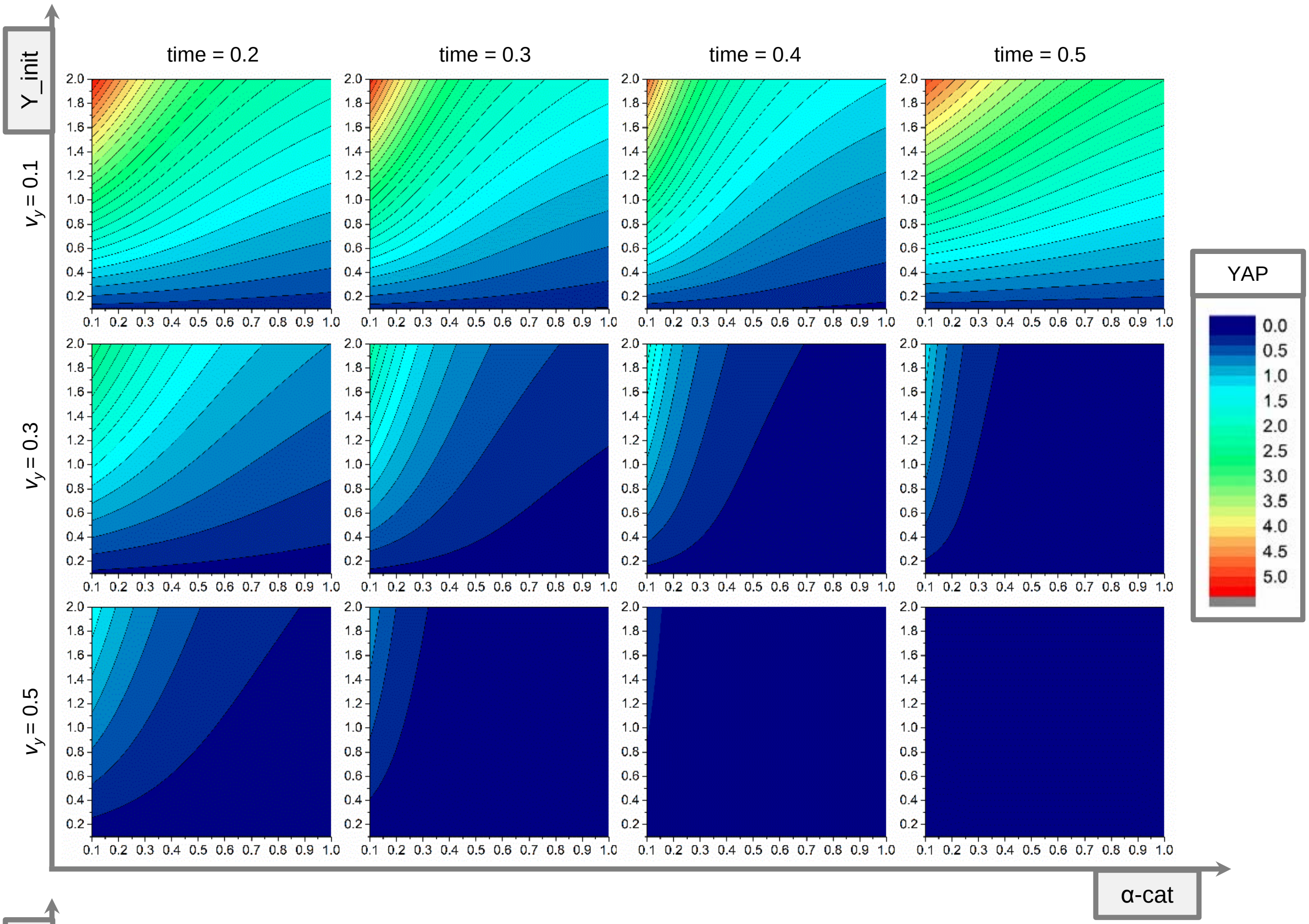

b

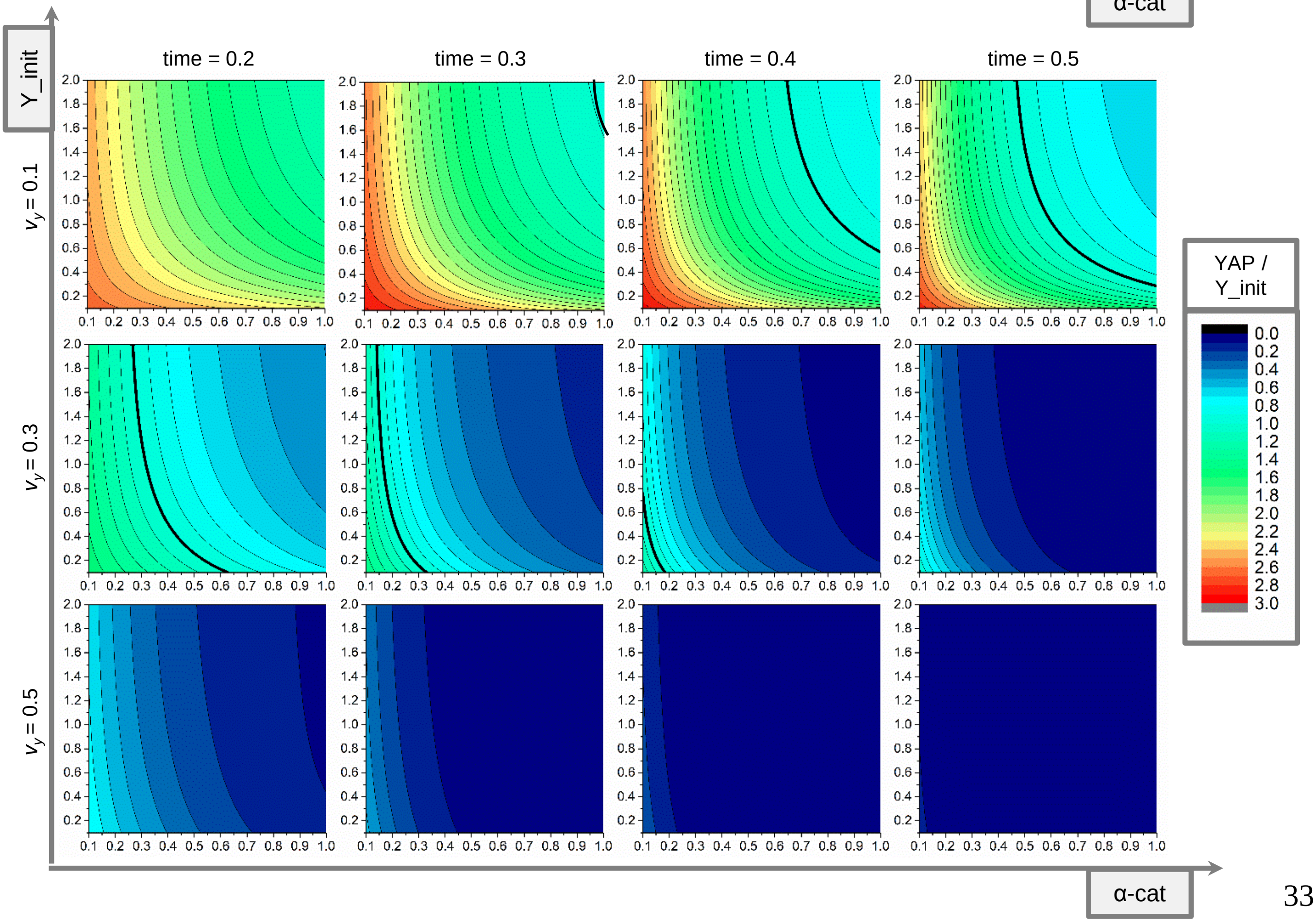

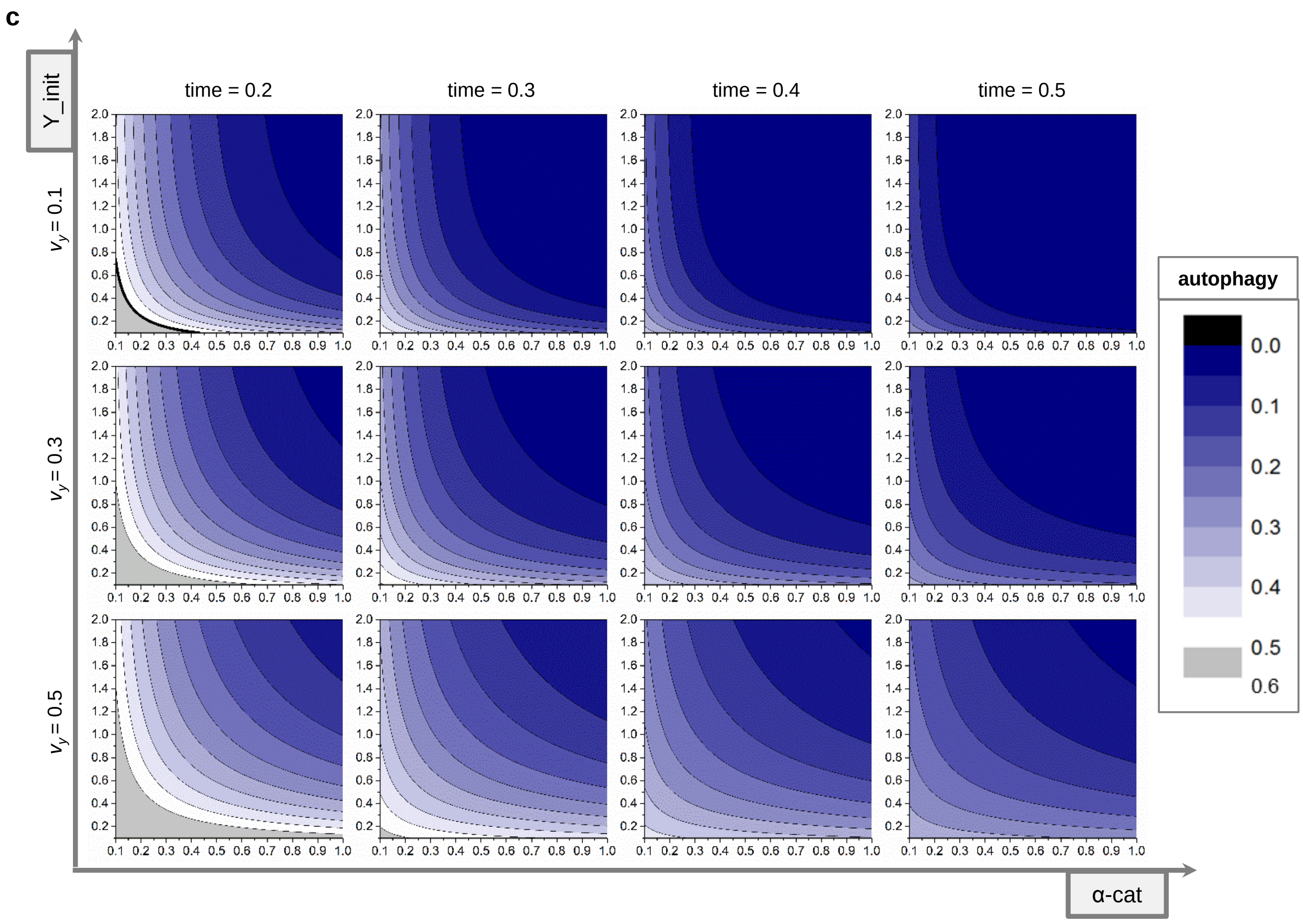

Supplementary Fig. 25 | YAP/TAZ activity at different time points of autophagy inhibition when initial YAP, α-cat and v_Y values are varied.

a, Predicted absolute YAP/TAZ activity when various initial YAP, α-cat and v_Y values are considered at different time points of autophagy inhibition (A_init = 1.0). α-cat parameter is shown on the horizontal axis, while the Y_init is varied on the vertical axis.

Initial v_Y levels have the strongest influence on final YAP/TAZ activity upon autophagy inhibition at higher time points. The colour code on the right indicates the estimated absolute values of YAP/TAZ activity.

b, Predicted relative YAP/TAZ activity (to initial values) for the conditions described at (**a**). The colour code on the right indicates the estimated relative values of YAP/TAZ activity. The black line corresponds to relative YAP = 1.0. The conditions with YAP values above the line are those that increase YAP upon autophagy inhibition.

c, Predicted relative autophagy levels for the indicated conditions in (**a**). The colour code on the right indicates the estimated relative autophagy values.

Note: Y_init represents the initial value for YAP activity (before applying the autophagy perturbation); A_init represents the initial autophagy level (before applying the autophagy perturbation); α-cat represents the initial α-catenin level (before applying the autophagy perturbation); v_Y represents the strength of the feedback loop / the strength of YAP influence on autophagy levels (Pavel et al. 2018)); YAP represents the YAP activity level at the indicated time point of autophagy inhibition (after applying the autophagy perturbation).

Supplementary Fig. 26

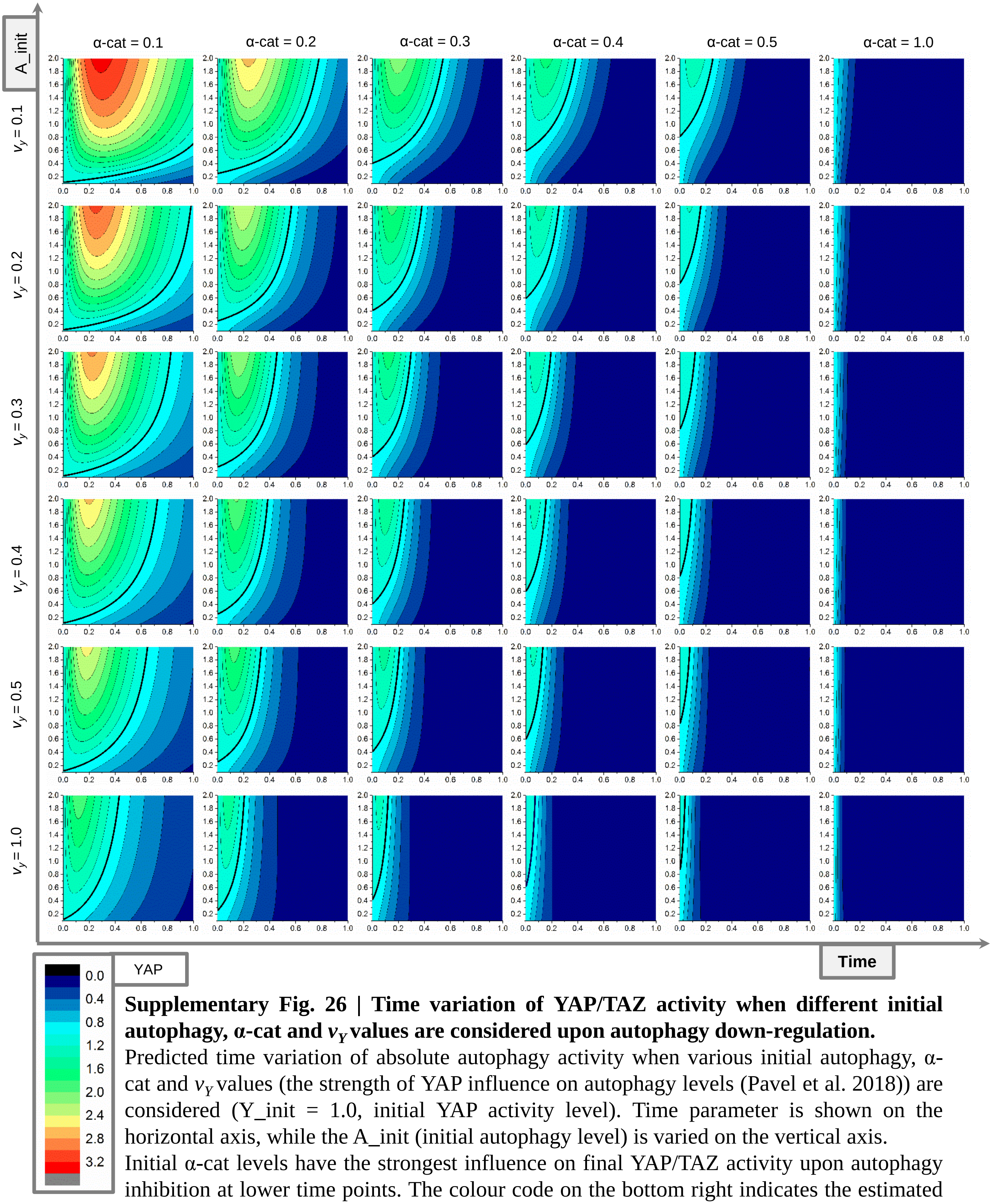

Supplementary Fig. 26 | Time variation of YAP/TAZ activity when different initial autophagy, α-cat and v_Y values are considered upon autophagy down-regulation. Predicted time variation of absolute autophagy activity when various initial autophagy, α-cat and v_Y values (the strength of YAP influence on autophagy levels (Pavel et al. 2018)) are considered (Y_init = 1.0, initial YAP activity level). Time parameter is shown on the horizontal axis, while the A_init (initial autophagy level) is varied on the vertical axis. Initial α-cat levels have the strongest influence on final YAP/TAZ activity upon autophagy inhibition at lower time points. The colour code on the bottom right indicates the estimated absolute values of YAP/TAZ activity.

Supplementary Fig. 27

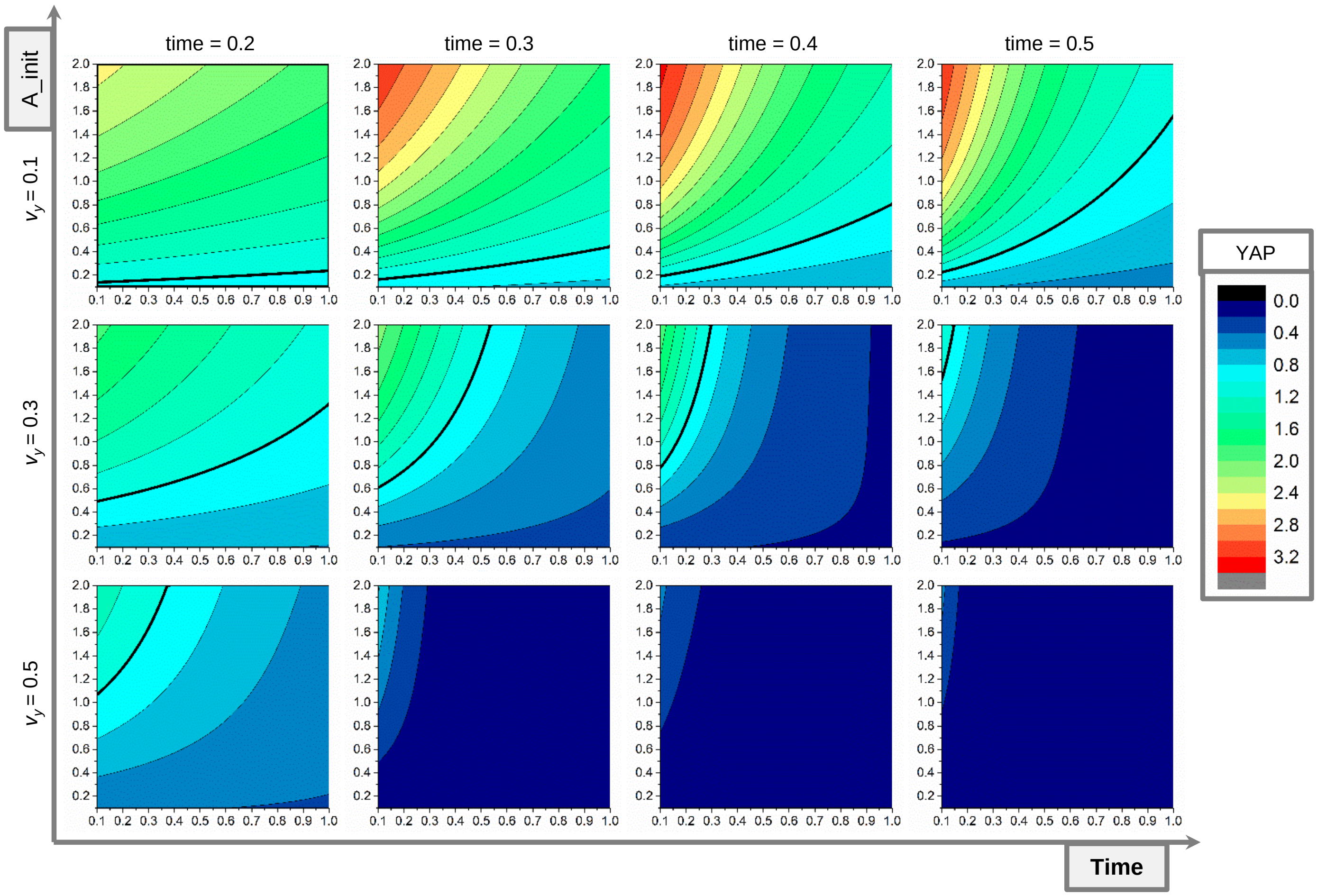

Supplementary Fig. 27 | YAP/TAZ activity at different time points of autophagy inhibition when initial autophagy, α-cat and v_Y values are varied.

Predicted relative YAP/TAZ activity (to initial values) for the indicated conditions activity when various initial autophagy, α-cat and v_Y values are considered at different time points of autophagy inhibition (Y_init = 1.0). Time parameter is shown on the horizontal axis, while the A_init is varied on the vertical axis.

The colour code on the right indicates the estimated relative values of YAP/TAZ activity. The black line corresponds to relative YAP = 1.0. The conditions with YAP values above the line are those that increase YAP upon autophagy inhibition.

Note: Y_init represents the initial value for YAP activity (before applying the autophagy perturbation); A_init represents the initial autophagy level (before applying the autophagy perturbation); α-cat represents the initial α-catenin level (before applying the autophagy perturbation); v_Y represents the strength of the feedback loop / the strength of YAP influence on autophagy levels (Pavel et al. 2018)); YAP represents the YAP activity level at the indicated time point of autophagy inhibition (after applying the autophagy perturbation).

Supplementary Fig. 28

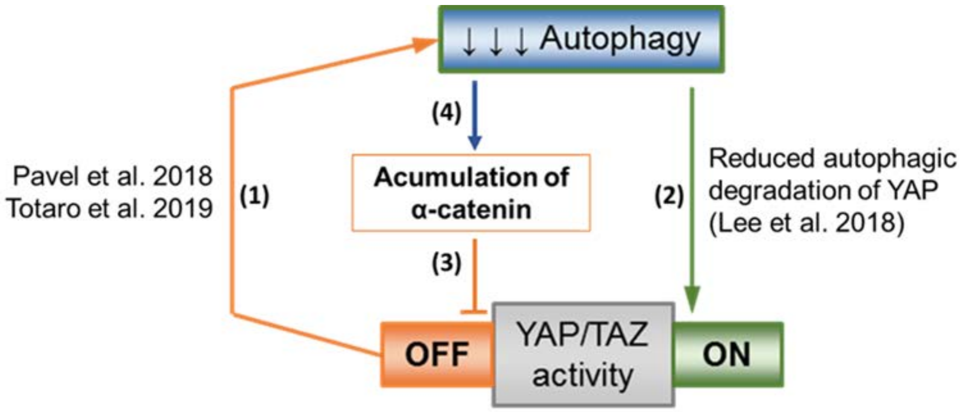

Supplementary Fig. 28 | Extended explanation of the mathematical model

The mathematical model proposed for understanding the dynamics of these 3 interlinked variables (autophagy, YAP activity and α-catenins) is based on the following **4 hypotheses**:

1. YAP promotes autophagy (YAP increase induces autophagy and YAP decrease inhibits autophagy – Pavel et. al. 2018)

2. Autophagy promotes *direct* YAP inhibition (autophagy induction directly decreases and inhibits YAP, autophagy inhibition directly increases and activates YAP – Lee et. al 2018)

3. α-catenins inhibits YAP (accumulation of α-catenins decreases YAP, α-catenin depletion and degradation increases YAP – Schlegelmilch et al. 2011)

4. Autophagy degrades α-catenins (*the novel mechanism described in this paper*: LIR-dependent degradation of α-catenins).

Note: Combining hypotheses 3 and 4 implies that **autophagy *indirectly* promotes YAP activation**.

Starting from those 4 hypotheses, we developed a dynamic system of **3 coupled differential equations** which follow the time evolution of our 3 inter-linked variables: autophagy (A), YAP activity (Y) and α-catenins (C). As the system does not have an analytical solution, it can only be numerically solved in order to provide a final solution and describe the temporal evolution (dynamics) of the process. Therefore, being a dynamic process, each time point depends on the previous one and the rate of evolution for each component (A, Y, C) at a certain time-point depends on their time-point values. The evolution rate is mediated by various weights, included as various coefficients in front of each term of the 3 differential equations (see *Methods* section)

More precisely, the system of differential equations that we present describes the rate of evolution (the time variation) for each component A, Y and C, when A is externally perturbed by genetic or chemical manipulation (direct repression of autophagy by inhibiting key autophagy genes or chemically impairing autophagy degradation and/or autophagosome formation). The 3 differential equations are detailed in the Methods section and they describe the following biological processes:

i) The **rate of evolution for autophagy** (the time variation of A variable) which depends on both the *external perturbation* described above (the first term in the equation (1) described in Methods section) and *the effect provided by YAP/TAZ on autophagy* (the second term in equation 1, which corresponds to ***hypothesis 1***). The effect of YAP/TAZ on autophagy may be either strong or weak, depending on cell line, environmental conditions, important aspects that we considered in our numerical simulations.

ii) The **rate of evolution for YAP activity** (the time variation of *Y* variable) which depends on the difference between: i) *the rate of accumulation of YAP provided by the* ***direct*** *effect of autophagy depletion* (explained by the second term in the differential equation (2), which corresponds to **hypothesis 2**), and ii) *the rate of inhibition of YAP by accumulating α-catenins, an* ***indirect*** *effect of autophagy depletion* (the first term in differential equation (2), which corresponds to **hypothesis 3**).

Here, we can easily observe that **if the catenin levels are high** *(at a certain time point)*, the effect of the first term (the effect of reducing YAP upon accumulation of α-catenins, which is an *indirect effect of autophagy depletion*) is strong enough to overcome the accumulation of YAP given by the second term (*a direct effect of autophagy depletion*) and the **cumulative effect of autophagy depletion** on YAP is **reduced YAP activity**.

If the **catenin levels are low** (*at a certain time point*), the first term in the differential equation (2) (the *indirect inhibition of YAP by autophagy depletion*) has a lower effect than the second term (which shows the *direct accumulation of YAP upon autophagy depletion*) and thus the **cumulative effect of autophagy depletion** on YAP is **increased YAP activity**.

iii) The **rate of evolution for α-catenins** (the time variation of *C* variable) includes the accumulation of the *C* component induced by autophagy inhibition (which correspond to **hypothesis 4** and includes *the novel LIR-dependent degradation mechanism by autophagy proposed in this paper*). The accumulation of α-catenins is saturated to the value determined experimentally when the system reaches its equilibrium (after 5 days of autophagy key genes knockdowns, Fig. 3 and Supplementary Fig. 9).

For a better understanding of this complex process, here we proposed to further dissect 2 main extreme situations of **high** and **low** initial α-catenins levels.

High initial C values.

When **initial *C*** values are **high**, the ***initial tendency for YAP is to decrease*** in differential equation (2), as the effect of the first term (corresponding to **hypothesis 3**: *autophagy inhibition* ***indirectly*** *inhibits YAP*) is higher than the effect provided by the second term (corresponding to **hypothesis 2**: *autophagy inhibition* ***directly*** *increases YAP*). As the *C values increase over time,* the *strength of the first term also increases* (more α-catenins in the cytosol, more YAP/TAZ sequestered and inactivated while autophagy decreases), while the rate of directly induced YAP accumulation by autophagy inhibition decreases over time (as this rate is proportional to the time-point YAP value). Thus, the **cumulative effect of autophagy inhibition** over YAP is **reduced YAP activity** (for the entire duration of the process).

Low initial C values.

When **initial** *C* values are **low**, the ***initial tendency for YAP is to increase*** in differential equation (2), as the effect of the first term (corresponding to **hypothesis 3**: *autophagy inhibition* ***indirectly*** *inhibits YAP*) is lower than the effect provided by the second term (corresponding to **hypothesis 2**: *autophagy inhibition* ***directly*** *increases YAP*). Thus, **an initial cumulative effect of autophagy inhibition** over YAP is **increased YAP activity**. However, as the *C values increase over time*, the *strength of the first term also increases* (more catenins in the cytosol, more YAP/TAZ sequestered and inactivated, while autophagy decreases), eventually reaching a time point when *the effect of the first term is equal to the second term* (corresponding to ***the peak of the graph of YAP variation over time***, Supplementary Fig. 22a)

and *even overcoming it*, determining YAP reduction over time if the system would be kept under such circumstances long enough, and, then, the **final possible cumulative effect of autophagy inhibitio***n* over YAP may be even the opposite: **reduced YAP activity**.

However, from an experimental point of view, it is difficult to perfectly control the time-point dependency of such a complex system, as environmental conditions may vary over time, as changes (of various degrees) in the cell growth, confluence, nutrients availability, or even in the strength of YAP effect on autophagy may influence the weights of the considered coefficients in our system of differential equations. The proposed model only aims to better understand how perturbing autophagy may have opposite effects on cellular signaling pathways in different cell types, by reducing the cellular systems to our 4 hypotheses - this allows us to describe how YAP/TAZ activity is influenced by the initial levels of α-catenins (when such perturbations on autophagy are occurring).

Supplementary Fig. 29

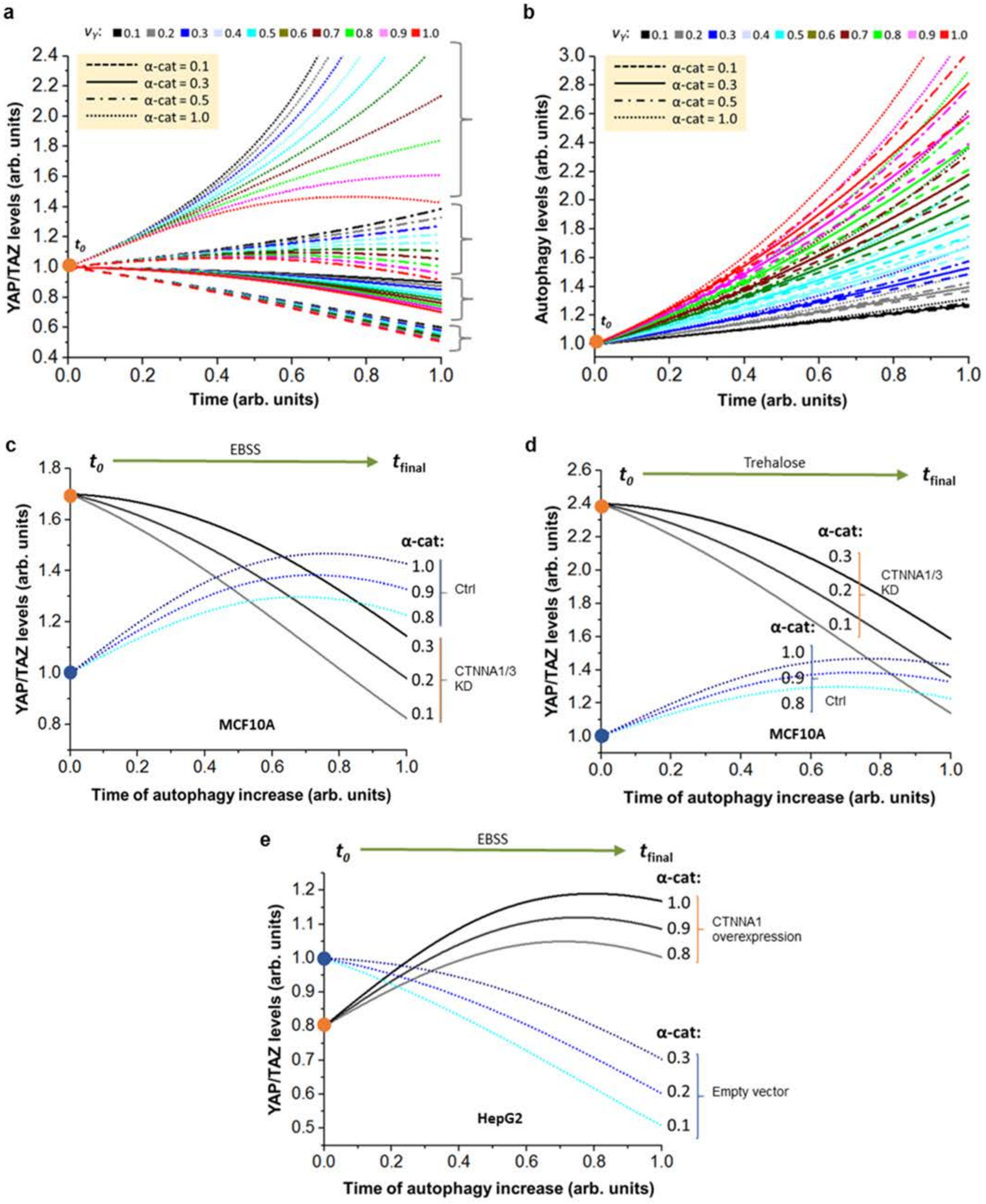

Supplementary Fig. 29 | Predicted time variation of YAP/TAZ activity upon autophagy up-regulation.

a, Predicted YAP/TAZ activity in cells with varying α-cat values (0.1, 0.3, 0.5 or 1.0) upon autophagy up-regulation.

b, Predicted time-dependent autophagy increase in cells with varying α-cat values (0.1, 0.3, 0.5, 1.0) upon external applied autophagy stimuli.
c, Predicted YAP activity upon autophagy upregulation, related to Fig. 10b (control and CTNNA1/3 KD MCF10A cells exposed to EBSS).
d, Predicted YAP activity upon autophagy upregulation, related to Fig. 10c (control and CTNNA1/3 KD MCF10A cells exposed to Trehalose).
e, Predicted YAP activity upon autophagy upregulation, related to Fig. 10e (empty-vector and mEm-CTNNA1 overexpressing HepG2 cells exposed to EBSS).

Note: The differences observed upon autophagy stimulation between the modelling simulations from (**c**) and (**d**), are due to differences in the extent of TEAD-luciferase activity increase (1.7 in one experiment - see Fig. 10b, and 2.4 in the other experiment – see Fig. 10c), most likely caused by differences in the CTNNA1/3 knockdown efficiencies from experiments performed in different days, from different cell passages.
α-cat represents the initial α-catenin level (before applying the autophagy perturbation); v_Y represents the strength of the feedback loop / the strength of YAP influence on autophagy levels (Pavel et al. 2018)); YAP represents the YAP activity level at the indicated time point of autophagy inhibition (after applying the autophagy perturbation).

Supplementary Fig. 30

a

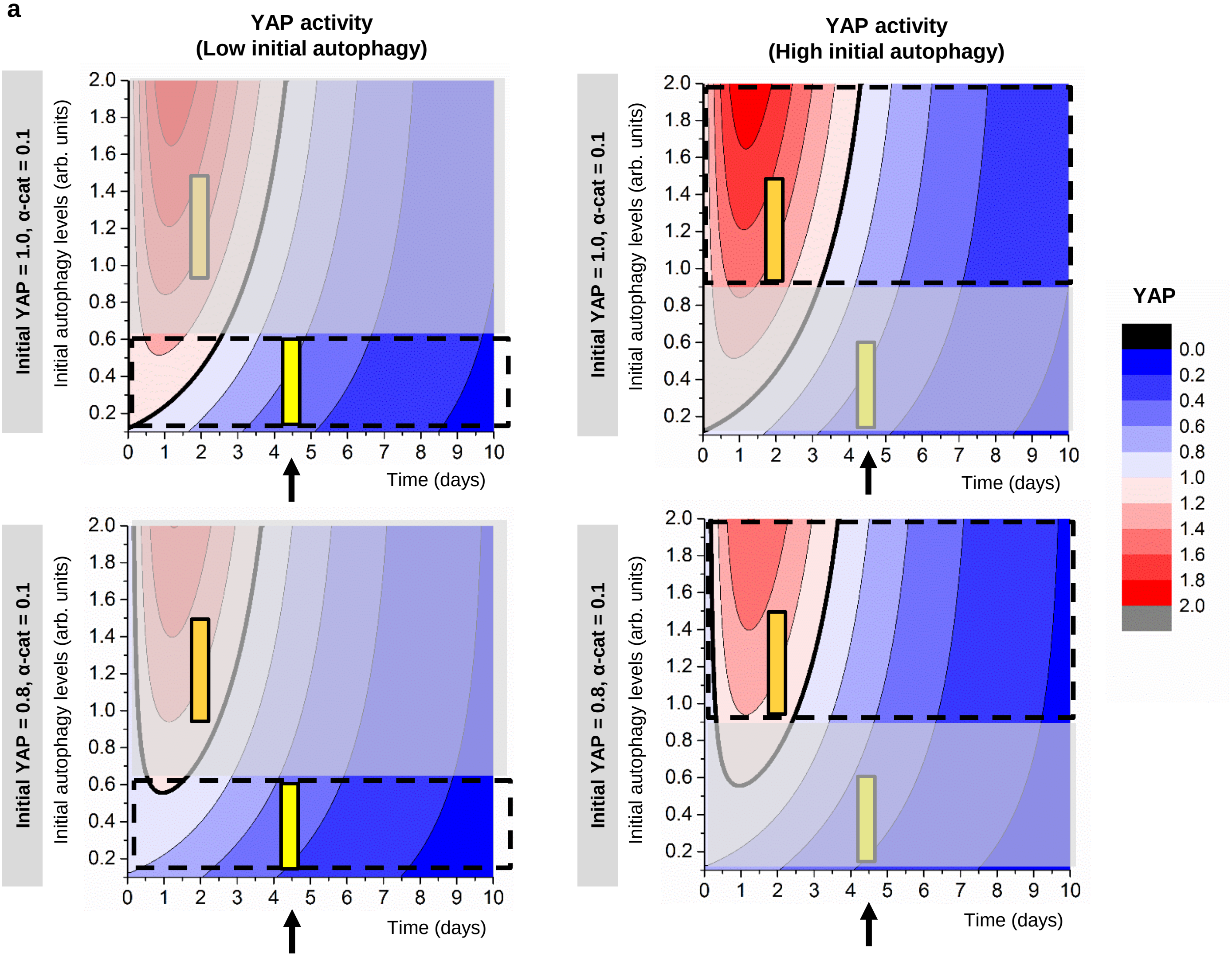

<u>**Low α-catenins:**</u>

- Autophagy levels **inverse correlate** with YAP activity at <u>lower time points</u>
- Autophagy levels **positively correlate** with YAP activity at <u>higher time points (96 h)</u>

b

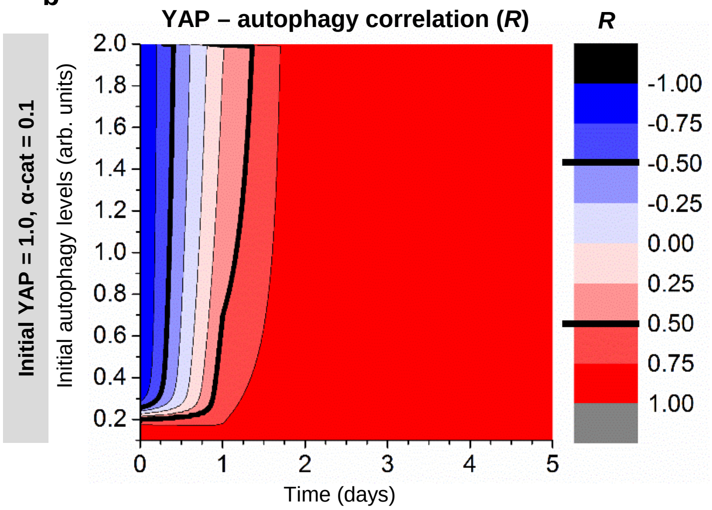

c

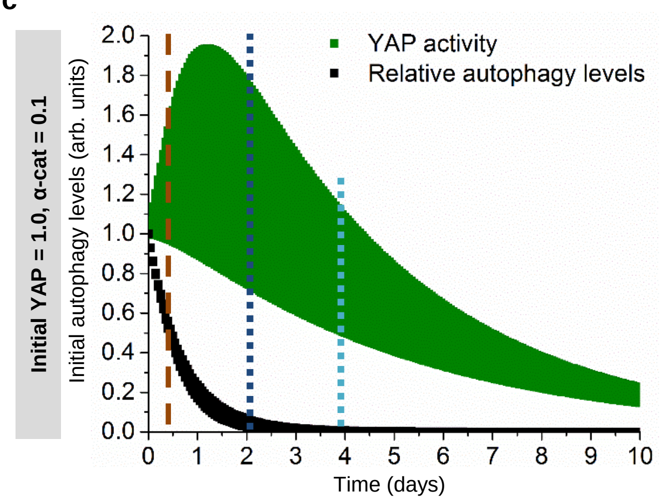

Supplementary Fig. 30 | Time variation of YAP/TAZ activity in cells with low α-cat levels in low and high initial autophagy conditions upon autophagy down-regulation.

a, Left - Predicted YAP/TAZ activity in cells with low α-cat (0.1) and already low initial autophagy: potential increased YAP/TAZ activity after autophagy down-regulation at early time points, but reduced YAP/TAZ activity at later time points, related to experimental data from Fig. 9a-d. Right - Predicted YAP/TAZ activity in cells with low α-cat (0.1) and high initial autophagy: increased YAP/TAZ activity after autophagy down-regulation, related to experimental data from Fig. 10d.

Note: When initial α-cat values are low, the initial tendency for YAP is to increase as the direct effect of up-regulating YAP by autophagy inhibition (YAP accumulates due to its impaired autophagic degradation) overcomes the indirect effect of inhibiting YAP by autophagy depletion (the herein presented α-catenins-mediated mechanism). However, as the α-cat values accumulates over time in the autophagy-deficient cells, more YAP is sequestered into the cytosol and inactivated, as the indirect effect overcomes the previous direct effect. Thus, the cumulative effect at higher time points of autophagy inhibition is of reducing YAP activity.

b, Time-dependent correlation (*R* - Pearson correlation coefficient) between YAP activity and autophagy levels in cells with low initial α-cat (0.1). At early time points: YAP inversely correlates with autophagy (YAP increases upon autophagy reduction); at later time points: YAP positively correlates with autophagy (YAP decreases upon autophagy reduction).

c, Time-dependency of YAP-activity and autophagy values in cells with low initial α-cat (0.1). The same observations as in (**b**).

Supplementary Fig. 31

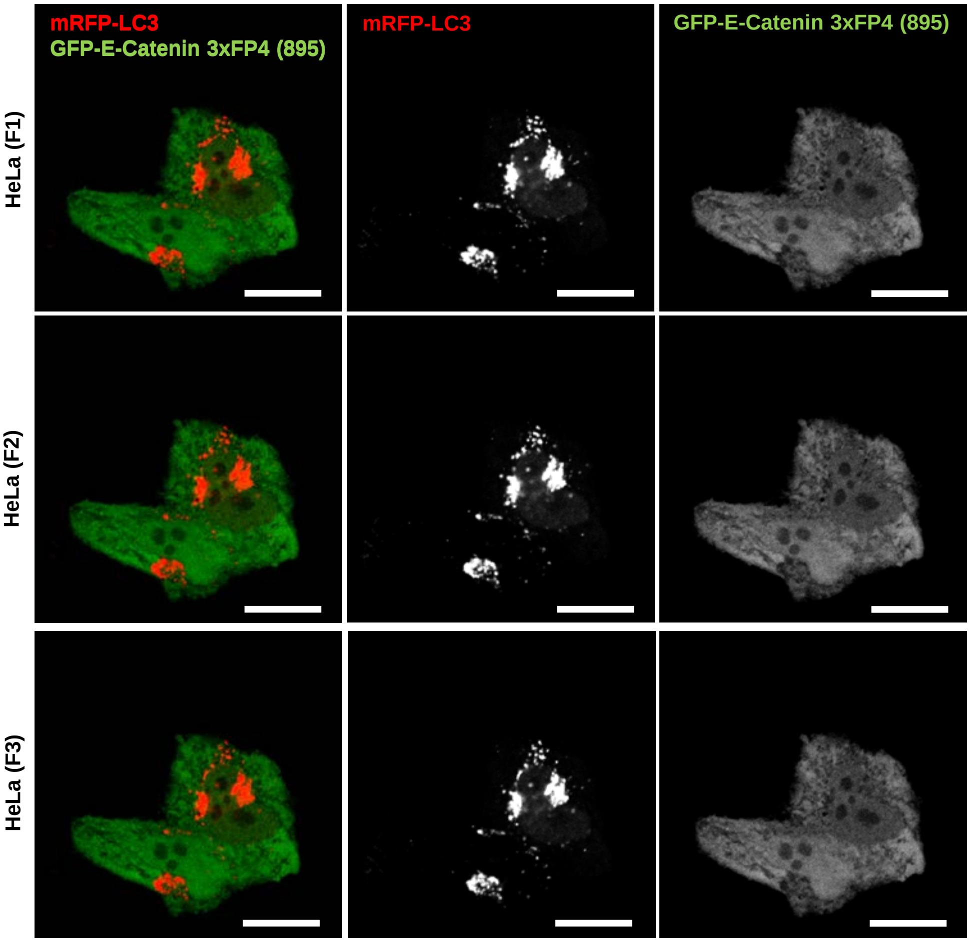

Supplementary Fig. 31 | The α-E-catenin(1-895) mutant does not accumulate in autophagosomes in HeLa cells.

Confocal live imaging of HeLa cells expressing GFP-α-E-Catenin3xFP4(1-895) and the autophagosome marker mRFP-LC3. Scale bars are 10 µm. The experiment was repeated once with similar results.

Supplementary Fig. 32

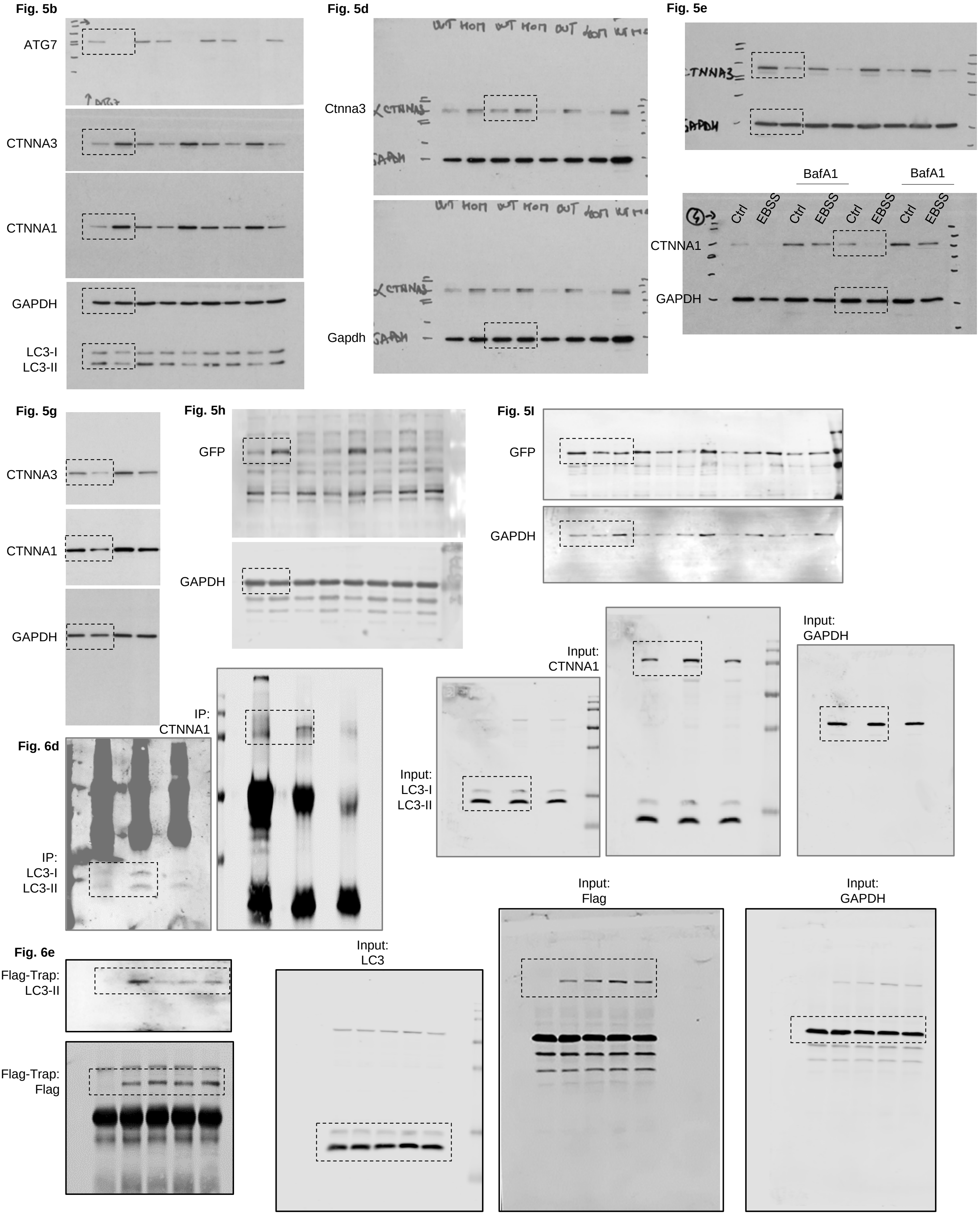

Supplementary Fig. 32 - continued

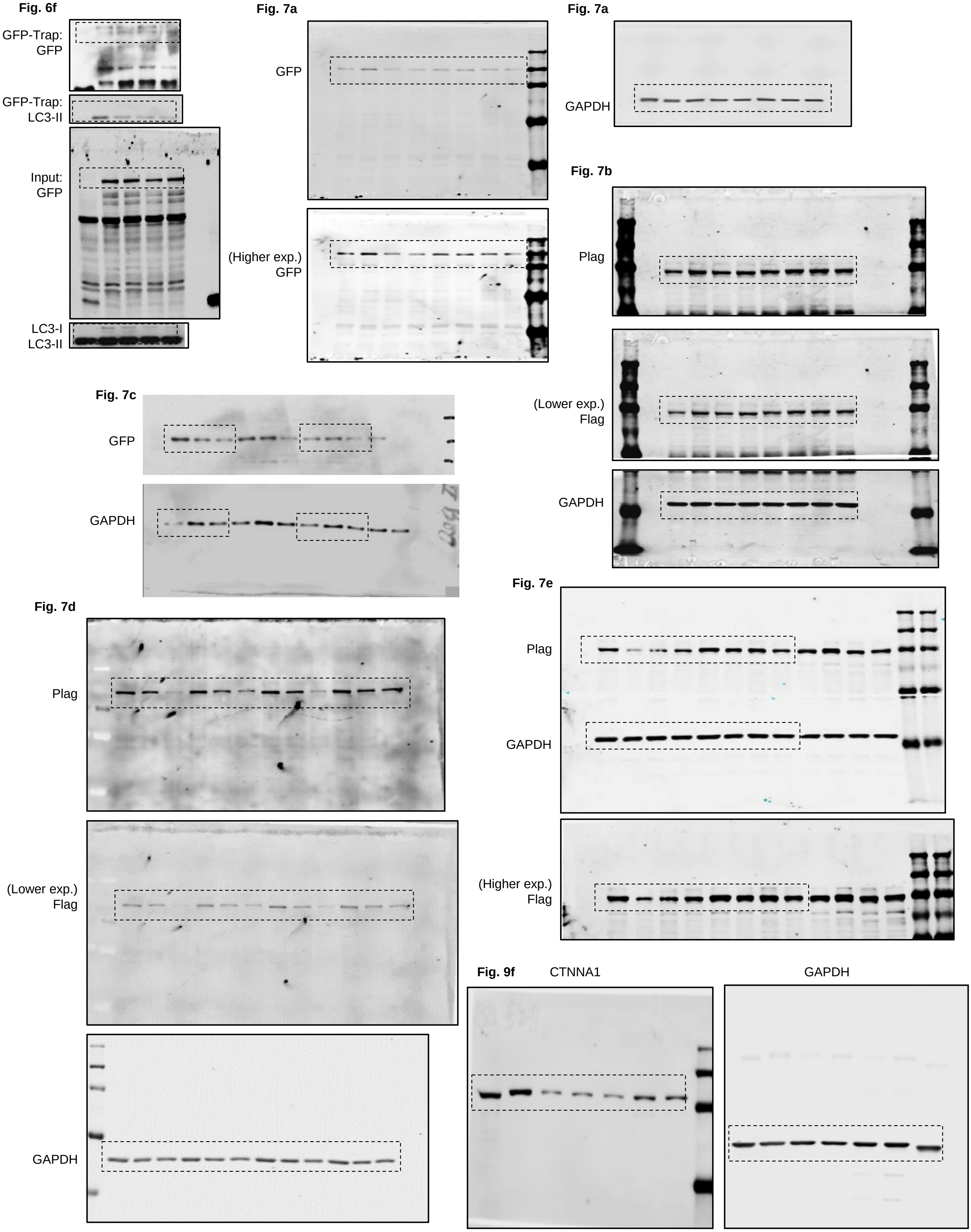

Supplementary Fig. 32 - continued

Fig. 10a

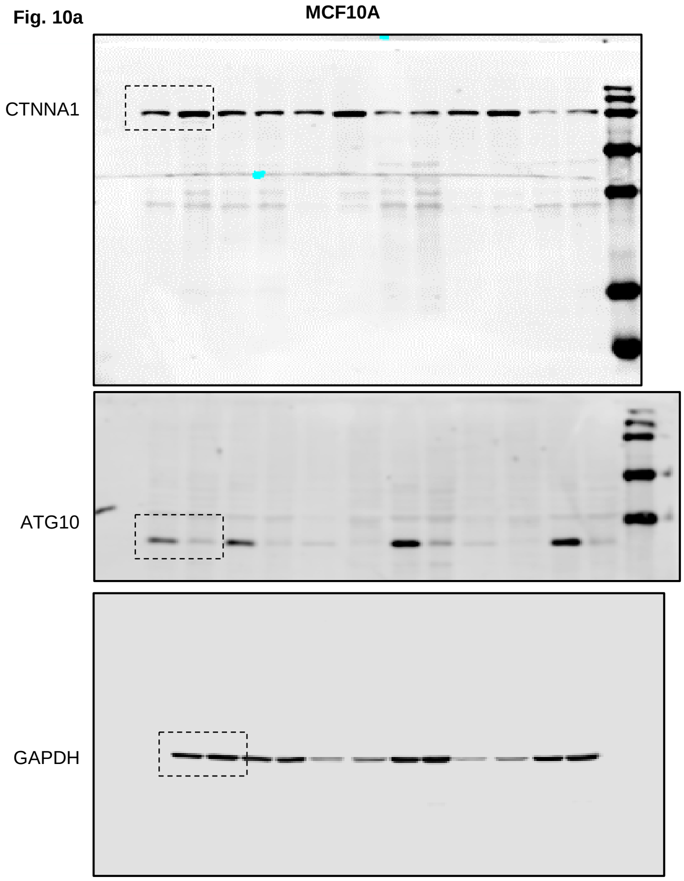

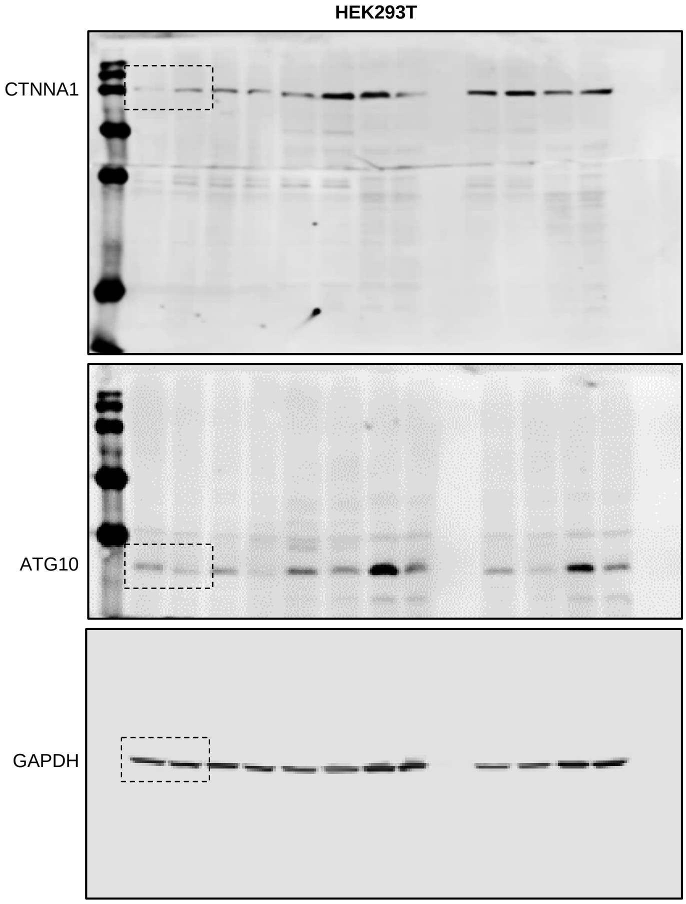

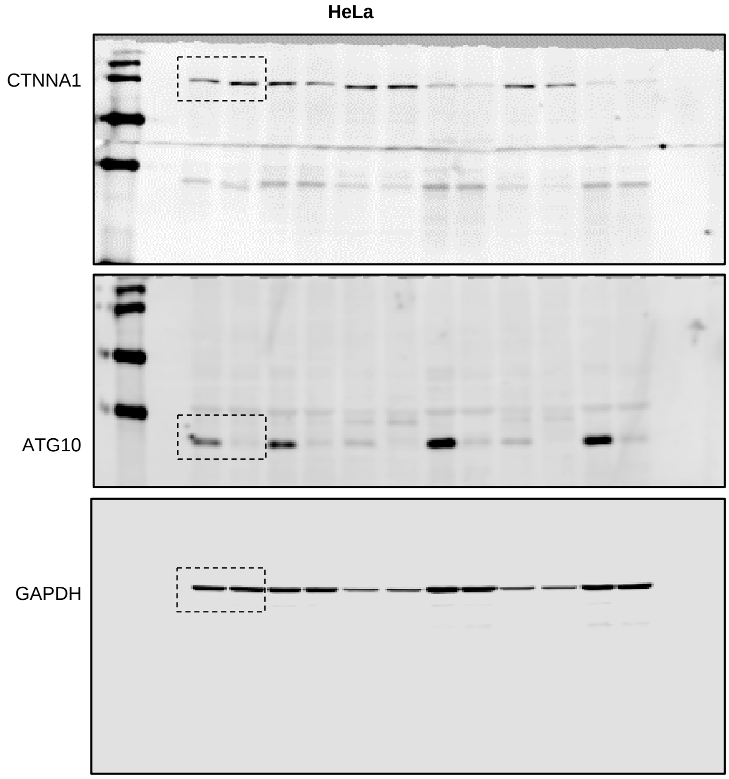

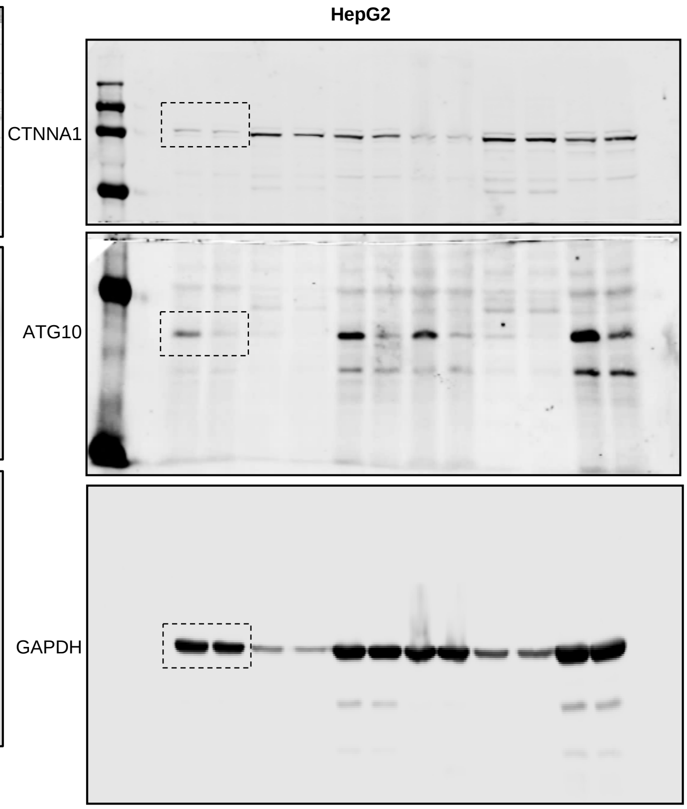

Fig. 10d

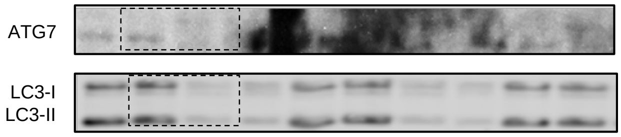

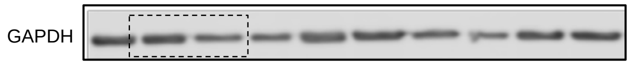

Supplementary Fig. 1a

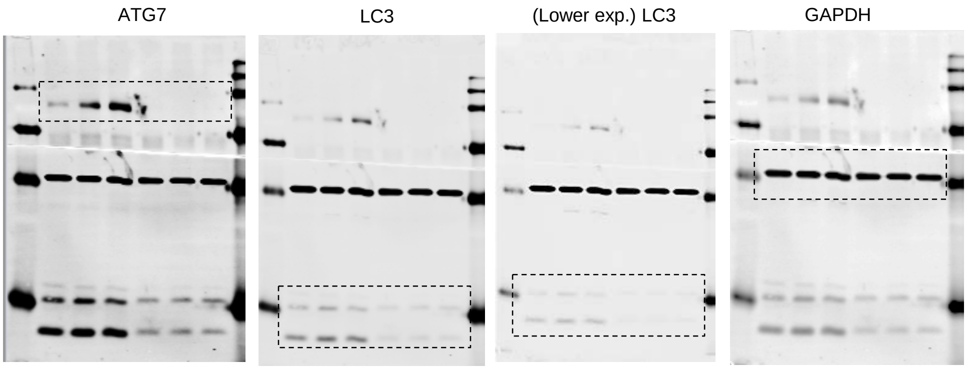

Supplementary Fig. 1d

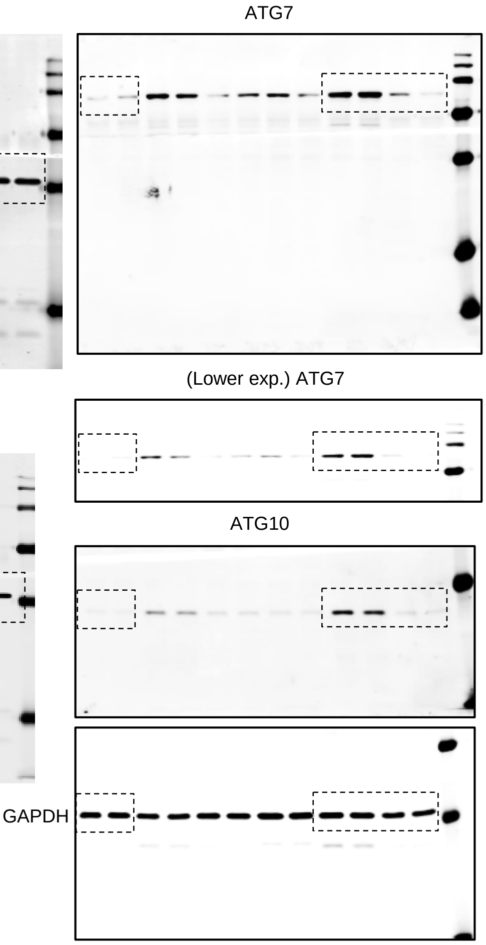

Supplementary Fig. 1b

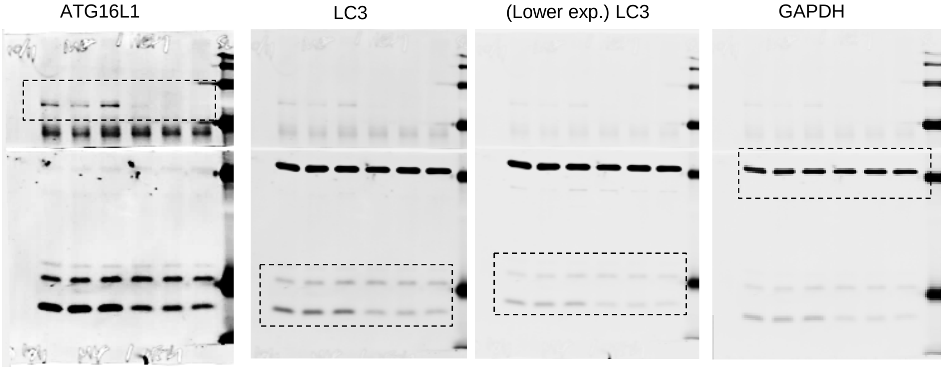

Supplementary Fig. 1d

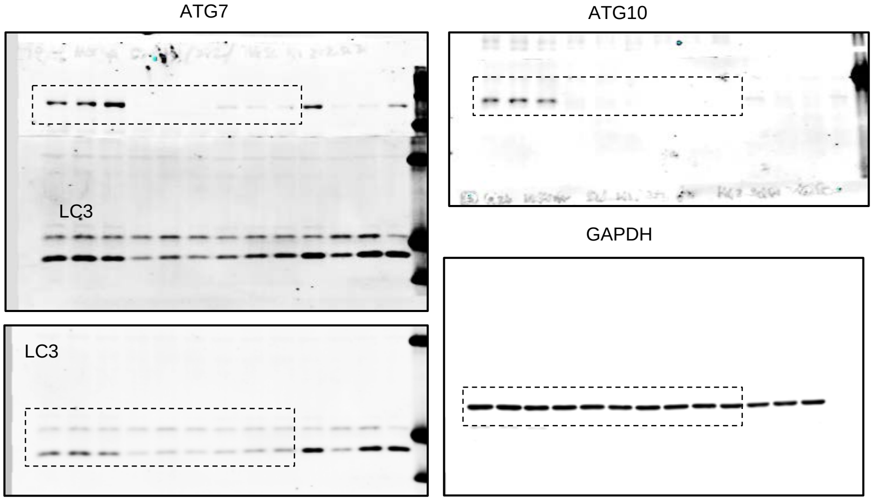

Supplementary Fig. 2e

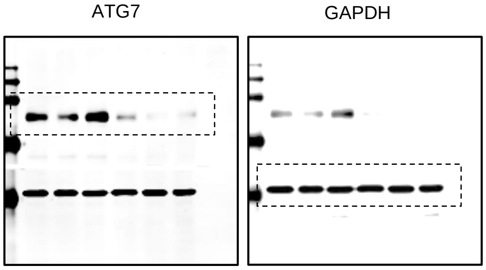

Supplementary Fig. 3a

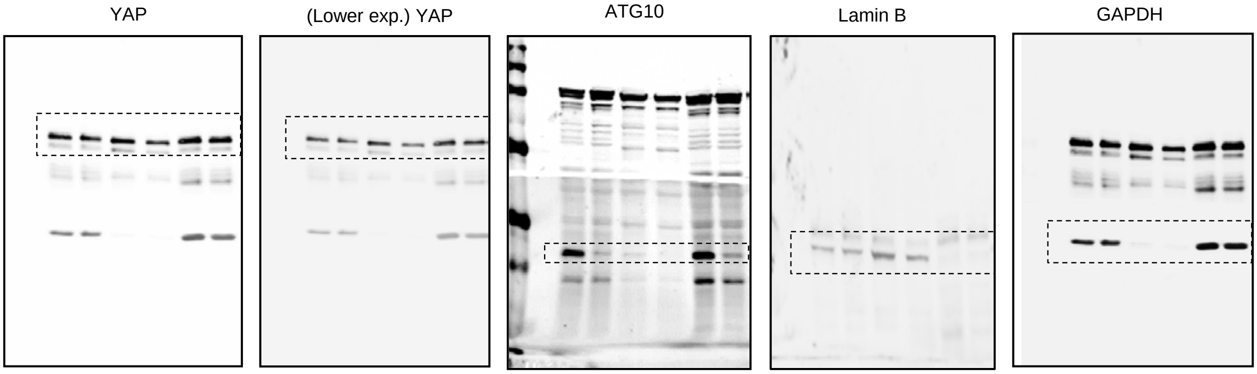

Supplementary Fig. 3b

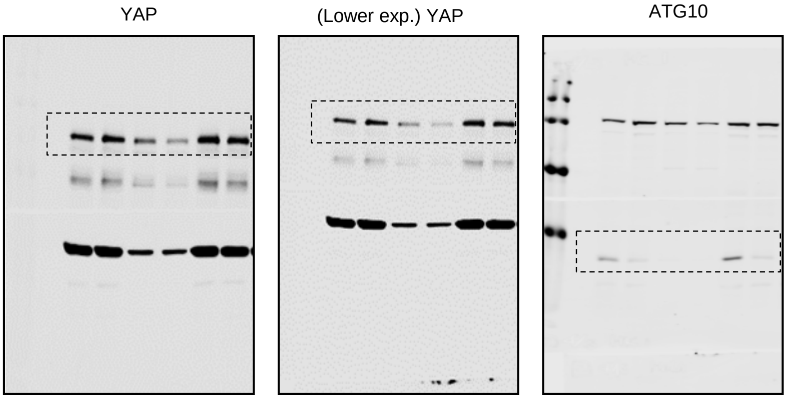

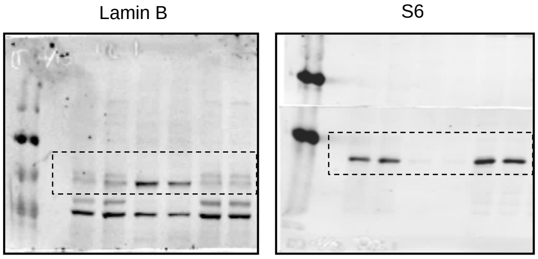

Supplementary Fig. 4e

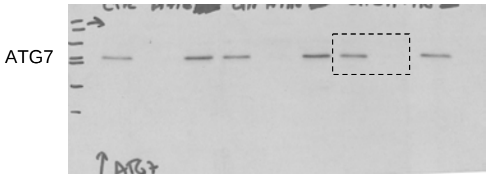

Supplementary Fig. 3b

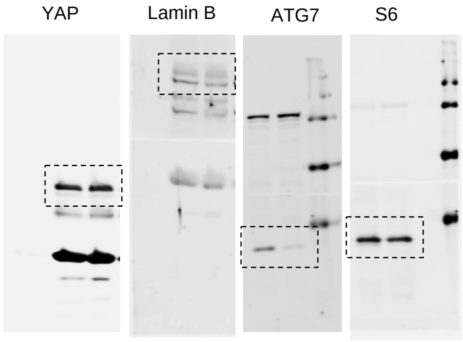

Supplementary Fig. 4e

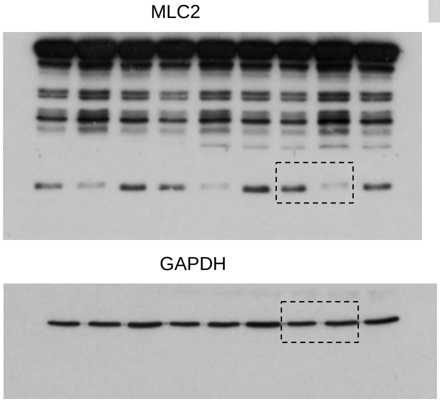

Supplementary Fig. 4f

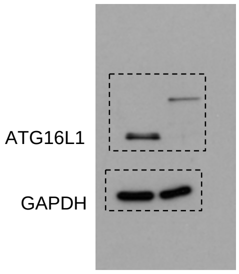

Supplementary Fig. 8c (left)

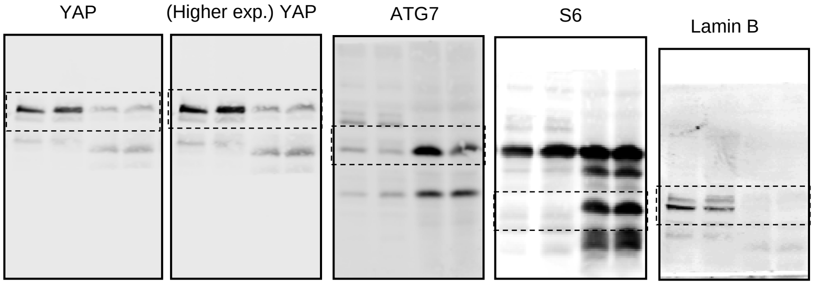

Supplementary Fig. 8g

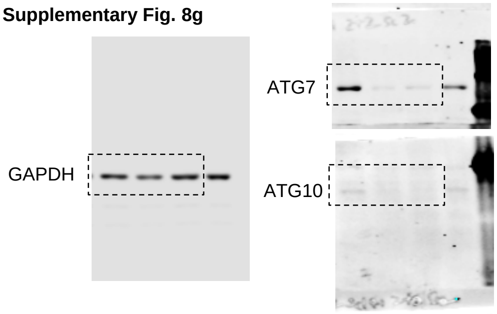

Supplementary Fig. 8c (right)

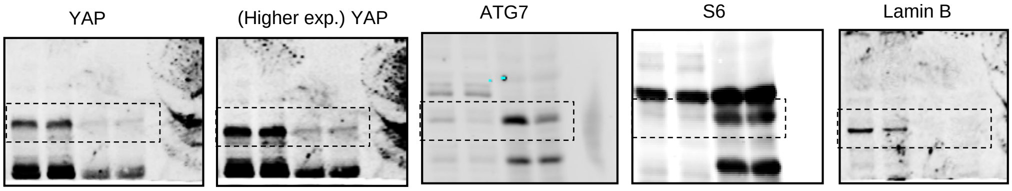

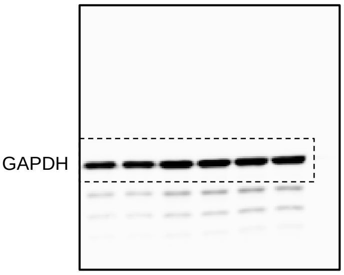

Supplementary Fig. 9b

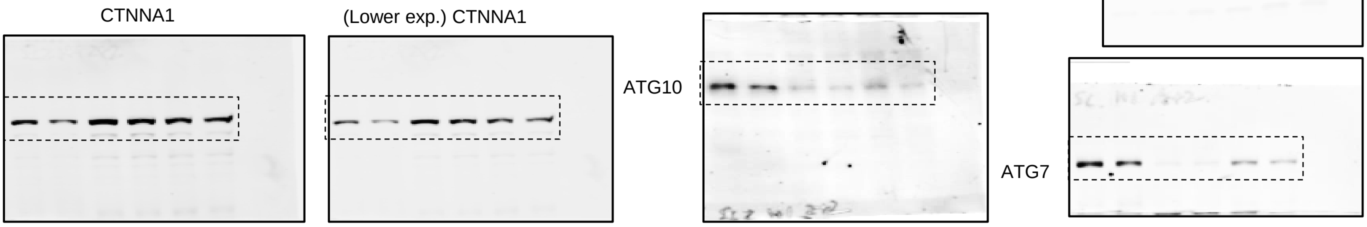

Supplementary Fig. 9c

CTNNA1 | (Lower exp.) CTNNA1 | GAPDH | ATG7 | ATG10

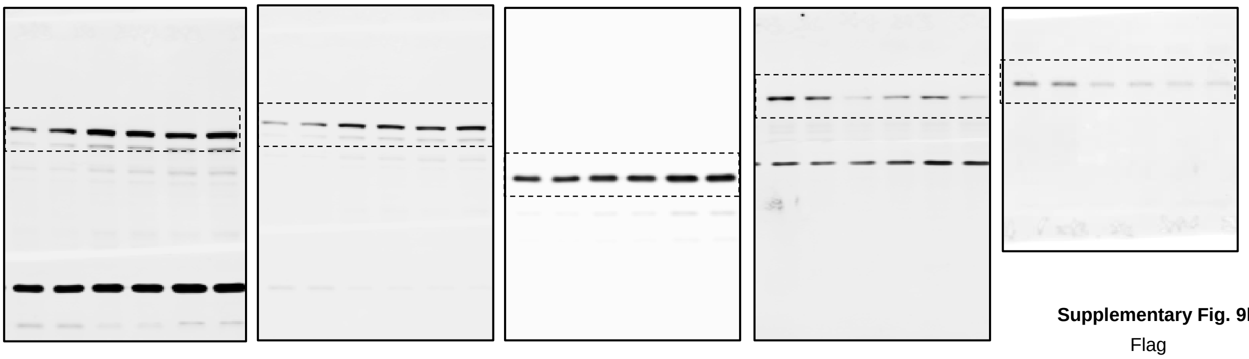

Supplementary Fig. 9d

CTNNA1

LC3

GAPDH

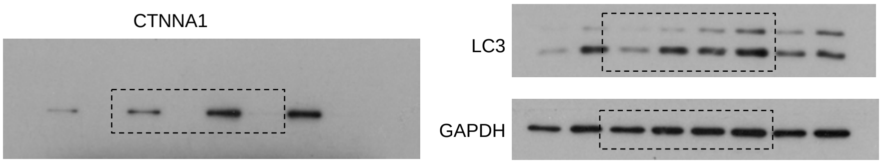

Supplementary Fig. 9h

Flag

(Higher exp.) Flag

GAPDH

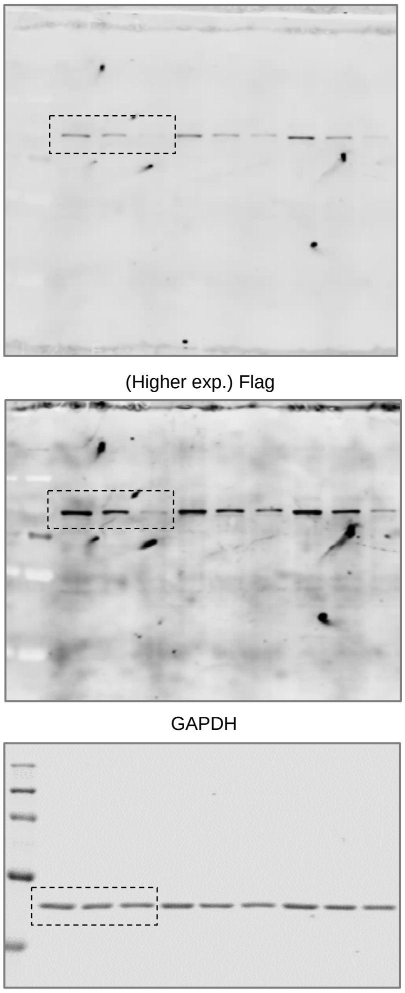

Supplementary Fig. 9e

CTNNA3

CTNNA1

GAPDH

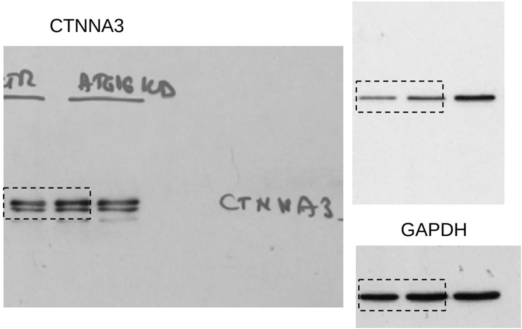

Supplementary Fig. 9g

Ctnna3

Gapdh

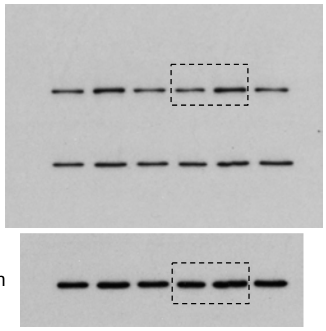

Supplementary Fig. 9i

Flag

(Higher exp.) Flag

GAPDH

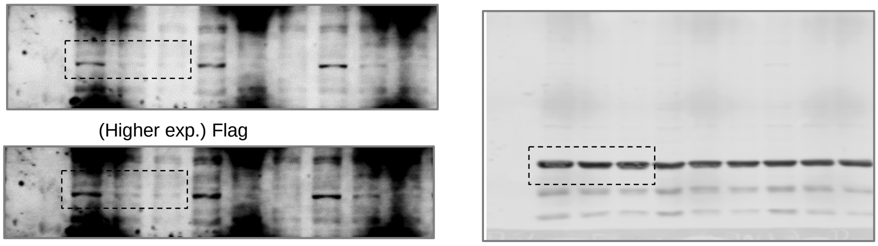

Supplementary Fig. 12a

#1 | #2 | #3

CTNNA1

LC3

CTNNA1

LC3

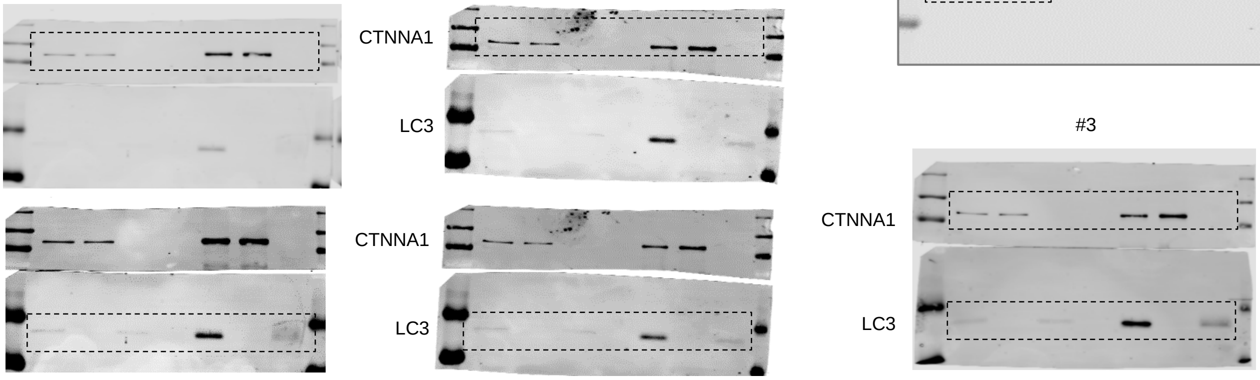

Supplementary Fig. 32 - continued

Supplementary Fig. 12b

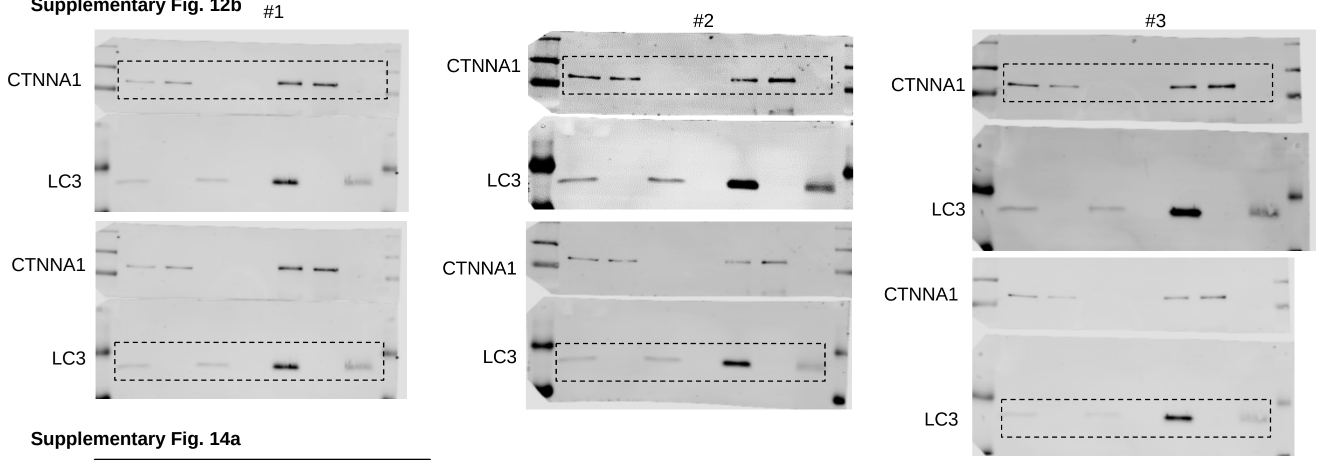

Supplementary Fig. 14a

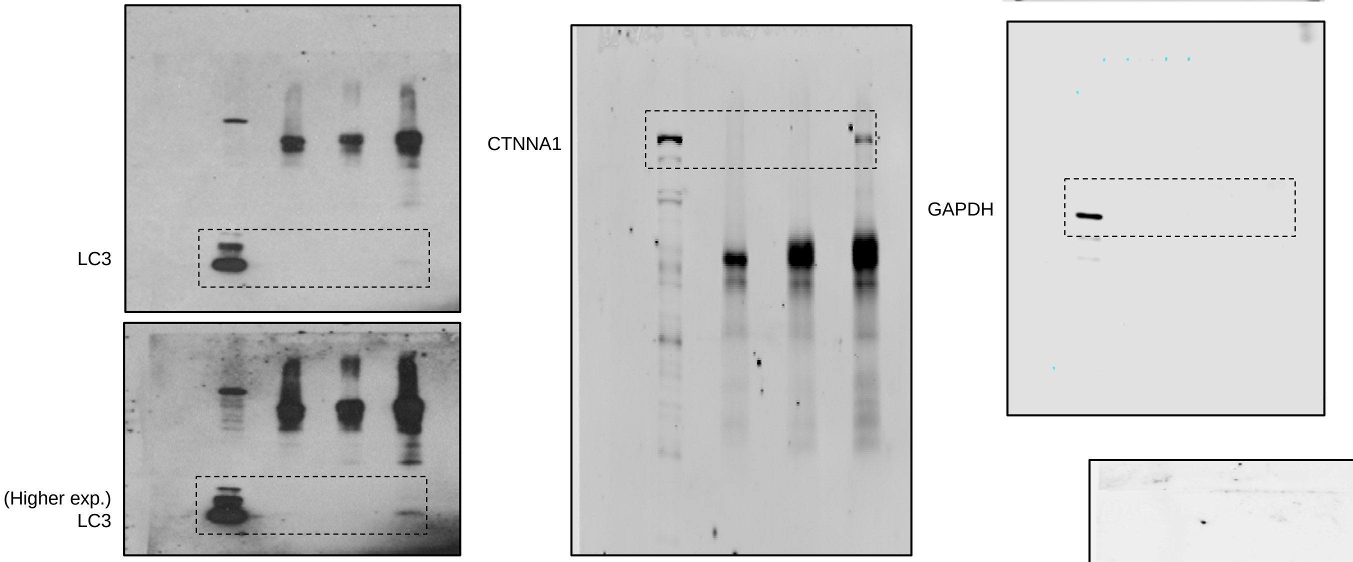

Supplementary Fig. 14c (left)

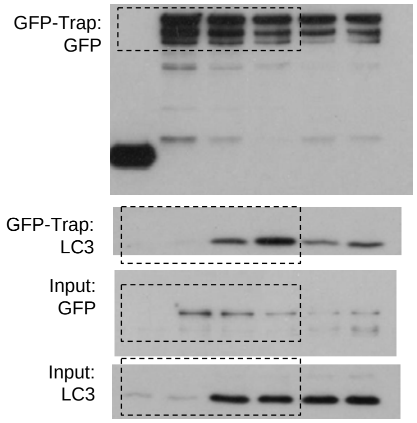

Supplementary Fig. 18 (Nucleus)

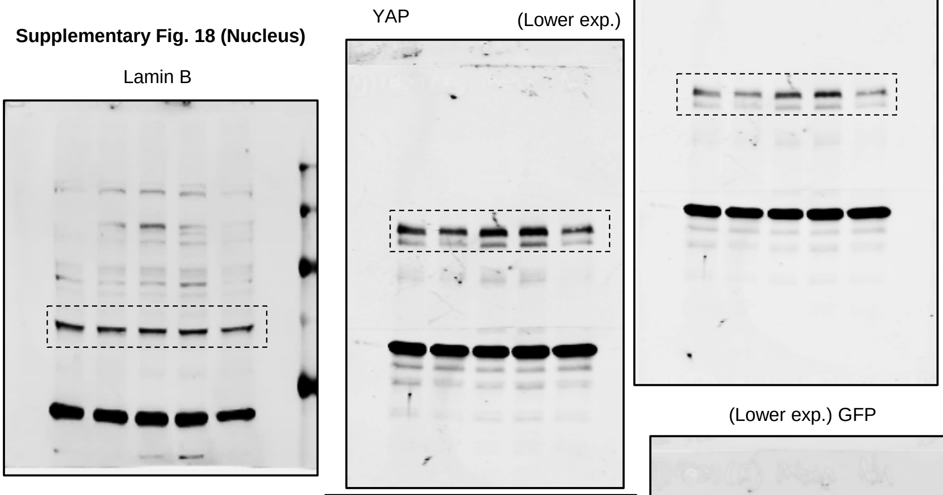

Supplementary Fig. 14c (right)

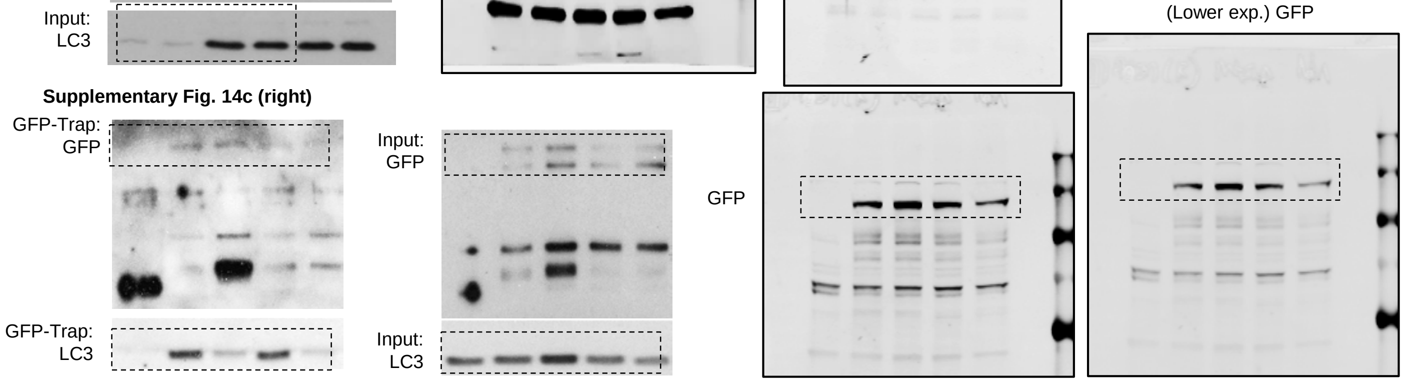

Supplementary Fig. 18 (Cytosol)

YAP

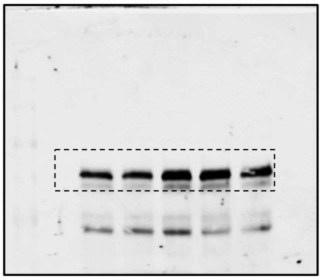

(Lower exp.) YAP

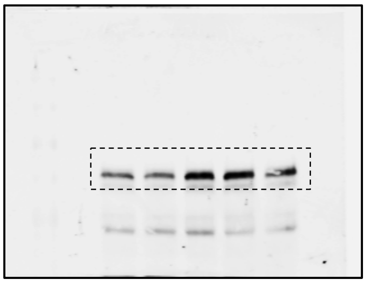

GFP

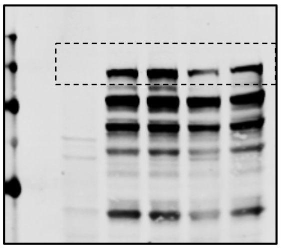

(Lower exp.) GFP

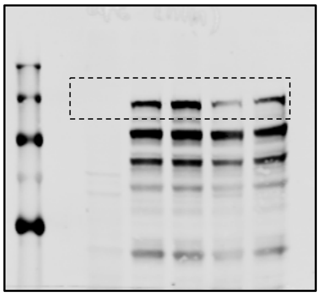

S6

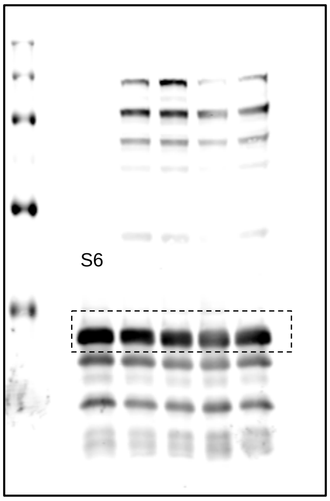

Supplementary Fig. 18 (Total)

(Lower exp.) GFP

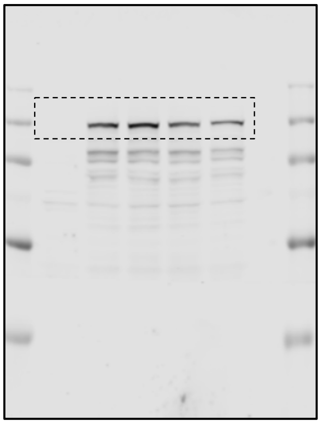

GFP

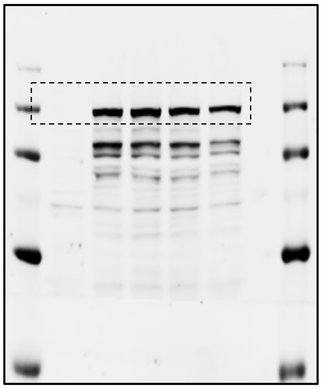

GAPDH

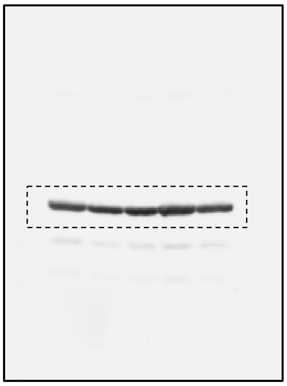

YAP

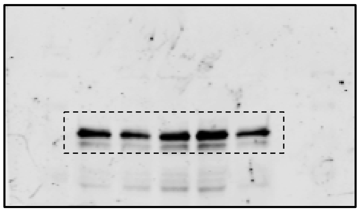

Supplementary Fig. 20a

Flag-Trap: GFP

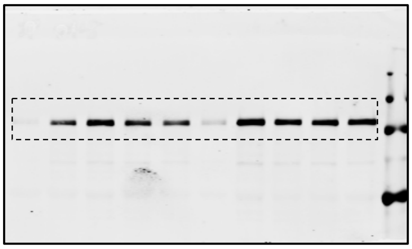

Flag-Trap: (Lower exp.) GFP

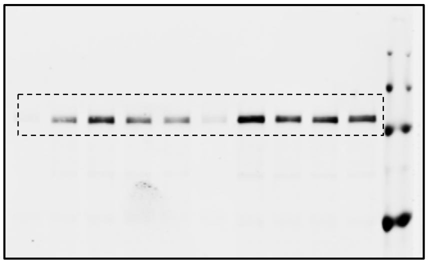

Input: Flag

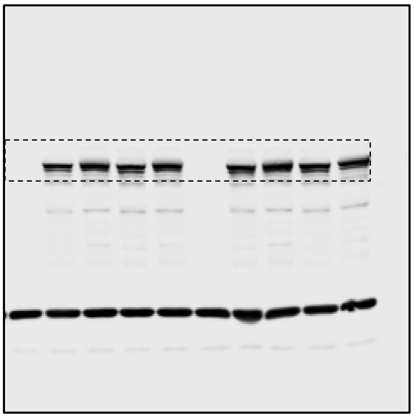

Flag-Trap: Flag

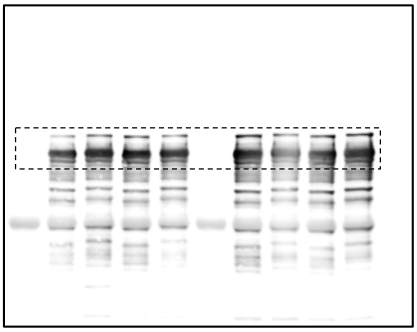

Input: GFP

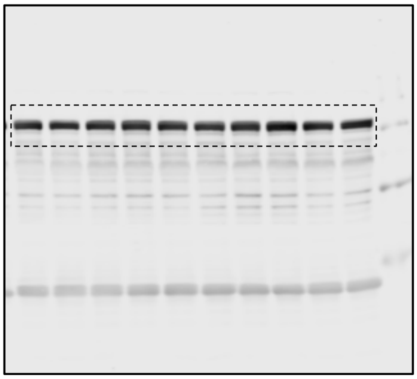

S6

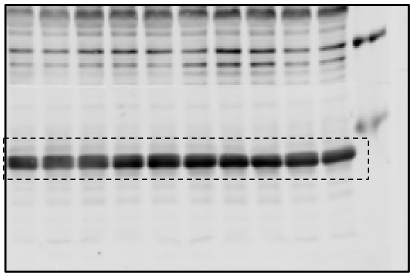

Supplementary Fig. 20a

Flag-Trap: GFP

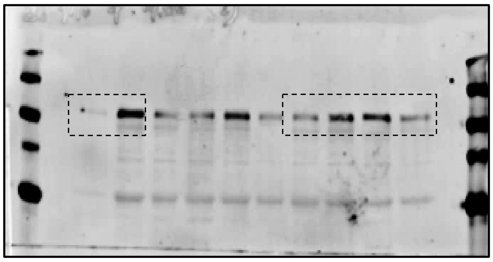

Flag-Trap: (Lower exp.) GFP

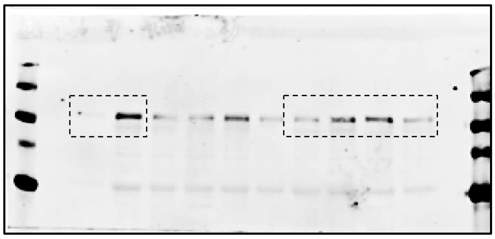

Supplementary Fig. 20b

Flag-Trap: Flag

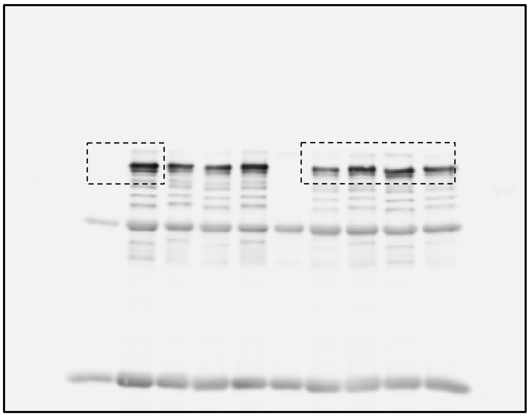

Input: GFP

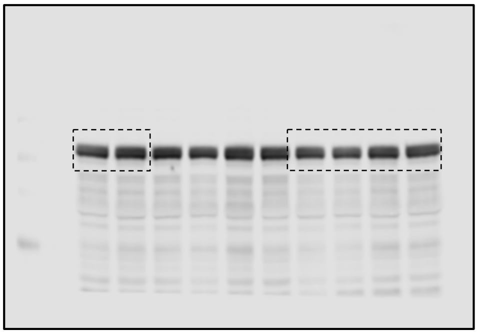

Input: Flag

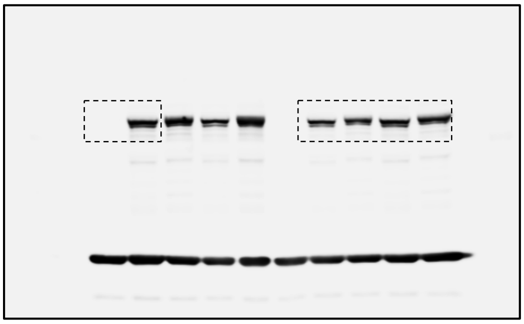

Input: S6

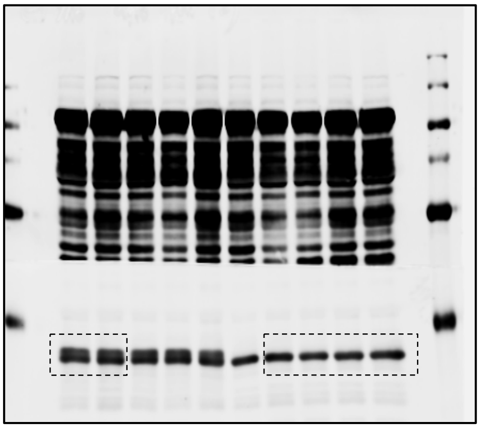

Supplementary Fig. 21f

CTNNA1

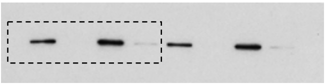

ATG7

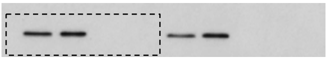

LC3

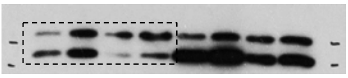

GAPDH

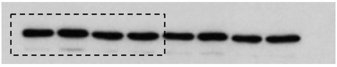

Supplementary Fig. 23b

CTNNA1

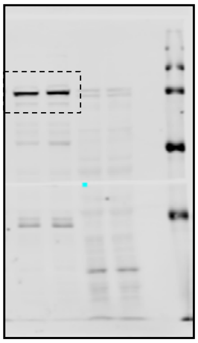

GAPDH

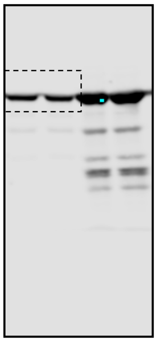

Supplementary Fig. 23a

CTNNA1

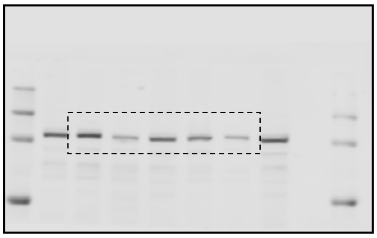

(Higher exp.) CTNNA1

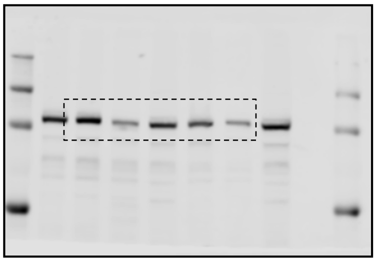

GAPDH

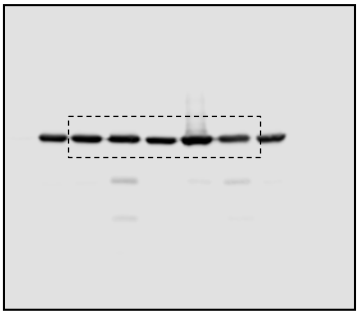

ATG10

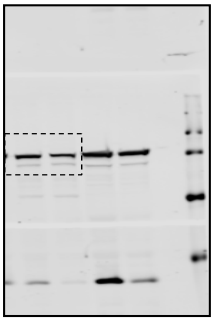

Supplementary Fig. 32 | Full scans of uncropped blots.

Supplementary Table 1: Primer sequences used for mutagenesis.

Mutation	Primers
CTNNA1 L897A-F900A	Fw: 5'- CGTCCAGTGCAGGCCGCGAGCGAGGCCAAAGCCATGGACAGC -3'<br>Rv: 5'- GCTGTCCATGGCTTTGGCCTCGCTCGCGGCCTGCACTGGACG -3'
CTNNA1 Y419A-V422A	Fw: 5'- GAAAGAAGTTAAGGAAGCTGCCCAAGCTTTTCGTGAACAT -3'<br>Rv: 5'- ATGTTCACGAAAAGCTTGGGCAGCTTCCTTAACTTCTTTC -3'
CTNNA1 F511D-V514D	Fw: 5'- ATTACTTCCATCGATGACGACTTGGCTGACTCAGAGAACCAC -3'<br>Rv: 5'- GTGGTTCTCTGAGTCAGCCAAGTCGTCATCGATGGAAGTAAT -3'
CTNNA1 Y619A-I622A	Fw: 5'- ACGCCTCCCGTCTGGTGGCCGACGGCGCCCGGGACATCC -3'<br>Rv: 5'- CGGATGTCCCGGGCGCCGTCGGCCACCAGACGGGAGGCGT -3'
