# Supplementary material for: α-Catenin levels determine direction of YAP/TAZ response to autophagy perturbation
Source: Nat Commun. 2021 Mar 17;12:1703. doi: 10.1038/s41467-021-21882-1 (PMC7969950; doi:10.1038/s41467-021-21882-1)
Supplement: Supplementary file 3 — Description of Additional Supplementary Files [file 41467_2021_21882_MOESM3_ESM.pdf]

Description of Additional Supplementary Files

File Name: Supplementary Data 1
Description: **Datasets of mass-spectrometry analysis of SILAC samples.** Two SILAC experiments were performed in HeLa cells: Experiment A (cells fed with heavy amino-acids were exposed to DMSO, while the cells fed with medium amino-acids were treated with BafA1 at 200 nM for 24 h) and Experiment B (cells fed with medium amino-acids were exposed to DMSO, while the cells fed with light amino-acids were treated with BafA1 at 200 nM for 24 h)
